# Harnessing nature’s pharmacy: investigating natural compounds as novel therapeutics for ulcerative colitis

**DOI:** 10.3389/fphar.2024.1394124

**Published:** 2024-08-14

**Authors:** You Huang, Qiuhong Wu, Sha Li, Xia Lin, Shasha Yang, Rui Zhu, Chaomei Fu, Zhen Zhang

**Affiliations:** ^1^ School of Pharmacy/School of Modern Chinese Medicine Industry, Chengdu University of Traditional Chinese Medicine, Chengdu, China; ^2^ State Key Laboratory of Southwestern Chinese Medicine Resources, Chengdu University of Traditional Chinese Medicine, Chengdu, China

**Keywords:** ulcerative colitis, natural compounds, gut microbiota, intestinal mucosal barrier, intestinal immune responses

## Abstract

**Backgrounds:**

Ulcerative colitis (UC) is a form of chronic inflammatory bowel disease, and UC diagnosis rates continue to rise throughout the globe. The research and development of new drugs for the treatment of UC are urgent, and natural compounds are an important source. However, there is a lack of systematic summarization of natural compounds and their mechanisms for the treatment of UC.

**Methods:**

We reviewed the literature in the databases below from their inception until July 2023: Web of Science, PubMed, China National Knowledge Infrastructure, and Wanfang Data, to obtain information on the relationship between natural compounds and UC.

**Results:**

The results showed that 279 natural compounds treat UC through four main mechanisms, including regulating gut microbiota and metabolites (Mechanism I), protecting the intestinal mucosal barrier (Mechanism II), regulating intestinal mucosal immune response (Mechanism III), as well as regulating other mechanisms (Mechanism Ⅳ) such as cellular autophagy modulation and ferroptosis inhibition. Of these, Mechanism III is regulated by all natural compounds. The 279 natural compounds, including 62 terpenoids, 57 alkaloids, 52 flavonoids, 26 phenols, 19 phenylpropanoids, 9 steroids, 9 saponins, 8 quinonoids, 6 vitamins, and 31 others, can effectively ameliorate UC. Of these, terpenoids, alkaloids, and flavonoids have the greatest potential for treating UC. It is noteworthy to highlight that a total of 54 natural compounds exhibit their therapeutic effects by modulating Mechanisms I, II, and III.

**Conclusion:**

This review serves as a comprehensive resource for the pharmaceutical industry, researchers, and clinicians seeking novel therapeutic approaches to combat UC. Harnessing the therapeutic potential of these natural compounds may significantly contribute to the improvement of the quality of life of patients with UC and promotion of disease-modifying therapies in the future.

## 1 Introduction

Ulcerative colitis (UC) is an idiopathic, chronic, inflammatory bowel disease (IBD) characterized by continuous inflammation starting from the rectum (Hoivik et al., 2013; Conrad et al., 2014; Nanki et al., 2019). World Health Organization has classified UC as a clinically intractable disease. Its global prevalence and incidence have been increasing with time; currently, its incidence and prevalence are 8–10 cases/100,000 subjects and 150–200 cases/100,000 subjects, respectively (da Silva et al., 2014; Ungaro et al., 2017). The annual UC treatment costs (direct and indirect) are estimated to be approximately US$8.1–14.9 billion and €12.5–29.1 billion in the United States and Europe, respectively (Cohen et al., 2010).

UC is primarily treated with medicines, including aminosalicylates, immunomodulators, steroids, and biologics. However, due to potential adverse reactions and reduced efficiency of standard therapies, a comprehensive search for the identification of novel and natural medicines has been initiated to replace or complement present treatment options (Pastorelli et al., 2009; Wan et al., 2014). Many researchers are now turning to natural resources to seek effective compounds that can be used against UC (Cao et al., 2019).

Currently, there are some reviews on natural compounds and UC, such as summarizing some natural compounds or a class of compounds. These studies are significant for finding drugs for UC, but there is still a lack of systematic summaries. Therefore, this study reviews the current progress made in the intervention of natural compounds in UC, and provides a complete overview of natural compounds and their mechanisms of action. More importantly, we hope that such a systematic summary will lead to important natural compounds and mechanisms of action for the treatment of UC. This review serves as a comprehensive resource for the pharmaceutical industry, researchers, and clinicians seeking novel therapeutic approaches to combat UC. Harnessing the therapeutic potential of these natural compounds may significantly contribute to the improvement of the quality of life of patients with UC and promotion of disease-modifying therapies in the future.

## 2 The etiology of UC

**FIGURE 1:**
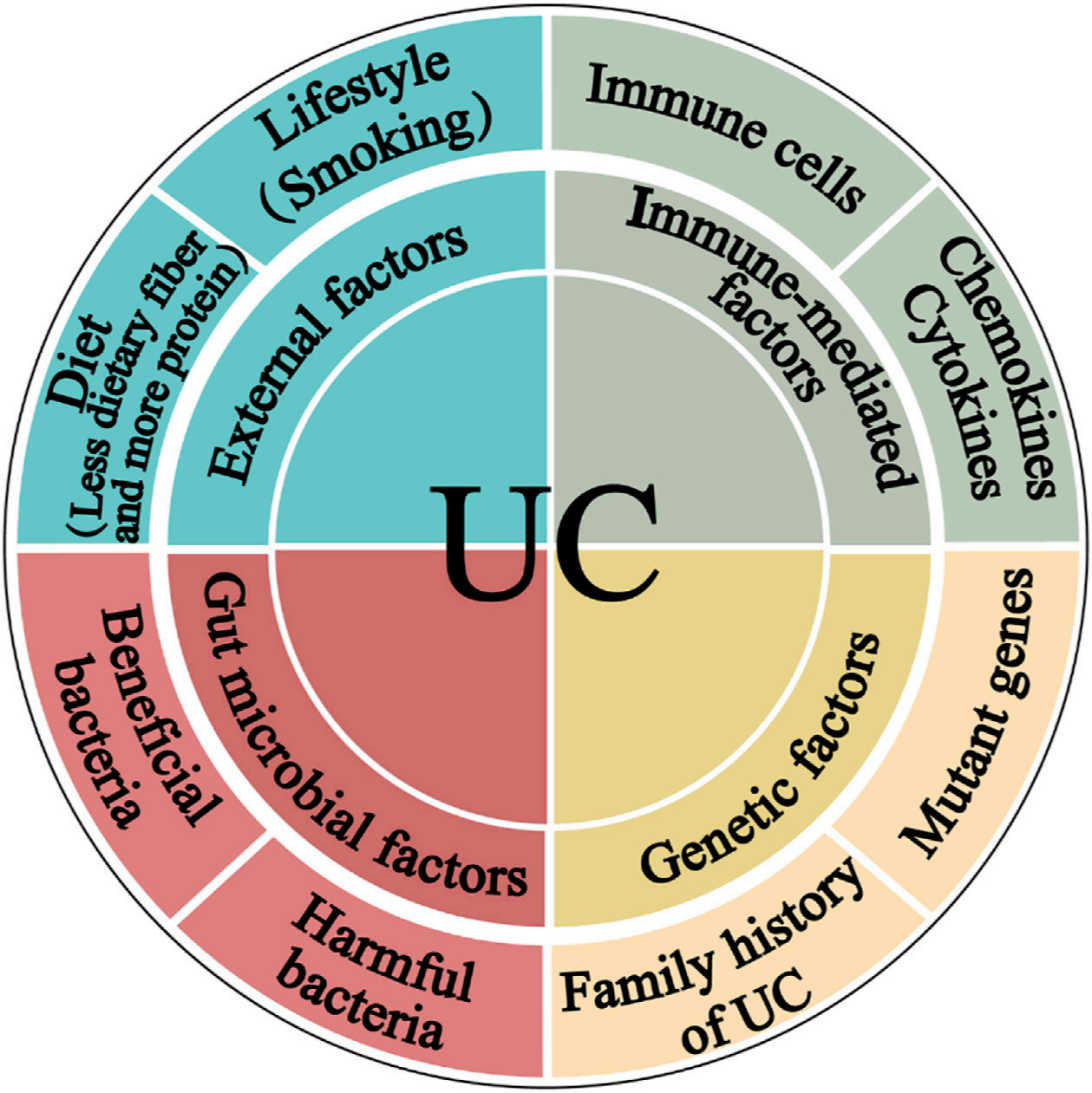
The pathogenesis of ulcerative colitis. The four main components linked to epithelial barrier abnormalities that drive the ulcerative colitis pathogenic mechanism are external factors, immune dysregulation, gut microbiota, and genetic inheritance.

The most accepted hypothesis states that UC pathogenesis comprises complex communications between, external, immunological, and gut microbial factors in a genetically susceptible host (Figure 1) (Abraham et al., 2017; Glassner et al., 2020).

### 2.1 External factors

The diet structure of modern people is constantly changing. People are gradually consuming less dietary fiber, whereas increasing meat, egg, and milk product intake, is the main reason for the increase in the prevalence of UC. Furthermore, although smoking cigarettes is a critical Crohn’s disease (CD) risk factor, quitting it has been linked to UC. According to a meta-analysis, smoking is more protective against UC than not smoking (Mahid et al., 2006). UC individuals who smoked had a milder disease course than non-smokers. UC is harsher for those who stop smoking. It may be mediated by carbon monoxide that can suppress interleukin-10 (IL-10) through a heme oxygenase (HO)-1-dependent pathway in UC mice (Sheikh et al., 2011).

### 2.2 Immune-mediated factors

The immune response is intricately associated with the pathophysiology of UC. The buildup of innate lymphoid cells (ILC), natural killer (NK) cells, macrophages, dendritic cells, neutrophils, and abnormal T and B cells inside the intestinal mucosa, along with the production of chemokines and cytokines that may trigger an inflammatory response. This inflammatory process can lead to the disruption of the intestinal mucosa and ultimately result in the development of UC (Liu Y. et al., 2022).

### 2.3 Gut microbial factors

The gut microbiota directly impacts the maintenance of homeostasis in the intestinal pro-inflammatory and anti-inflammatory responses. Germ-free conditions prevent the development of colitis in genetically susceptible mice (Veltkamp et al., 2001). Moreover, the introduction of proinflammatory bacteria or microbiota from patients with UC into healthy mice can induce inflammation (Ohkusa et al., 2003), while colonization of mice with intestinal microbiota from donors with IBD exacerbates colitis by modulating immune responses (Britton et al., 2019).

### 2.4 Genetic factors

Genetic factors have also been linked with UC. 12% of UC patients have a family history of IBD (Childers et al., 2014). Genome-wide association studies have identified 200 risk loci for IBD to date, with most genes contributing to both UC and CD phenotypes (Jostins et al., 2012; Liu et al., 2015). Examples of loci associated with increased UC susceptibility include human leukocyte antigen and genes associated with barrier function, such as HNF4A and CDH1 (Consortium et al., 2009). In addition, with increasing knowledge about UC pathogenesis, natural compounds have become a research hotspot because of their more efficient application prospects for preventing and mitigating UC occurrence and development.

## 3 The mechanism of natural compounds in intervention UC

**FIGURE 2:**
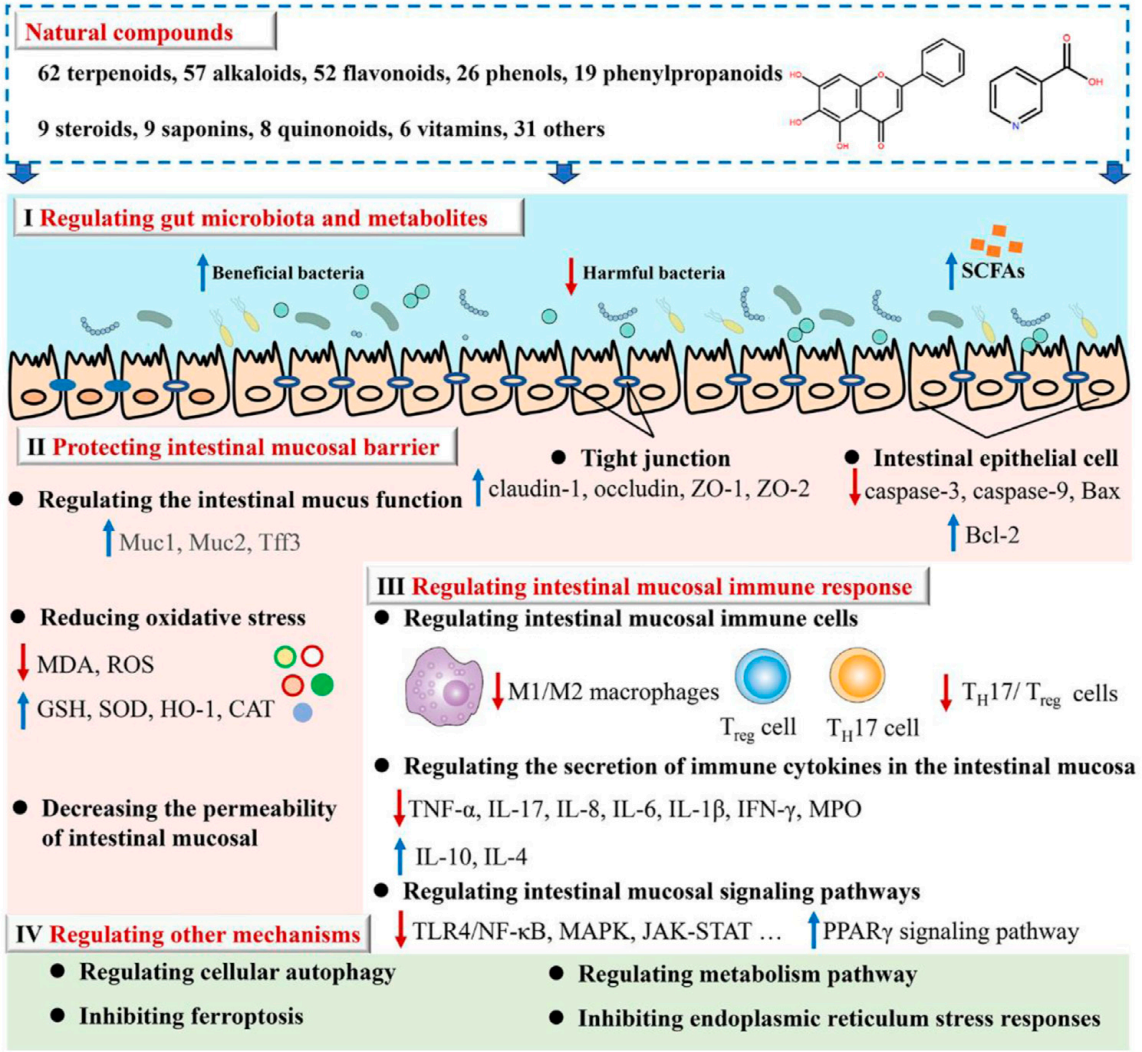
Illustrates how natural compounds intervene in ulcerative colitis.

We reviewed the scientific papers in the databases below from their inception to July 2023 to identify the studies relevant to the mechanism and activity of natural compounds against UC: PubMed, Web of Science, Wanfang Data, and the China National Knowledge Infrastructure. The present study provides a comprehensive summary of 279 natural compounds demonstrated to treat UC through various mechanisms primarily. These mechanisms include regulating gut microbiota and metabolites (Mechanism I), protecting the intestinal mucosal barrier (Mechanism II), regulating intestinal mucosal immune response (Mechanism III), as well as the other mechanisms (Mechanism Ⅳ) such as cellular autophagy modulation and ferroptosis inhibition (as depicted in Figure 2; Supplementary Table S1). It is noteworthy to highlight that Mechanism III is regulated by all natural compounds; Mechanisms II and III can be modulated by at least half of the compounds. Research on these mechanisms may give information on the etiology of UC.

### 3.1 Regulating gut microbiota and metabolites

**FIGURE 3:**
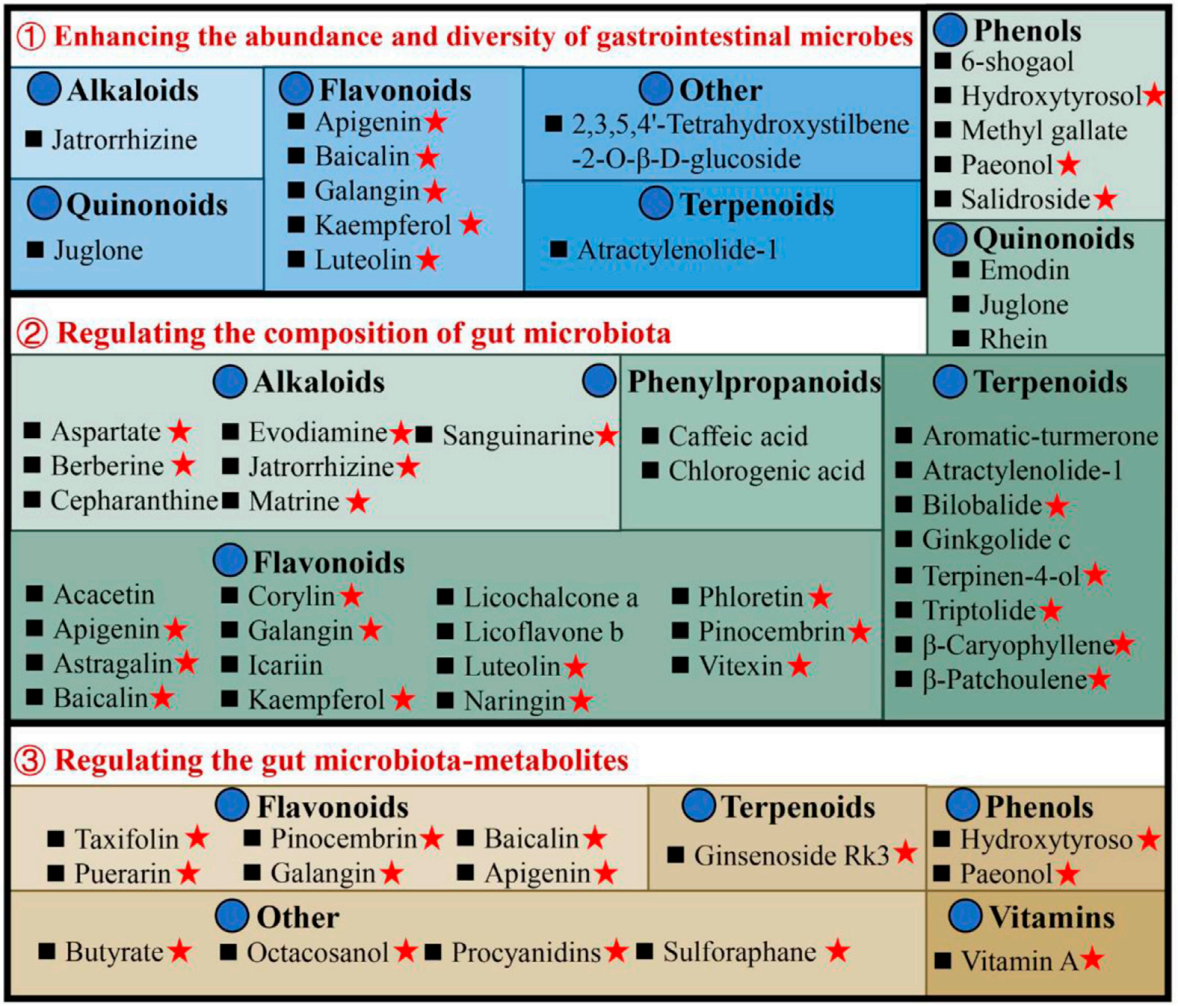
Illustrates the utilization of natural compounds in the management of ulcerative colitis through the modulation of gut microbiota and metabolites. Red pentagrams indicate compounds involved in Mechanism Ⅰ, Ⅱ, and Ⅲ. The natural compounds involved in this paper are shown in Supplementary Table S1.

The available evidence indicates that UC is an increased immune response in the mucosal lining, which is triggered by an imbalance in particular gut bacteria. This condition is defined by an abnormal composition of the microbiota and the presence of bacterial products. According to the data shown in Supplementary Table S1, there has been extensive research conducted on natural compounds to investigate their prebiotic qualities. These compounds have been found to have an impact on the makeup of the microbiota and its metabolites, as well as the prevention of colonization by intestinal pathogens and the reduction of the risk of recurrence of ulcerative colitis, as illustrated in Figure 3.

#### 3.1.1 Enhancing the abundance and diversity of gastrointestinal microbes

Patients with UC exhibit a diminished diversity of gut microbes, and an imbalance in the composition of the microbiome continues throughout the progression of the disease (Fang et al., 2021). Microbial diversity and community richness of species are reflected by Shannon and Simpson’s indexes and abundance-based coverage estimator (ACE) and Chao1 indexes, respectively (Wang et al., 2022). Research shows that atractylenolide Ⅰ (Qu et al., 2022), kaempferol (Qu et al., 2021), and 6-shogaol (Wei et al., 2022) indicated a substantial elevation of the Shannon, Simpson, and Chao1 indexes in the UC mice intestinal flora. Ginkgolide C (Xu et al., 2022), hydroxytyrosol (Miao, 2022), jatrorrhizine (Zhang et al., 2022), luteolin (Li et al., 2021), and sauchinone (Wu et al., 2023) enhanced the diversity and abundance of UC intestinal flora by increasing Chao1 and Shannon indexes. Whereas apigenin (Fu et al., 2022), berberine (Wei et al., 2022), docosapentaenoic acid (Dong et al., 2022a), ginsenoside Rg1 (Cheng et al., 2022; Long et al., 2022), and 2,3,5,4′-tetrahydroxystilbene-2-O-β-D-glucoside (He et al., 2021) elevated the abundance and diversity by Chao1 and ACE indexes upregulation.

#### 3.1.2 Regulating the composition of gut microbiota

The composition of gut microbiota in individuals with UC or animal models has exhibited considerable heterogeneity across different studies. In general, when comparing the microbiota of individuals or animals in good health, it is observed that there is a reduction in the abundance of beneficial bacteria and an increase in the prevalence of harmful bacteria. Seven alkaloids [aspartate (Hu et al., 2022), berberine (Li et al., 2022a), cepharanthine (Wang et al., 2022), evodiamine (Wang et al., 2020), jatrorrhizine (Zhang et al., 2022), matrine (Yao et al., 2021), and sanguinarine (Li et al., 2022b)], 15 flavonoids [acacetin (Ren et al., 2021), apigenin (Fu et al., 2022), astragalin (Peng et al., 2020), corylin (Wang et al., 2023), galangin (Xuan et al., 2020), icariin (Zhang et al., 2021a), kaempferol (Qu et al., 2021), luteolin (Li et al., 2021), licoflavone B (Zhang et al., 2022b), licochalcone A (Zhang et al., 2021), α-mangostin (Gutierrez-Orozco et al., 2014), naringin (Cao et al., 2021), pinocembrin (Yue et al., 2020), phloretin (Wu et al., 2019), and vitexin (Zhang et al., 2022c)], 6 phenols [hydroxytyrosol (Miao, 2022), methyl gallate (Zhou et al., 2022), paeonol (Zheng et al., 2022), prim-O-Glucosylcimifugin (Yin et al., 2022), salidroside (Liu et al., 2023), and 6-shogaol (Wei et al., 2022)], 2 quinonoids [juglone (Hua et al., 2021), rhein (Dong et al., 2022)], 8 terpenoids [atractylenolide-1 (Qu et al., 2022), aromatic-turmerone (Li et al., 2022), bilobalide (Zhang et al., 2021c), β-caryophyllene (Yeom et al., 2022), ginkgolide C (Xu et al., 2022), β-patchoulene (Liu et al., 2020), triptolide (Wu et al., 2020) and terpinen-4-ol (Zhang et al., 2017a)], 2 phenylpropanoids [caffeic acid (Zhang et al., 2016), chlorogenic acid (Niu et al., 2022)] could stimulates the propagation of beneficial bacteria and reduces some pathogenic bacteria. For instance, the administration of berberine in mice with DSS-induced UC has been found to induce a range of protective effects (Zhang et al., 2017; Jiang et al., 2021; Zheng et al., 2021; Li et al., 2022a). These effects include the mitigation of colon inflammation and oxidative stress, restoration of the epithelial barrier’s functionality, and improvement of the gut microenvironment. Specifically, berberine supplementation has been observed to increase the abundance of Bacillibacteria, *Bacteroides fragilis*, Eubacterium, *Lactobacillales*, and *Lactobacillus/Lactococcus*. Conversely, it has been found to decrease the levels of *Akkermansia muciniphila*, *Bacteroides*, *Desulfovibrio*, Enterobacteriaceae, Segmented flamentous bacteria, Verrucomicrobiae, and Verrucomicrobiales.

#### 3.1.3 Regulating the gut microbiota-metabolites

The metabolites of gut microbiota, including tryptophan, bile acids, and short-chain fatty acids (SCFAs), affect UC development. Most of the current research has focused primarily on the effects of SCFAs. The research indicates reduced SCFA-producing bacteria, including *Clostridium clusters IV and XIVb, Faecalibacterium, Leuconostocaceae, Odoribacter*, and *Roseburia* in UC patients (Kostic et al., 2014). Moreover, recently many natural compounds, for instance, apigenin (Fu et al., 2022), baicalin (Zhu et al., 2020), evodiamine (Shen et al., 2019), galangin (Xuan et al., 2020), ginsenoside Rg1 (Long et al., 2022), hydroxytyrosol (Miao, 2022), octacosanol (Miao et al., 2022), pinocembrin (Hu et al., 2019), paeonol (Zheng et al., 2022), procyanidins (Huang et al., 2022), sulforaphane (Zhang et al., 2020), and vitamin A (Pang et al., 2021), could increase SCFA-producing bacteria in UC models. For example, taxifolin can ameliorate DSS-induced colitis by altering gut microbiota to increase the production of SCFAs (Li et al., 2022). Furthermore, SCFAs function by stimulating G-protein-coupled receptors (GPCR) and suppressing histone deacetylases (Kostic et al., 2014). It is reported that taxifolin can increase the level of GPR41 and GPR43 in the colon, and increase the level of the content of SCFAs, thereby reducing DSS-induced intestinal inflammatory reaction and protecting the intestinal mucosa (Li et al., 2022).

The present study has specifically examined the impact of pharmaceutical substances on the composition and diversity of the gastrointestinal microbiota. However, the gut microbiota exerts a significant influence on the chemical alteration, pharmacological action, and metabolic mechanisms of natural compounds (Zhao et al., 2022a). Certain gut microorganisms possess the ability to break down and convert organic substances, resulting in the production of metabolites and functional chemicals that exhibit physiological actions that are not naturally generated by the host organism (Koppel et al., 2017). There is currently a significant amount of research being dedicated to comprehending the distinct ways in which microorganisms alter natural products and the consequent effects of these metabolites on the health of the host organism (Luca et al., 2020). This is a matter that warrants further investigation in our research.

### 3.2 Protecting intestinal mucosal barrier

**FIGURE 4:**
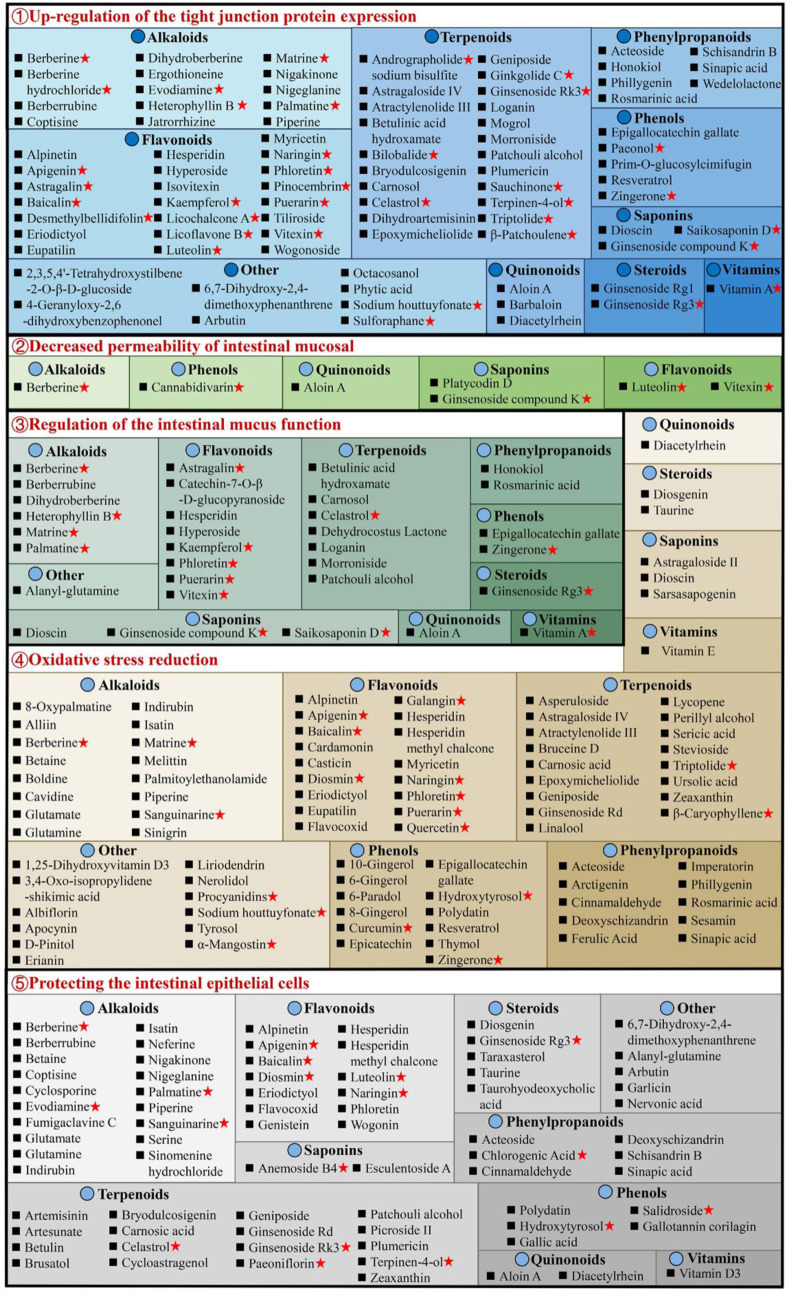
Natural compounds against ulcerative colitis *via* intestinal mucosal barrier protection. Red pentagrams indicate compounds involved in Mechanism Ⅰ, Ⅱ, and Ⅲ. The natural compounds involved in this paper are shown in Supplementary Table S1.

The intestinal mucosal barrier damage is a crucial UC characteristic (Ungaro et al., 2017). Complete healing of intestinal mucosa is the most desired goal in UC treatment (Du et al., 2020). As shown in Supplementary Table S1, natural compounds can improve the barrier function of the UC mucosa through multiple perspectives, these include upregulation of the expression of tight junction protein, reduction in the intestinal mucosal, permeability, regulation of the intestinal mucus function, reduction of oxidative stress, and protection of the intestinal epithelial cells (Figure 4).

#### 3.2.1 Upregulation of the tight junction protein expression

The tight junctions (TJs) are present between epithelial cells in the junctions’ apical region and comprise multiple proteins, such as claudins, junctional adhesion molecules, occludin, and tricellulin (Pan et al., 2023). TJs are an intestinal mucosal mechanical barrier required for the maintenance of intestinal epithelium integrity and intestinal mucosal permeability by modulating ions and molecules’ entrance into the paracellular channels (Suzuki, 2013). The destruction or reduction of TJ proteins can disrupt the gastrointestinal mucosal barrier, causing UC and other intestinal disorders. However, some natural compounds, such as 8 alkaloids [berberine hydrochloride (Zhu et al., 2019a), berberrubine (Yu et al., 2018), coptisine (Wang et al., 2021a), dihydroberberine (Li et al., 2021), evodiamine (Shen et al., 2019), matrine (Yan et al., 2020), nigakinone (Liu et al., 2023), and piperine (Guo et al., 2020)], 8 flavonoids [apigenin (Fu et al., 2022), kaempferol (Qu et al., 2021), licochalcone A (Zhang et al., 2021), licoflavone B (Zhang et al., 2022b), phloretin (Zhang Z. et al., 2019), pinocembrin (Hu et al., 2019), puerarin (Wu et al., 2020), and wogonoside (Huang et al., 2020)], 2 phenylpropanoids [honokiol (Wang and Wang, 2022), sinapic acid (Qian et al., 2020)], 5 terpenoids [carnosol (Xu et al., 2022b), ginsenoside Rk3 (Tian et al., 2020), patchouli alcohol (Wu et al., 2020), plumericin (Rapa et al., 2021), and sauchinone (Wu et al., 2023)], arbutin (Zhang et al., 2021), sodium houttuyfonate (Cheng et al., 2023), can promote TJ proteins expression in UC animals, such as claudin-1, occludin, and zona occludens 1 (ZO-1), thus efficiently prevent the paracellular permeability disruption. Additionally, berberrubine (Yu et al., 2018), coptisine (Wang et al., 2021a), dihydroberberine (Li et al., 2021), patchouli alcohol (Wu et al., 2020), and palmatine (Zhang et al., 2018) promote ZO-2 protein levels in UC animals. Multiple researches are investigating UC alleviation by alkaloids and flavonoids, which upregulate TJ proteins.

#### 3.2.2 Decreased permeability of intestinal mucosal

The permeability of the intestinal mucosa controls the transport of molecular substances across the epithelium of the intestinal mucosa by the process of simple diffusion. Increased mucosal permeability (Nakarai et al., 2012) has been reported in UC patients (Wang et al., 2022), allowing the entrance of intestinal pathogens as well as their toxic metabolites in the liver, lymph, peripheral tissues, and blood, causing enhanced oxidative stress and inflammation. Intestinal permeability allows accurate, direct, and quantitative evaluation of the colonic epithelial barrier (Huang et al., 2016). Generally, FITC-dextran (fluorescein isothiocyanate dextran) permeability is utilized for the elucidation of epithelium integrity. It has recently been revealed that after taking FITC-dextran orally, the serum of DSS mice had markedly increased FITC-dextran levels (Zhang et al., 2022c). Interestingly, berberine (Zheng et al., 2021), cannabidivarin (Pagano et al., 2019), dioscin (Cai et al., 2021), ginsenoside compound K (Wang et al., 2022), luteolin (Xie et al., 2022), platycodin D (Guo et al., 2021), vitexin (Zhang et al., 2022c), and wogonoside (Huang et al., 2020), can decrease serum FITC-dextran level in UC animals.

#### 3.2.3 Regulation of the intestinal mucus function

The structure of the intestinal mucus is composed of the glycoprotein network containing host-specific glycan that prevents the interaction of bacteria and epithelium, inhibits infection, and modulates the balance between exogenous stimulation and immune function (Johansson et al., 2014). Intestinal mucosal layer dysfunction compromises intestinal epithelium integrity and enhances pathogenic susceptibility. The main intestinal mucosal component is mucin (Johansson et al., 2011). During active UC, there are decreased goblet cells in the colon epithelium, the protective mucus layer thickness reduces, and the mucus levels in mucin, glycosylation, and phosphatidylcholine alters. Alterations in the levels of colon proteins, such as trefoil factor 3 (Tff3), mucin 1 (Muc1), and Muc2, increase susceptibility to chronic inflammation, indicating mucins’ importance in intestinal barrier repair. The research suggests that 7 flavonoids [astragalin (Peng et al., 2020), catechin-7-O-β-D-glucopyranoside (Kook et al., 2015), hyperoside (Cheng et al., 2021), kaempferol (Park et al., 2012), puerarin (Wu et al., 2020), phloretin (Wu et al., 2019), and vitexin (Zhang et al., 2022c)], 8 alkaloids [berberrubine (Yu et al., 2018), berberine (Dong et al., 2022c), dihydroberberine (Li et al., 2021), evodiamine (Wang et al., 2020), heterophyllin B (Chen et al., 2022), matrine (Yan et al., 2020), palmatine (Zhang et al., 2018), and tryptophan (Islam et al., 2017)], 8 terpenoids [betulinic acid hydroxamate (Prados et al., 2021), carnosol (Xu et al., 2022b), celastrol (Li et al., 2022), dehydrocostus lactone (Zhou et al., 2020), ginsenoside Rg3 (Liu et al., 2023), loganin (Yuan et al., 2020), morroniside (Yuan et al., 2020), and patchouli alcohol (Wu et al., 2020)], 2 phenols [epigallocatechin gallate (Diwan and Sharma, 2022), zingerone (Zhang et al., 2022)], aloin A (Jiang et al., 2022), alanyl-glutamine (Hou et al., 2013), rosmarinic acid (Formiga et al., 2020), saikosaponin D (Li et al., 2020) and vitamin A (Pang et al., 2021) can effectively enhance the colon tissue expression of mucus-linked mucins and Tff3 in the UC mice to improve the function of colonic barrier.

#### 3.2.4 Oxidative stress reduction

Increased oxidative stress causes colonic mucosal barrier activity loss and a marked reduction in TJ proteins, thus enhancing the risk for the development of UC. In the intestine, inflammation and oxidative stress together disrupt the mucosal redox balance and promotes apoptosis of intestinal epithelial cell (IEC) (Seo et al., 2014). It has been indicated that bruceine D (Dou et al., 2018) and casticin (Ma et al., 2018) reduce malondialdehyde (MDA) and reactive oxygen species (ROS) and enhances glutathione (GSH) and superoxide dismutase to alleviate the damage caused by oxidative stress damage in colon tissues and UC symptoms in animals. Stevioside can alleviate colonic epithelium oxidative damage by UC, including ROS reduction and intestinal mucosal GSH consumption and elevating the enzyme activity of catalase (CAT), GSH (Mostafa et al., 2020), and heme oxygenase-1 (HO-1). Furthermore, there are many compounds, including atractylenolide III (Han et al., 2022), astragaloside IV (Zhong et al., 2022), asperuloside (Chen et al., 2021), alpinetin (Tan and Zheng, 2018), acteoside (Guo et al., 2022), brusatol (Zhou et al., 2018), bruceine D (Dou et al., 2018), betaine (Chen et al., 2022), berberine (Zhang et al., 2017), baicalin (Yao et al., 2016), carnosic acid (Yang et al., 2017), D-pinitol (Lin et al., 2021), epoxymicheliolide (He et al., 2022), geniposide (Yang et al., 2020), galangin (Sangaraju et al., 2019), 6-gingerol (Ajayi et al., 2018), hydroxytyrosol (Elmaksoud et al., 2021), isatin (Socca et al., 2014), imperatorin (Luo and Luo, 2021), lycopene (Tekeli et al., 2018; Li et al., 2021; Yin et al., 2023), naringenin (Al-Rejaie et al., 2013), 8-oxypalmatine (Cheng et al., 2022), and 3,4-Oxo-isopropylidene-shikimic acid (Xing et al., 2012), puerarin (Jeon et al., 2020), sesamin (Bai et al., 2019), syringic acid (Fang et al., 2019), stevioside (Alavala et al., 2019; Mostafa et al., 2020), sinigrin (Kotipalli et al., 2023), sinapic acid (Qian et al., 2020), tyrosol (Guvenc et al., 2019), vitamin C (Yan et al., 2015), wogonin (Zhou et al., 2022), et al. that decrease colonic epithelium oxidative damage by UC.

#### 3.2.5 Protecting the intestinal epithelial cells

The IECs have rapid renewal capability (Krndija et al., 2019), ensuring normal digestion and barrier activity, and are based on non-inflammatory apoptosis. At UC onset, IEC travels to the damaged area to maintain the intestinal barrier’s integrity (Maria-Ferreira et al., 2018). However, the excessive apoptosis and uncontrolled IEC inflammation are primarily responsible for impaired intestinal mucosal barrier activity in UC. Caspase is the most critical protease associated with apoptosis; Bax and Bcl-2 are essential apoptosis modulatory genes. Some natural compounds, such as 5 alkaloids [berberine (Jia et al., 2020), coptisine (Wang et al., 2021a), indirubin (Gao et al., 2018), isatin (Gao et al., 2018), and palmatine (Zhang et al., 2018)], 3 flavonoids [baicalin (Shen et al., 2019b), hesperidin (Shafik et al., 2019), and wogonin (Zhou et al., 2022)], 2 phenols [hydroxytyrosoland (Elmaksoud et al., 2021), polydatin (Lv et al., 2018)], 4 phenylpropanoids [acteoside (Guo et al., 2022), chlorogenic acid (Gao et al., 2019), deoxyschizandrin (Zhang et al., 2016; Yu and Qian, 2021), and sinapic acid (Shahid et al., 2022)], 3 steroids [taraxasterol (Che et al., 2019), taurine (Giris et al., 2008), and taurohyodeoxycholic acid (Laukens et al., 2014)], 4 terpenoids [cycloastragenol (Bagalagel et al., 2022), plumericin (Rapa et al., 2021), paeoniflorin (Gu et al., 2017), and patchouli alcohol (Qu et al., 2017)] and arbutin (Zhang et al., 2021), diacetylrhein (Zohny et al., 2022), glutamate (Li et al., 2014), and nervonic acid (Yuan et al., 2023), have indicated UC improvement, decreasing the expression of Bax, caspase-3, and caspase-9, whereas increasing Bcl-2 in epithelial cells. Meanwhile, anemoside B4 (Zhang et al., 2021), bryodulcosigenin (Li et al., 2022), and berberrubine (Yu et al., 2018) could decrease the ratio of Bax/Bcl-2 and caspase-3, while artesunate (Yin et al., 2020) increasing the ratio of Bcl-2/Bax and decreasing caspase-3. Diosgenin (Tang et al., 2020) can protect against colonic apoptosis by downregulating the Bax/Caspase-1 pathway. In addition, cyclosporine protects against epithelial apoptosis linked with increased tumor growth factor-β-related signaling (Satoh et al., 2009).

### 3.3 Regulating intestinal mucosal immune response

**FIGURE 5:**
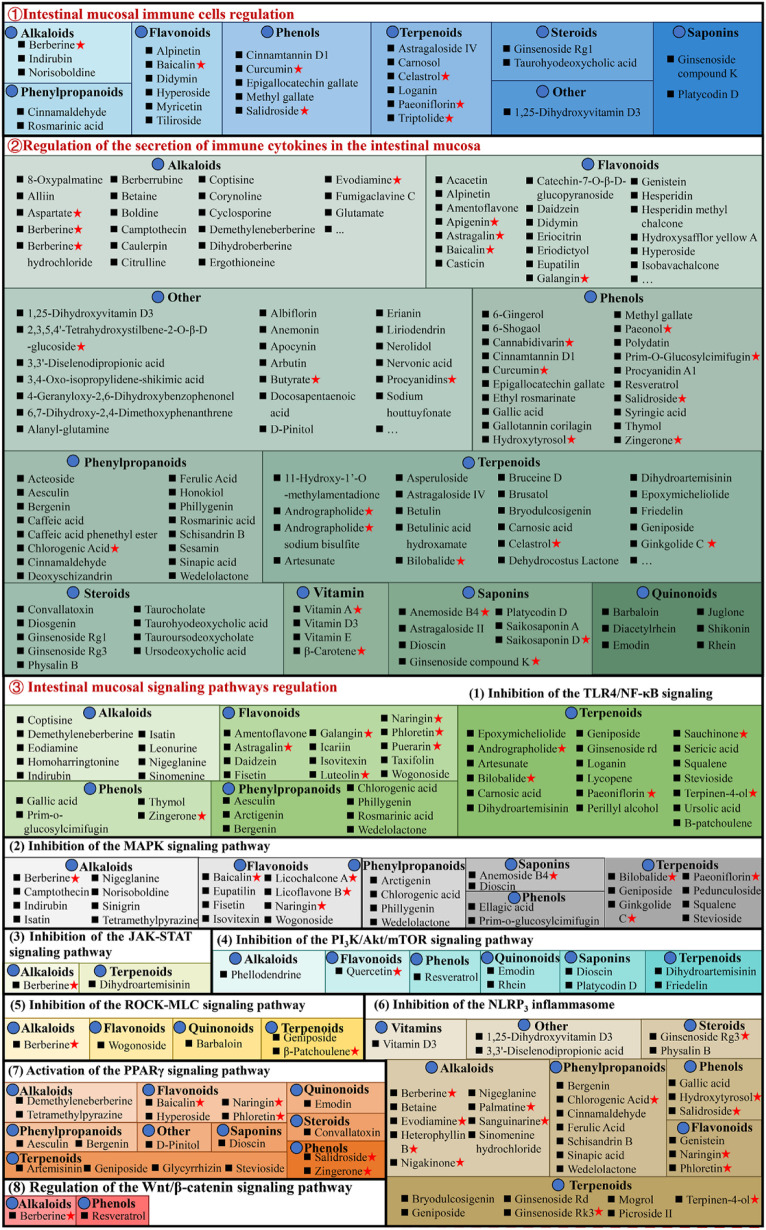
Natural compounds against ulcerative colitis *via* regulation of intestinal mucosal immune response. Red pentagrams indicate compounds involved in Mechanism Ⅰ, Ⅱ, and Ⅲ. The natural compounds involved in this paper are shown in Supplementary Table S1.

The intestinal mucosal immunological disorder is the essential factor for UC pathogenesis, characterized by innate immune system alterations, adaptive immune system activation, increased pro-inflammatory mediators, and anti-inflammatory signals inhibition, causing chronic intestinal inflammation. Currently, 283 natural compounds have been indicated to improve mucosal immune response in UC, primarily by regulating cytokine, inflammatory signaling pathways, and immune cells as shown in Figure 5.

#### 3.3.1 Regulating intestinal mucosal immune cells

Various cell types, including antigen-presenting (dendritic cells and macrophages) and effector and regulatory T cells, are critically linked with UC pathogenesis, as they promote or inhibit inflammation. Macrophages are essential for intestinal homeostasis and the pathology of IBD. Generally, persistent M1 macrophage activation causes excessive stimulation of pro-inflammatory cytokines to release, causing an imbalance of colonic homeostasis and barrier disruption (Bain et al., 2013). Whereas M2 macrophages stimulate anti-inflammatory cytokines to alleviate UC progression (Formentini et al., 2017). It has been reported that didymin (Lv et al., 2021), 1,25-dihydroxyvitamin D3 (Cao et al., 2020), ginsenoside Rg1 (Long et al., 2022), loganin (Liu et al., 2020), methyl gallate (Zhou et al., 2022), platycodin D (Guo et al., 2021), triptolide (Tang et al., 2020), and tiliroside (Zhuang et al., 2021) suppresses M1 macrophages activation and promote M2 macrophages, thereby alleviating UC. In addition, baicalin (Zou et al., 2015), cinnamtannin D1 (Yang et al., 2022), curcumin (Wang et al., 2022), cinnamaldehyde (Qu et al., 2021), epigallocatechin gallate (Xu et al., 2015), ginsenoside compound K (Wang et al., 2022), hyperoside (Cheng et al., 2021), paeoniflorin (Zheng et al., 2020), and salidroside (Liu et al., 2023) can ameliorate chronic intestinal inflammation in UC, and its mechanism that promote intestinal mucosal immune imbalance, thereby regulating Th17/Treg balance.

Interestingly, these compounds are mainly phenols. The endoscopic findings consistently indicate that supplementation with phenols has demonstrated benefits in individuals with IBD. However, to acquire a more comprehensive understanding of the influence of phenols, it is necessary to conduct long-term trials that incorporate both clinical and mechanistic investigations (Hagan et al., 2021).

#### 3.3.2 Regulating the secretion of immune cytokines in the intestinal mucosa

In UC patients’ intestines, increased pro-inflammatory cytokines cause persistent mucosal inflammation and are directly linked with UC pathogenesis. The mucosal immune system is the primary factor affecting intestinal injury and inflammation, and with cytokines, it modulates inflammation (Ardizzone and Bianchi Porro, 2005). Therefore, cytokines are a logical UC target that can be modulated by specific inhibitors. The literature has indicated that chemokine ligand 5 (CCL5), cyclooxygenase (COX-2), IL-1β, IL-6, IL-8, IL-17, interferon-γ (IFN-γ), inducible nitric oxide synthase (iNOS), myeloperoxidase (MPO), nitric oxide (NO), and tumor necrosis factor alpha (TNF-α) expression enhanced in UC animal models, while anti-inflammatory cytokines secretion such as Arg-1, IL-10, and IL-4 decreased. Most natural compounds compiled here can regulate these cytokines to treat UC. Taurohyodeoxycholic acid (TA), a natural 6α-hydroxylated bile acid with hydrophilic properties, is the main component of traditional Chinese medicine (TCM) *Pulvis Fellis Suis*. TA can modulate multiple cytokines in UC, including CXC motif chemokine ligand 2 (Cxcl2), IL-1β, IL-4, IL-6, IL-10, IL-17A, IL-21, IL-22, IFN-γ, MPO, and TNF-α for maintaining the immune balance of the body (He et al., 2011; Laukens et al., 2014; Lv et al., 2023).

#### 3.3.3 Regulating intestinal mucosal signaling pathways

The pathogenesis of UC is associated with multiple complex inflammatory signaling pathways. Natural compounds directly or indirectly interact with the immune system, stimulating different molecular and cellular pathways and producing anti-inflammatory effects. Therefore, UC prevention and therapy by natural molecules regulate one or more complicated signaling pathways.

##### 3.3.3.1 Inhibition of the TLR4/NF-κB signaling pathway

NF-κB is a transcription factor that stimulates inflammatory cytokines’s genetic transcription and is linked with multiple inflammatory diseases. Physiologically, inactive NF-κB interacts with cytoplasmic inhibitor protein IkappaB (IκB). During inflammation, IκB undergoes phosphorylation and degradation, dissociating NF-κB and translocating it from the cytoplasm to the nucleus, thereby activating downstream gene transcription, such as pro-inflammatory cytokines and iNOS. Therefore, NF-κB inhibition is an efficient strategy to prevent UC and inflammatory cytokines release in UC patients. It is reported that 14 alkaloids [corynoline (Zhang et al., 2023), cavidine (Niu et al., 2015), caulerpin (Lucena et al., 2018), coptisine (Wang et al., 2021a), demethyleneberberine (Zhao et al., 2022b), evodiamine (Shen et al., 2019), homoharringtonine (Liu et al., 2022), isatin (Gao et al., 2018), indirubin (Gao et al., 2018), leonurine (Zheng et al., 2021), melittin (Ahmedy et al., 2020), nigeglanine (Gao et al., 2019), platycodin D (Guo et al., 2021), and sinomenine (Zhou et al., 2023)], 16 flavonoids [astragalin (Peng et al., 2020), and amentoflavone (Sakthivel and Guruvayoorappan, 2013), baicalin (Feng et al., 2014), daidzein (Shen et al., 2019c), eupatilin (Zhou et al., 2018), euptailin (Zhou et al., 2018), fisetin (Sahu et al., 2016), galangin (Sangaraju et al., 2019), icariin (Zhang et al., 2021a), licochalcone A (Liu et al., 2018), luteolin (Li et al., 2021), α-mangostin (You et al., 2017), naringin (Cao et al., 2018), puerarin (Jeon et al., 2020), phloretin (Zhang et al., 2019), and taxifolin (Li W. et al., 2022)], 6 phenols [epicatechin (Zhang et al., 2016), gallic acid (Zhu et al., 2019b), polydatin (Yao et al., 2011), prim-*o*-glucosylcimifugin (Yin et al., 2022), thymol (Chamanara et al., 2019), and zingerone (Zhang et al., 2022)], 7 phenylpropanoids [arctigenin (Wu et al., 2014), aesculin (Tiana et al., 2019), bergenin (Wang et al., 2018), caffeic acid (Zhang et al., 2016), chlorogenic acid (Zeng et al., 2020), phillygenin (Xue et al., 2023), and wedelolactone (Wei et al., 2017)], 25 terpenoids [astragaloside IV (Wu and Chen, 2019), astragalin (Peng et al., 2020), asperuloside (Chen et al., 2021), artesunate (Chen et al., 2019), bilobalide (Zhang et al., 2021c), brusatol (Zhou et al., 2018), carnosic acid (Yang et al., 2017), β-carotene (Zhu et al., 2021), diosgenin (Tang et al., 2020), dihydroartemisinin (Li N. et al., 2019), epoxymicheliolide (He et al., 2022), geniposide (Yang et al., 2020), ginkgolide C (Xu D. et al., 2022), ginsenoside Rd (Qu et al., 2022), patchouli alcohol (Wu et al., 2020), parthenolide (Zhao et al., 2012), picroside II (Yao et al., 2022), paeoniflorin (Gu et al., 2017), rographolide (Zhang et al., 2020), stevioside (Alavala et al., 2019), squalene (Sanchez-Fidalgo et al., 2015), sericic acid (Lifei et al., 2023), sauchinone (Wu et al., 2023), terpinen-4-ol (Zhang et al., 2017a), and ursolic acid (Liu et al., 2016)], and 2 steroids [convallatoxin (Li et al., 2019), physalin B (Zhang et al., 2020)], 2 aponins [dioscin (Cai et al., 2021), saikosaponin A (Zhou et al., 2019)] and albiflorin (Wang et al., 2023), erianin (Dou et al., 2020), 4-geranyloxy-2,6-dihydroxybenzophenonel (Wang et al., 2023), liriodendrin (Zhang et al., 2017c), nervonic acid (Yuan et al., 2023), and vitamin C (Kondo et al., 2019) have been indicated to improve UC related systemic symptoms by suppressing NF-κB inflammatory signaling pathway.

Furthermore, the Toll-like receptor 4 (TLR4) is an essential signaling pathway associated with colon inflammation (Rashidian et al., 2020). As an innate immune receptor, TLR4 is activated during inflammation after gut pathogen-associated molecular patterns (PAMPs) recognition, conformation alterations, and dimerization. Activated TLR4 is then recruited at aptamer, activating NF-κB. Much research suggests that colitis is linked with excessive activation of the TLR4/NF-κB signaling pathway (Rashidian et al., 2016; Liu et al., 2017). It is reported that the baicalin (Cui et al., 2014; Feng et al., 2014), berberine (Zhang et al., 2011), cinnamaldehyde (Tan et al., 2023), deoxyschizandrin (Zhang et al., 2016), eriodictyol (Hu et al., 2021), emodin (Xu et al., 2021), honokiol (Wang et al., 2022), hypaconitine (Zhang et al., 2011), hydroxysafflor yellow A (Feng et al., 2022), honokiol (Wang et al., 2022), methyl gallate (Zhou et al., 2022), naringenin (Dou et al., 2013), perillyl alcohol (Puppala et al., 2022), vitexin (Duan et al., 2020), and vitexin (Duan et al., 2020) have been linked with the inhibition of the TLR4/NF-κB signaling pathway and inflammatory cytokines, thereby exerting anti-UC effect.

##### 3.3.3.2 Inhibition of the MAPK signaling pathway

MAPKs family comprises evolutionarily conserved serine/threonine protein kinases, which regulate cellular pathways, such as inflammation-related genes. Among these, stimulation of extracellular-signal-regulated kinases 1/2(ERK-1/2), c-Jun N-terminal kinase (JNK), and p38 kinase (p38) promotes cell apoptosis and aggravates intestinal inflammation. Much research has been published indicating that natural compounds, 7 alkaloids [camptothecin (Wang et al., 2021b), isatin (Gao et al., 2018), indirubin (Gao et al., 2018), melittin (Ahmedy et al., 2020), nigeglanine (Gao et al., 2019), sinigrin (Kotipalli et al., 2023), and tetramethylpyrazine (He et al., 2012)], 5 flavonoids [baicalin (Liang et al., 2019), eupatilin (Zhou et al., 2018), licoflavone B (Zhang et al., 2022b), licochalcone A (Zhang et al., 2021), and naringin (Cao et al., 2018)], 8 terpenoids [bilobalide (Zhang et al., 2021c), ginkgolide C (Xu et al., 2022), geniposide (Lu et al., 2021), pedunculoside (Liu et al., 2020), squalene (Sanchez-Fidalgo et al., 2015), stevioside (Alavala et al., 2019), squalene (Sanchez-Fidalgo et al., 2015), and ursolic acid (Sheng et al., 2021)], 4 phenylpropanoids [arctigenin (Wu et al., 2014), chlorogenic acid (Gao et al., 2019), phillygenin (Xue et al., 2023), and wedelolactone (Wei et al., 2017)], albiflorin (Wang et al., 2023), atractylodin (Qu et al., 2021), β-carotene (Zhu et al., 2021), dioscin (Cai et al., 2021), α-mangostin (You et al., 2017), nervonic acid (Yuan et al., 2023), and prim-*O*-Glucosylcimifugin (Yin et al., 2022) have suppressive effect on MAPK pathway, reducing the expression and inflammatory mediators release.

##### 3.3.3.3 Inhibition of the JAK-STAT signaling pathway

The janus kinase/signal transducer and activator of tranions (JAK/STAT) is a common signaling pathway for transducing signals from various cytokines, which widely regulate cell growth, differentiation, inflammation, apoptosis, and other mechanisms. berberine (Zhang et al., 2017), dihydroartemisinin (Jiang et al., 2021), and erianin (Dou et al., 2020) can ameliorate UC’s intestinal mucosal inflammation by downregulating phosphorylated Janus kinase 2 (p-JAK2), JAK2, phosphorylated signal transducer and activator of transcription 3 (p-STAT3), and signal transducer and activator of transcription 3 (STAT3) expression.

##### 3.3.3.4 Inhibition of the PI_3_K/Akt/mTOR signaling pathway

Studies have shown that the PI_3_K/AKT signaling pathway plays an important role in the occurrence of UC (Dong et al., 2022). The inflammatory response can be alleviated by blocking this signal transduction pathway, thus presenting a promising target for treating UC. Interestingly, dihydroartemisinin (Jiang et al., 2021), glutamine (Yan et al., 2020), ihydroartemisinin (Li et al., 2019), luteolin (Vukelic et al., 2020), platycodin D (Guo et al., 2021), quercetin (Zhang et al., 2023), and rhein (Dong et al., 2022) attenuate DSS-induced colitis *via* PI_3_K/AKT signaling pathway inhibition. Additionally, mTOR, a downstream target of PI_3_K/AKT, primarily modulates cell growth and metabolism, promoting anabolism, including ribosome biogenesis and synthesizing nucleotides, proteins, fatty acids, and lids, and inhibiting catabolism. P-mTOR upregulation in the colon tissues of DSS-induced UC rats causes autophagy dysfunction. However, alpinetin (Miao et al., 2019), dioscin (Li et al., 2022), friedelin (Shi et al., 2021), phellodendrine (Su et al., 2021), and rhein (Dong et al., 2022) reverse this effect, return mTOR to normal levels, and inhibit inflammatory cascade, thereby improving intestinal inflammation.

##### 3.3.3.5 Inhibition of the ROCK-MLC signaling pathway

The Ras homologous protein A-Rho kinase (RhoA-ROCK) signaling pathway modulates TJ synthesis, polymerization, and epithelial cell gap permeability, typically linked with the ROCK-MLC pathway (Liu et al., 2020). ROCKs is a serine-threonine kinase family member, including Rho-associated kinase 1 (ROCK1) and ROCK2. ROCK1 directly modulates myosin light chain 2 (MLC2) activation and myosin contraction for TJ depolymerization, accompanied by increased intercellular permeability (Fu et al., 2019). According to a study, UC animals have increased ROCK1 and MLC2 phosphorylation in the colon; however, barbaloin (Gai et al., 2019), geniposide (Xu et al., 2017), and β-patchoulene (Liu et al., 2020) substantially downregulate them to improve the colonic barrier.

##### 3.3.3.6 Inhibition of the NLRP^3^ inflammasome

Recently, it was observed that single nucleotide polymorphisms (SNPs) in genes encoding the NOD-like receptor protein 3 (NLRP3) are associated with IBD susceptibility. NLRP3 belongs to the NOD-like receptor (NLR) family (Kanneganti et al., 2007; Abraham et al., 2017). It is a cytosolic platform protein that assembles inflammasome, a protein complex involved in proteolytic maturation and release of IL-1 and IL-18 pro-inflammatory cytokines (Martinon et al., 2002; Villani et al., 2009). NLRP3 inflammasome regulates various inflammatory and autoimmune disorders (Cao et al., 2020). There were 11 alkaloids [betaine (Chen et al., 2022), berberine (Li et al., 2020), demethyleneberberine (Zhao et al., 2022b), evodiamine (Shen et al., 2019), heterophyllin B (Chen et al., 2022), nigeglanine (Gao et al., 2019), nigakinone (Liu et al., 2023), 8-Oxypalmatine (Cheng et al., 2022), palmatine (Mai et al., 2019), sinomenine hydrochloride (Zhou et al., 2021), and sanguinarine (Li et al., 2022b)], 7 phenylpropanoids [bergenin (Lopes de Oliveira et al., 2019), cinnamaldehyde (Tan et al., 2023), chlorogenic acid (Zeng et al., 2020), ferulic acid (Yu S. et al., 2023), sinapic acid (Qian et al., 2020), schisandrin B (Zhang et al., 2021), and wedelolactone (Wei et al., 2017)], 9 terpenoids [brusatol (Zhou et al., 2018), bryodulcosigenin (Li et al., 2022), ginsenoside Rk3 (Tian et al., 2020), ginsenoside Rg3 (Liu et al., 2023), ginsenoside Rd (Liu C. et al., 2018), geniposide (Pu et al., 2020), mogrol (Liang et al., 2021), picroside II (Yao et al., 2022), and terpinen-4-ol (Zhang et al., 2017a)], 2 flavonoids [naringin (Cao et al., 2018), phloretin (Zhang et al., 2019)], 3 phenols [gallic acid (Yu et al., 2023), hydroxytyrosol (Miao, 2022), and salidroside (Liu et al., 2019)], diacetylrhein (Zohny et al., 2022), dioscin (Cai et al., 2021), 1,25-dihydroxyvitamin D3 (Cao et al., 2020), 3,3′-diselenodipropionic acid (Zheng et al., 2023), physalin B (Zhang et al., 2020), and vitamin D3 (Gao et al., 2023) have been linked with the alleviation of UC *via* NLRP3 inhibition.

##### 3.3.3.7 Activation of the PPARγ signaling pathway

PPARγ is a transcriptional factor expressed mainly in colonic epithelial cells, and UC, its expressions are reduced (Su et al., 1999; Aoyagi et al., 2010). PPARγ activation decreases UC-mediated NF-κB pathway stimulation and inflammatory cytokines (IL-6, IL-1β, and TNF-α) expression (Aoyagi et al., 2010). Furthermore, targeted PPARγ expression alteration enhances mice’s susceptibility towards DSS-induced colitis (Shah et al., 2007; Aoyagi et al., 2010). However, natural compounds, artemisinin (Jia et al., 2022), baicalin (Xu et al., 2021), bergenin (Wang et al., 2018), aesculin (Tiana et al., 2019), convallatoxin (Li et al., 2019), dioscin (Wu et al., 2021), d-pinitol (Lin et al., 2021), demethyleneberberine (Zhao et al., 2022b), emodin (Luo et al., 2022), glycyrrhizin (Sethuraman et al., 2015), geniposide (Zhang et al., 2017d), honokiol (Wang et al., 2022), hyperoside (Cheng et al., 2021), luteolin (Li et al., 2021), naringin (Cao et al., 2018), phloretin (Zhang et al., 2019), salidroside (Liu et al., 2019), stevioside (Mostafa et al., 2020), tetramethylpyrazine (He et al., 2012), and zingerone (Zhang et al., 2022), can upregulate PPARγ expression to alleviate UC.

##### 3.3.3.8 Regulation of the Wnt/β-catenin signaling pathway

It has been observed that the Wnt signaling pathway substantially affects epithelial cell proliferation to repair mechanical barriers (Kuhnert et al., 2004). Wnt modulates β-catenin expression and is involved in the pathological and physiological mechanisms of injury (Whyte et al., 2012). Multiple research indicates that berberine (Dong et al., 2022c) and 6-gingerol (Ajayi et al., 2018) alleviate UC by maintaining intestinal mucosal barrier function and structure and function, regulating the homeostasis of intestinal mucosal immunity *via* the Wnt/β-catenin pathway.

### 3.4 Regulating other mechanisms

#### 3.4.1 Regulating cellular autophagy

One of the cellular self-protection mechanisms is autophagy, which is a self-protective mechanism that maintains homeostasis. It is an evolutionarily conserved mechanism that starts with the generation of a double-membrane autophagosome with cytoplasmic contents (Xie et al., 2020). It is essential for maintaining intestinal homeostasis, modulation of gut ecology, appropriate intestinal immune responses, and microbial protection. It has been suggested that autophagy can substantially suppress cells’ inflammatory reactions (Lin et al., 2019; Larabi et al., 2020). Notably, natural compounds [i.e., alpinetin (Miao et al., 2019), berberine (Xu et al., 2022c), curcumin (Zhang et al., 2019), dioscin (Li et al., 2022), friedelin (Shi et al., 2021), galangin (Xuan et al., 2020), luteolin (Vukelic et al., 2020), palmatine (Mai et al., 2019), procyanidin A1 (Zhang et al., 2022), resveratrol (Pan et al., 2020)], and salidroside (Liu et al., 2023) can improve autophagy and reduce inflammation in the intestinal disorders. Berberine alleviates DSS-induced UC and suppresses the expression and release of lysozyme by stimulating autophagy via adenosine 5‘-monophosphate (AMP)-triggered protein kinase (AMP-activated protein kinase) (AMPK)/mammalian target of rapamycin (mechanistic target of rapamycin kinase) (MTOR)/unlike autophagy activating kinase 1 ULK1 (unc-51 like autophagy activating kinase 1) pathway (Foerster et al., 2022).

#### 3.4.2 Inhibiting ferroptosis

In 2012, ferroptosis was formally stated as an iron-dependent, non-apoptotic cell death manifested by the accumulation of lipid peroxidation products and the depletion of membrane polyunsaturated fatty acid (Dixon et al., 2012). It is characterized by lipid peroxidation, iron accumulation, and increased ROS generation. Iron sagging includes iron deposition, increased lipid peroxidation, reduced GSH, inactivation of glutathione peroxidase 4 (GPX4), and enhanced lipoxygenase (LOX), all of which are linked with UC pathogenesis (Huang et al., 2022). These findings validate that ferroptosis inhibition might be a novel target for treating UC (Wang et al., 2020; Chen et al., 2021; Dong et al., 2021; Tang et al., 2021). β-Caryophyllene is widely found in various plant essential oils, and its flavor and fragrance resembles bicyclic sesquiterpene (Jha et al., 2021). A study revealed that β-caryophyllene acts as an inhibitor of ferroptosis that represses lipid peroxidation and inflammation, thereby alleviating UC (Wu et al., 2022).

#### 3.4.3 Regulating metabolism pathway

The literature suggests that metabolic reprogramming can regulate the activation of macrophages. The metabolic signals furnish energy and polarize macrophages. M1 macrophages substantially depend on glycolytic metabolism, whereas M2 primarily depends on oxidative phosphorylation (Saha et al., 2017). Glucose is converted to pyruvate and lactic acid glycolysis *via* a series of cytoplasmic enzymes. Pyruvate dehydrogenase kinase 1 (PDK1) knockdown is a key modulator enzyme of glucose metabolism, reducing M1 but enhancing M2 macrophage activation (Tan et al., 2015). Glycolysis inhibitor 2-deoxy-D-glucose (2-DG) reduces M1 macrophage activation and pro-inflammatory cytokines secretion (Wang et al., 2018). It has been revealed that tiliroside alleviates UC by restoring the M1/M2 macrophage balance via the HIF-1α/glycolysis pathway (Zhuang et al., 2021).

#### 3.4.4 Inhibiting endoplasmic reticulum stress responses

The endoplasmic reticulum (ER) is an essential cellular organelle with multiple functions to store free calcium and synthesize, mature, and transport various lipids, proteins, sterols, etc. Because of multiple cellular factors, proteins are unable to fold correctly, resulting in the accumulation of newly synthesized unfolded proteins in cells, thereby promoting ER stress (Song et al., 2021). Much research indicates that ER stress is associated with UC progression. Highly secretory cells, such as intestinal paneth and goblet cells, are specifically impressionable to ER stress (Kaser et al., 2010). Inhibition of ER stress responses is thus an important therapeutic rationale for UC. Limonin might be utilized for this purpose as it blocks the PERK-ATF4-CHOP pathway of ER stress (Song et al., 2021). Furthermore, berberine (Shen et al., 2020) and artesunate (Yin et al., 2021) reduce ER stress-related marker proteins (glucose-regulated protein, GRP78, C/EBP-homologous protein, CHOP) to treat UC.

## 4 Analysis of important natural compounds

**FIGURE 6:**
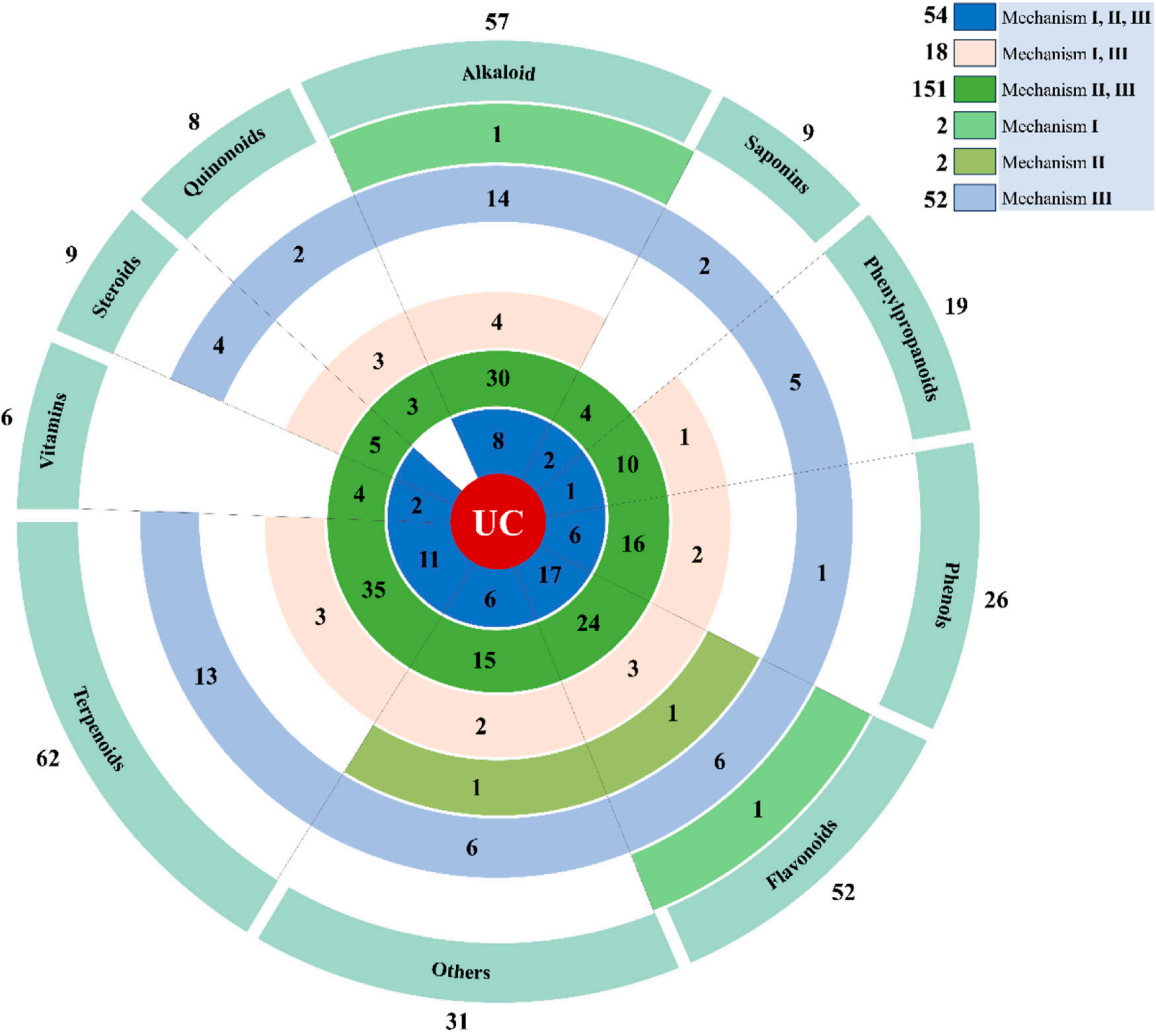
Displays the total amount of natural compounds associated with various mechanisms. The natural compounds involved in this paper are shown in Supplementary Table S1.

Since pharmacotherapy based on a single target has been insufficient for drug development in complex diseases, the emerging multi-target approach is a promising strategy for the search of new drug candidates. Therefore, we analyzed the relationship between the 279 natural compounds and mechanisms covered in this review. The 279 natural compounds, including 62 terpenoids, 57 alkaloids, 52 flavonoids, 26 phenols, 19 phenylpropanoids, 9 steroids, 9 saponins, 8 quinonoids, 6 vitamins, and 31 others, can effectively ameliorate UC. Of these, terpenoids, alkaloids, and flavonoids have the greatest potential for treating UC. It is noteworthy to highlight that a total of 54 compounds are linked to Mechanism Ⅰ, Ⅱ, and Ⅲ; 151 compounds are associated with Mechanism Ⅰ and Ⅱ; 18 compounds are associated with Mechanism Ⅱ and Ⅲ; 4 compounds are associated with Mechanism Ⅰ; 50 compounds are associated with Mechanism Ⅱ; 2 compounds are related to Mechanism Ⅲ (Figure 6).

**FIGURE 7:**
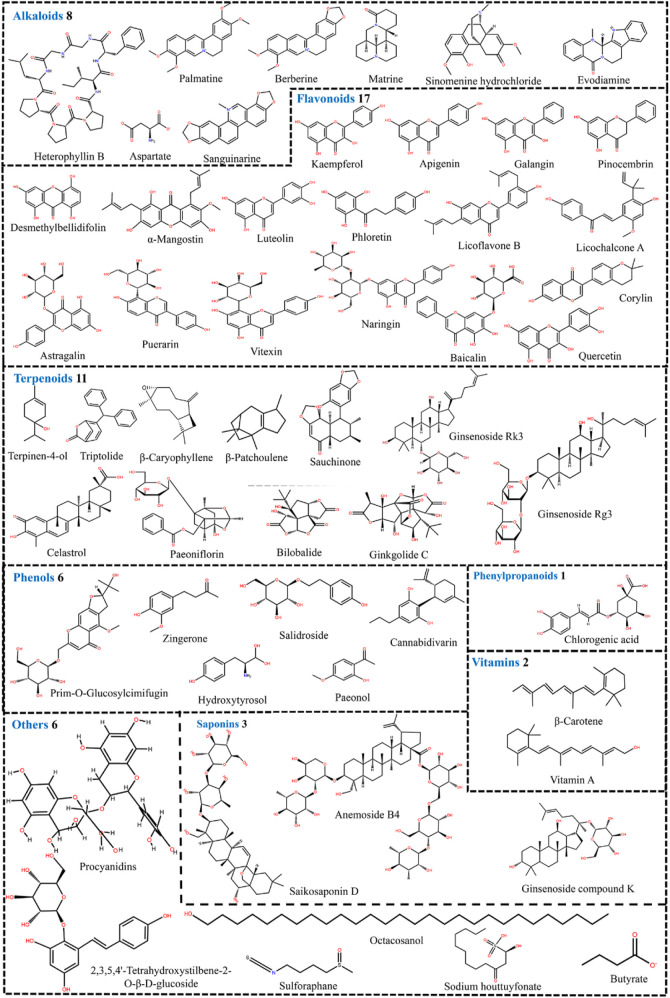
54 natural compounds that can treat ulcerative colitis by regulating multiple mechanisms (Mechanism Ⅰ, Ⅱ, and Ⅲ).

Furthermore, we conducted a comprehensive search of the Pubchem and Drugbank databases to obtain pertinent data regarding the clinical studies associated with the aforementioned natural compounds. Consequently, a total of 6 compounds (andrographolide, berberine, berberine hydrochloride, butyrate, curcumin, and diosmin) for the therapeutic management of UC were identified to be either in the clinical stage of development or already available on the market (Table 1). Interestingly, the vast majority of these compounds can alleviate UC by Mechanism Ⅰ, Ⅱ, and Ⅲ. This indicates that we should pay more attention to the compounds with multiple mechanisms in the follow-up UC drug research (Figure 7).

**TABLE 1:** Natural compounds in clinical trials for the treatment of ulcerative colitis.

Natural compounds	Clinical trial ID/Approval number	Study title	Phase	Drug name
Diosmin	NCT05626166	The efficacy and safety of diosmin in patients with ulcerative colitis	Phase Ⅲ	-
Curcumin	NCT01320436	Curcumin + aminosalicylic acid (5ASA) versus 5ASA alone in the treatment of mild to moderate ulcerative colitis	Phase Ⅲ	-
Butyrate	NCT05218850	The use of butyrate therapy in pediatric ulcerative colitis	Phase Ⅰ	-
Berberine	NCT02962245	Efficacy of treatment with berberine to maintain remission in ulcerative colitis	Phase Ⅳ	-
Berberine hydrochloride	H61022181	-	Approved	Berberine Hydrochloride Tablets
Andrographolide	NCT00659802	Phase II study of HMPL-004 in patients with ulcerative colitis	Phase Ⅱ	-
	NCT01882764	HMPL-004 maintenance treatment in subjects with mild to moderate ulcerative colitis	Phase Ⅲ	-
	NCT01805791	A phase III trial of HMPL-004 in patients with mild to moderate active ulcerative colitis	Phase Ⅲ	-

Note: Drugbank and Pubchem databases were used to obtain natural compounds used in clinical studies.

Many synthetic drugs are currently in use to treat UC such as 5-aminosalicylic acid (5-ASA) (Hossen et al., 2020). Therapeutic mechanisms of 5-ASA for UC include inhibition of cyclooxygenases and lipoxygenase, activation of peroxisome proliferator activated receptor γ, inhibition of T-cell proliferation and activation, reduction of chemotaxis, adhesion and phagocytosis, inhibition of nuclear factor-κβ (Hauso et al., 2015). Overall, 5-ASA appears to exert its therapeutic effect by topical action on the affected areas of inflammation. This is the same as one of the mechanisms (Mechanism III) by which natural compounds treat UC. However, the mechanism of natural compounds against UC is more complex compared to synthetic drugs. Furthermore, 5-ASA have some drawbacks as long-term use results in side effects including nausea, vomiting, fatigue, diarrhea, abdominal pain, pulmonary fibrosis, etc (Rogler, 2010). For centuries, herbal treatments have shown their potential to ameliorate countless diseases and disorders with no or fewer side effects. In conclusion, natural compounds have a richer mechanism for treating UC than synthetic drugs, and natural compounds are more abundantly available and have fewer side effects.

## 5 Concluding remarks and future directions

This review provides a comprehensive overview of the protective effects exhibited by natural substances against UC, while also delving into their probable mechanisms of action in mitigating colitis. Results indicated that 279 natural compounds (62 terpenoids, 57 alkaloids, 52 flavonoids, 26 phenols, 19 phenylpropanoids, 9 steroids, 9 saponins, 8 quinonoids, 6 vitamins, and 31 others) can act on various mechanisms to improve UC, such as regulating gut microbiota and metabolites (Mechanisms I), protecting the intestinal mucosal barrier (Mechanisms II), regulating intestinal mucosal immune response (Mechanisms III), as well as the other mechanisms (cellular autophagy modulation and ferroptosis inhibition). More importantly, (1) 54 natural compounds exhibit their therapeutic effects by modulating Mechanisms I, II, and III, which can be used to develop multitargeted drugs for UC; Terpenoids, alkaloids, and flavonoids have the greatest potential for treating UC. (2) Mechanism III is regulated by all natural compounds; Mechanisms II and III can be modulated by at least half of the compounds, which may give information on the etiology of UC. In conclusion, this review serves as a comprehensive resource for the pharmaceutical industry, researchers, and clinicians seeking novel therapeutic approaches to combat UC. Harnessing the therapeutic potential of these natural compounds may significantly contribute to the improvement of the quality of life of patients with UC and promotion of disease-modifying therapies in the future.

This review fails to resolve some issues and requires further research and refined methodology to provide evidence for the natural compound’s therapeutic efficacy. The limitations include: (1) Disadvantages including reduced water insolubility, oral bioavailability, rapid metabolism, and increased degradation limit the clinical use of various natural compounds. However, different drug delivery strategies can resolve these issues. (2) Clinical trials are required to assess natural compounds' safety and efficacy profiles, the elucidation criteria of which are not uniform for UC, and the treatment mechanism is not thoroughly studied. Research requires standardization and rationalization to improve UC’s therapeutic effect and promote new drug development. (3) Co-treatment of natural compounds and other drugs should be studied for improved treatment. Furthermore, applying targeted preparations would benefit the targeted delivery of natural compounds with an increased curative effect and potential.

## Figures and Tables

**Table:** 

**ACE**	abundance-based coverage estimator
**AMP**	adenosine 5’-monophosphate
**AMPK**	AMP-activated protein kinase
**CAT**	catalase
**CCL-5**	chemokine ligand 5
**CD**	Crohn’s disease
**CDH1**	e-cadherin
**CHOP**	C/EBP-homologous protein
**COX-2**	cyclooxygenase
**Cxcl2**	CXC motif chemokine ligand 2
**ERK-1/2**	extracellular-signal-regulated kinases 1/2
**ER**	endoplasmic reticulum
**FITC-dextran**	fluorescein isothiocyanate dextran
**GPCR**	G-protein-coupled receptors
**GPX4**	glutathione peroxidase 4
**GRP78**	GSH glucose-regulated protein glutathione
**HNF4A**	hepatocyte nuclear factor 4 alpha
**HO-1**	heme oxygenase-1
**IBD**	inflammatory bowel disease
**IκB**	IkappaB
**IEC**	intestinal epithelial cell
**IFN-γ**	interferon-γ
**IL-10**	interleukin-10
**ILC**	innate lymphoid cells
**iNOS**	inducible nitric oxide synthase
**JAK/STAT**	janus kinase/signal transducer and activator of tranions
**JNK**	c-Jun N-terminal kinase
**LOX**	lipoxygenase
**mTOR**	mammalian target of rapamycin
**MAPK**	mitogen-activated protein kinase
**MDA**	malondialdehyde
**MLC2**	myosin light chain 2
**MPO**	myeloperoxidase
**MTOR**	mechanistic target of rapamycin kinase
**Muc1**	mucin 1
**NK cells**	natural killer cells
**NO**	nitric oxide
**NF-κB**	nuclear factor kappa-B
**NLRP3**	NOD-like receptor protein 3
**NLR**	NOD-like receptor
**PAMPs**	pathogen-associated molecular patterns
**PDK1**	Pyruvate dehydrogenase kinase 1
**p-JAK2**	phosphorylated Janus kinase 2
**PPARγ**	peroxisome proliferator-activated receptor γ
**p-STAT3**	phosphorylated signal transducer and activator of transcription 3
**p38**	p38 kinase
**RhoA-ROCK**	Ras homologous protein A-Rho kinase
**ROS**	reactive oxygen species
**ROCK1**	Rho-associated kinase 1
**STAT3**	signal transducer and activator of transcription 3
**SCFAs**	short-chain fatty acids
**SNPs**	single nucleotide polymorphisms
**TA**	Taurohyodeoxycholic acid
**TCM**	traditional Chinese medicine
**TJs**	tight junctions
**Tff3**	trefoil factor 3
**TNF-α**	tumor necrosis factor alpha
**TLR4**	Toll-like receptor 4
**ULK1**	unc-51 like autophagy activating kinase 1
**UC**	Ulcerative colitis
**ZO-1**	zona occludens 1
**2-DG**	2-deoxy-D-glucose
