# Supplementary material for: Harnessing nature’s pharmacy: investigating natural compounds as novel therapeutics for ulcerative colitis
Source: Front Pharmacol. 2024 Aug 14;15:1394124. doi: 10.3389/fphar.2024.1394124 (PMC11349575; doi:10.3389/fphar.2024.1394124)
Supplement: Supplementary file 1 [file Table1.pdf]

Supplementary materials

Table S1 The mechanism of natural compounds in the treatment of ulcerative colitis

Name	Study model	Intestinal mucosal barrier	Intestinal mucosal immune response	Gut microbiota and metabolites	Classification	Reference
β-Patchoulene	DSS-induced UC mice	↑ Occludin, ZO-1<br>↓ ICAM-1, LPS	↓ MPO, TNF-α<br>↓ TLR4/MyD88/NF-κB pathway	↑ *4C0d-2*, *Bacteroidia*, *Bacteroidales*, *Bacteroidetes*, *Clostridia*, *Firmicutes*, *Mollicutes*, *prevotella*, *TM7-3*, *Tenericutes*<br>↓ *Alphaproteobacteria*, *Enterobacteriaceae*, *Escherichia*, *Proteobacteria*, *Gammaproteobacteria*, *Parabacteroides*, *Proteus*	B	[1]
β-Caryophyllene	DSS-induced UC mice; RSL3-induced RAW264.7 cells and BMDMs	↑ GSH<br>↓ MDA, LDH, ROS	↓ IL-1β, IL-6, MPO, TNF-α	↑ Bacilli<br>↓ Enterobacteriaceae, Peptostreptococcaceae, Proteobacteria	B	[2, 3]
β-Carotene	DSS-induced UC rats; DSS-induced UC mice	↑ GSH, Nrf2<br>↓ MMP-9	↓ COX-2, IFN-γ, IL-1β, IL-6, TNF-α<br>↓ MAPK, NF-κB pathway	↑ *Actinobacteria*, *Firmicutes*<br>↓ *Bacteroidetes*, *Proteobacteria*	J	[4, 5]
α-Mangostin	DSS-induced UC mice	↑ CAT, SOD<br>↓ MDA	↓ MPO, NO<br>↓ MAPK, NF-κB pathway	↑ *Enterococcus*,<br>↓ Bacteroidales, Bifidobacteriales, Clostridiales, Erysipelotrichales, Lactobacillales, *Lactobacillus*	A	[6-8]
Zonarol	DSS-induced UC mice; LPS-induced RAW264.7 cells	-	↓ iNOS, IL-1β, IL-6, NO, TNF-α	-	K	[9]

Zingerone	DSS-induced UC mice	↑ GSH, Muc2, Occludin, SOD, ZO-1<br>↓ MDA	↑ PPARγ<br>↓ IFN-γ, IL-1β, IL-12, IL-17A, TNF-α<br>↓ NF-κB pathway	↓ *Escherichia coli*	I	[10]
Zerumbone	DSS-induced UC mice	-	↓ IL-1β, TNF-α	-	K	[11]
Zeaxanthin	Acetic acid-induced UC rats	↑ CAT, GSH, SOD<br>↓ Caspase-3, MDA	↓ COX-2, IFN-γ, IL-1β, IL-6, iNOS, MPO, NF-κB, TNF-α	-	B	[12]
Wogonoside	DSS-induced UC mice;<br>TNF-α induced Caco-2 cells	↑ Claudin-1, Occludin, ZO-1<br>↓ FITC-dextran	-	-	A	[13]
Wogonin	DSS-induced UC mice	↑ GST, GSH, HO-1, SOD<br>↑ Nrf2 pathway<br>↓ TBARS	↑ IL-10<br>↓ COX-2, iNOS, IL-6, MPO, NO, p65, TLR4, TNF-α<br>regulate Nrf2/TLR4/NF-κB pathway	-	A	[14]
Wedelolactone	DSS-induced UC rats；<br>DSS-induced UC mice;<br>LPS+ATP-induced THP-1 cells;<br>TNF-α-induced NCM460 cells	↑ GSH, Occludin<br>↓ Caspase-1	↑ IL-10<br>↓ CCL-5, IFN-γ, IL-1α, IL-1β, IL-2, IL-6, MPO, NF-κB, NLRP3, STAT3, TNF-α<br>↓ IL-6/STAT3, MAPK, NF-κB pathway	-	G	[15, 16]

Vitexin	DSS-induced UC mice	↑ Muc2, Occludin, ZO-1<br>↓ FITC-Dextran	↑ IL-10<br>↓ F4/80, IL-1β, IL-6, TNF-α<br>↓ TLR4/NF-κB pathway	↑ *Alistipes*, *Clostridia*, *Erysipelotrichia*, *Lachnospiraceae_NK4A136_group*, *Lachnospiraceae_UCG-006*, *Verrucomicrobiae*<br>↓ *Bacilli*, *Bacteroides*, *Gammaproteobacteria*, *Helicobacter*	A	[17, 18]
Vitamin E	Acetic acid-induced UC rats；DSS-induced UC rats	↑ GSH, SOD<br>↓ MDA	↓ IFN-γ, IL-1β, IL-6, IL-12, IL-18, MPO, TNF-α	-	J	[19, 20]
Vitamin D3	DSS-induced UC mice; LPS-induced primary intestinal epithelial cells	↓ Caspase-1	↑ IL-10,<br>↓ IL-6, NLRP6, TNF-α	-	J	[21, 22]
Vitamin C	DSS-induced UC mice	↑ CAT, GPX, H2O2, SOD<br>↓ MDA	↓ COX-2, iNOS, IL-1β, IL-6, IL-17, TNF-α<br>↓ NF-κB pathway	-	J	[23, 24]
Vitamin A	DSS-induced UC mice	↑ Muc1, Muc2, Muc4, Occludin	↑ IL-10<br>↓ IL-1β, IL-6, TNF-α	↑ SCFAs<br>↓ *Bacteroides*, *Butyrimimonas*, *Clostridium*, *Clostridium XIVb*, *Escherichia/Shigella*, *Klebsiella*, *Oscillibacter*, *Pseudolavonifractor*, *Parabacteroides*	J	[25]
Vicenin-2	DSS-induced UC mice	↓ MMP-9	↓ COX-2, iNOS, IL-1β, IL-6, MPO, NF-κB p65, TNF-α	-	A	[26]
Ursolic acid	DSS-induced UC mice	↑ SOD<br>↓ MDA	↓ Ccr-2, Csf-1, IL-6, TGF-β, MPO, NF-κB p65<br>↓ IL-6/STAT3, MAPKs, PI3K pathway, regulate immune cells	-	B	[27, 28]

Ursodeoxycholic acid	TNBS-induced UC rats	↓ AP activity	↑ IL-10	-	E	[29]
Tyrosol	DSS-induced UC rats	↑ CAT, GSH, GSH-Px, Nrf2<br>↓ MDA	↓ COX-2, IL-6, NF-κB, TNF-α	-	K	[30]
Tryptophan	DSS-induced UC mice	↑ Muc1, Muc2, Muc3, Muc4, Reg3γ<br>↓ TBARS	↑ Foxp3, IL-22,<br>↓ CCL2, CXCL1, CXCR3, IL-1β, IL-17, IL-6, MPO, TNF-α	-	C	[31, 32]
Triptolide	DSS-induced UC mice；<br>LPS+IFN-γ-induced RAW264.7 cells	↑ Claudin-1, Occludin<br>↓ROS<br>↑ Nrf2/HO-1 pathway	↑ Arg-1, CCL2, M2 macrophages<br>↓ CD80, CXCL10, IL-1β, IL-6, M1 macrophages, MCP-1, TNF-α<br>↓ PDE4B/AKT/NF-κB Axis	↑ *Clostridiales*, *Firmicutes*, *Oscillospira*, *Psychrobacter*, *Ruminococcus*, *Staphylococcus*, *S24-7*<br>↓ *Bacteroides*, *Lachnospiraceae*	B	[33-36]
Tiliroside	DSS-induced UC mice;<br>LPS+IFN-γ-induced e BMDMs;<br>IL-4-induced e BMDMs	-	↑ M2 macrophages<br>↓ IL-1β, iNOS, M1 macrophages, MPO	-	A	[37]
Thymol	Acetic acid-induced UC rats	↓ MDA	↓ COX-2, IL-1β, IL-6, MPO, NF-κB p65, TNF-α<br>↓ NF-κB pathway	-	I	[38, 39]
Theophylline	Acetic acid-induced UC rats	-	↓ IL-1β, IL-6, MPO, TNF-α	-	C	[40]

Tetrandrine	DSS-induced UC mice	-	↓ IL-1β, MPO, NF-kB, TNF-α	-	C	[41]
Tetramethylpyrazine	Oxazolone-induced UC mice	-	↑ PPARγ<br>↓ COX-2, iNOS, MPO, NF-κB p65, TNF-α	-	C	[42]
Terpinen-4-ol	DSS-induced UC mice;<br>LPS-induced RAW 264.7	↑ Occludin, ZO-1<br>↓ Caspase-1	↓ MAPK pathway<br>↓ IL-1β, IL-12, MPO, NLRP3, p65, p-IκB, TNF-α<br>↓ NF-κB pathway	↑ *Lactobacillus*<br>↓ Escherichia coli	B	[43]
Taxifolin	DSS-induced UC mice	-	↑ IL-10<br>↓ IL-1β, IL-6, TNF-α<br>↓ NF-κB pathway	↑ SCFAs	A	[44]
Tauroursodeoxycholate	TNBS-induced UC mice	-	↓ IFN-γ, IL-1β, MPO, TNF-α	-	E	[45]
Taurohyodeoxycholic acid	TNBS-induced UC mice;<br>DSS-induced UC mice;	↑ Bcl-2<br>↓ Caspase-3	↑ Foxp3, IL-4, IL-10, STAT6, TGF-β1<br>↓ CXCL2, IFN-γ, IL-1β, IL-6, IL-12p70, IL-17A, IL-21, IL-22, MPO, STAT3, STAT4, T-bet, TNF-α, Th1/Th2 cells, Th17/Treg cells	-	E	[46-48]

Taurocholate	TNBS-induced UC mice	-	↓ IFN-γ, IL-1β, MPO, TNF-α	-	E	[49]
Taurine	TNBS-induced UC rats	↑ Bcl-2, GSH<br>↓ Bax, MDA	↓ MPO	-	E	[50]
Taraxasterol	DSS-induced UC mice;<br>LPS-induced HT-29 cells	↓ Bax, Caspase-3	↓ IL-6, p53, TNF-α	-	E	[51]
Syringic acid	Acetic acid-induced UC rats;<br>DSS-induced UC mice;<br>LPS-induced RAW 264.7	↑ HO-1, NQO1, Nrf2	↓ CD68, IL-1β, IL-6, MPO, NF-κB p65, p-IκB-α, p-STAT3Y705, TNF-α	-	I	[52, 53]
Sulforaphane	DSS-induced UC mice	↑ Nrf2, ZO-1	↓ IFN-γ, IL-6, MPO, TNF-α	↑ SCFAs<br>↑ *Bacteroides*, *Butyricicoccus*, *Parabacteroides*, *Prevotellaceae*, *Proteobacteria*, *Rikenellacea*<br>↓ *Bacteroidales_S24-7*, *Bacteroidota*, *Campylobacteraceae*, *Erysipelototrichaeae*, *Firmicutes*, *Firmicutes/Bacteroides*, *Parabacteroides*, *Turibaccharacter*, *Verrucomicrobiota*	K	[54, 55]
Stevioside	Acetic acid-induced UC rats;<br>DSS-induced UC mice;<br>LPS-induced RAW 264.7 cells	↑ CAT, GSH, GST, HO-1, Nrf2, SOD<br>↓ ROS	↑ PPARγ<br>↓ COX-2, IL-6, iNOS, MPO, NO, TNF-α<br>↓ NF-κB, MAPK pathway	-	B	[56, 57]
Squalene	DSS-induced UC mice	-	↓ COX-2, iNOS, IL-1β, TNF-α<br>↓ p38 MAPK, NF-κB pathway	-	B	[58]

Sophocarpine	DSS-induced UC mice	-	↓ MPO	-	C	[59]
Sodium houttuyfonate	*Candida albicans*+DSS-induced UC mice*;*<br>TNBS+Ethyl alcohol-induced UC rats	↑ Claudin-1, Occludin, ZO-1<br>↓ LPS, MDA	↑ IL-10<br>↓ IL-1β, IL-6, IL-8, MPO, NF-κB, TLR4, TNF-α	↑ *Lachnospiraceae_NK4A136_group*, *Lactobacillus, norank_f_Muribaculaceae*<br>↓ *Bacteroides, Klebsiella*	K	[60, 61]
Skimmianine	TNBS+Ethyl alcohol-induced UC rats;<br>LPS-induced HT29 cells	↓ LBP	↓ TLR4	-	C	[62]
Sinomenine hydrochloride	DSS-induced UC mice	↓ Caspase-1	↑ Arg-1, IL-10<br>↓ iNOS, IL-6, NLRP3, TNF-α	↑ Bacteroidia<br>↓ *Proteobacteria*, γ-proteobacteria	C	[63]
Sinomenine	DSS-induced UC mice<br>DSS-induced human colonic epithelial cells	↑ HO-1, SOD<br>↑ Nrf2/NQO1 pathway	↓ CCL2, CCL5, iNOS, IL-1β, IL-6, NO, TNF-α<br>↓ NF-κB pathway	-	C	[64, 65]
Sinigrin	DSS-induced UC mice	↑ CAT, GST, GSH, SOD<br>↓ MDA	↓ COX-2, CD68, F4/80, IL-1β, IL-6, IL-17, MCP-1, MPO, TNF-α<br>↓ MAPK pathway	-	C	[66]

Sinapic acid	Acetic acid-induced UC rats;<br>DSS-induced UC mice	↑ Bcl-2, CAT, Claudin-1, GSH, GSH-Px, HO-1, Nrf2, Occludin, SOD, ZO-1<br>↓ Bax, Caspase-1, Caspase-3, MDA	↓ COX-2, iNOS, IL-6, MPO, NF-κB p65, NLRP3, NO, TNF-α	-	G	[67, 68]
Shikonin	DSS-induced UC mice;<br>LPS-induced RAW 264.7 cells	-	↑ IL-10<br>↓ CD86, F4/80, iNOS, IFN-γ, IL-1β, IL-6, TNF-α	-	D	[69]
Sesamin	DSS-induced UC mice;<br>Caco-2 cells	↑ GR, GSH, HO-1, Keap-1, NQO1, Nrf2, SOD<br>↓ MDA	↑ p-AKT/AKT, p-ERK/ERK<br>↓ IL-1β, IL-6, TNF-α	-	G	[70]
Serine	DSS-induced UC mice	↓ Caspase-3	↓ IL-1β, IL-6, MPO, TNF-α	-	C	[71]
Sericic acid	DSS-induced UC mice;<br>LPS-induced RAW264.7 cells	↑ HO-1, SOD<br>↑ Nrf2 pathway<br>↓ MDA	↓ COX-2, iNOS, IL-1β, IL-6, NO, TNF-α<br>↓ NF-κB pathway	-	B	[72]
Schisandrin B	DSS-induced UC mice;<br>Caco-2 cells;<br>ATP+LPS-induced intestinal epithelial Cells;<br>HCT-116 cells	↑ E-cadherin, Occludin<br>↑ AMPK/Nrf2 pathway<br>↓ Caspase-1, ROS, GSDMD	↓ IL-1β, IL-6, IL-18, NLRP3, p-Erk, p-JNK, p-p38/MAPK, TNF-α	-	G	[73, 74]
Sauchinone	DSS-induced UC mice	↑ Claudin-1, NQO1, Occludin, ZO-1	↓ IL-1β, IL-6, TNF-α<br>regulate NQO1/NF-κB pathway	↑ *Firmicutes*, *Oscillospira*, *Ruminococcus*<br>↓ *Bacteroidetes*, *Bacteroides*, *Helicobacter*, *Proteobacteria*, *Verrucomicrobia*	B	[75]

Sarsasapogenin	TNBS-induced UC rats	↑ GSH, SOD<br>↓ MDA	↓ MPO, NO	-	H	[76]
Sanguinarine	DSS-induced UC mice;<br>Acetic acid-induced UC mice;<br>LPS+Nigericin-induced THP-1 cells	↓ Caspase-1, ROS	↑ IL-4, IL-10<br>↓ IFN-γ, IL-1β, IL-6, IL-13, IL-18, MPO, NLRP3, NF-κB p65, TNF-α<br>↓ NLRP3/IL-1β pathway	↑ *Muribaculaceae_unclassified*, *Mucispirillum*, *Rumini-clostridium_5*<br>↓ *Escherichia-Shigella*, *Lachno-spiraceae_NK4A136_group*, *Helicobacter*, *Eisenbergiella*	C	[77, 78]
Salidroside	DSS-induced UC mice;<br>LPS+ATP-induced BMDMs	↓ Caspase-1	↑ IL-10, PPARγ, Treg cells<br>↓ IFN-γ, IL-1β, IL-6, IL-17A, MAPK p38, NLRP3, p62, p65, Th17 cells, TNF-α<br>↓ TREM1 pathway	↑ Deinococcus-Thermus, Firmicutes, Gemmatimonadetes, Nitrospirae, uncultured_bacterium_f_Lachnospiraceae<br>↓ Akkermansia, Bateroidetes, Lachnospiraceae_NK4A136_group, uncultured_bacterium_g_Lachnospiraceae_NK4A136_group, uncultured_bacterium_f_Muribaculaceae	I	[79, 80]
Saikosaponin D	DSS-induced UC mice	↑ Claudin-1, Muc1, Muc2, ZO-1	↑ IL-10<br>↓ IL-1β, IL-6, TNF-α	↑ Shannon index<br>↑ *Anaerotruncus*, *Akkermansia*, *Blautia*, *Mucispirillum*, *Oscillibacter*, *Ruminiclostridium_5*, *Ruminiclostridium_9*, *Ruminiclostridium*, *unclassified Lachnospiraceae*<br>↓ Simpson index	H	[81]
Saikosaponin A	DSS-induced UC mice	-	↓ IL-1β, MPO, TNF-α<br>↓ NF-κB pathway	-	H	[82]

Rosmarinic acid	TNBS-induced UC rats;<br>DSS-induced UC mice	↑ GSH, Muc2, SOD, ZO-1<br>↓ MDA	↑ IL-10, Treg cells<br>↓ CD4+ T cells, CD8+ T cells, COX-2, iNOS, IFN-γ, IL-1β, IL-6, IL-22, MPO, NF-κB p65, p-STAT3, TNF-α<br>↓ NF-κB pathway	-	G	[83, 84]
Riboflavin	Acetic acid-induced UC rats	↑ GSH	↑ TGF-β1<br>↓ MPO	-	J	[85]
Rhein	DSS-induced UC mice;<br>LPS-induced RAW264.7 cells	-	↓ IL-1β, IL-6, MPO, TNF-α<br>↓ PI3K/Akt/mTOR pathway	↑ Bacteroidetes, *Rikenellaceae*, *Unspecified-S24-7*<br>↓ *Enterobacteriaceae*, Proteobacteria, *Turicibacter*	D	[86, 87]
Resveratrol	DSS-induced UC mice;<br>TNBS-induced UC mice;<br>TNBS-induced UC rats;<br>Oxazolone-induced UC rats;<br>HCT 116 cell	↑ GSH-Px, Occludin, SOD, ZO-1<br>↓ ICAM-1, MDA	↑ IL-10, TGF-β1<br>↓ Atg12, COX-2, iNOS, IFN-γ, IL-1β, IL-4, IL-6, IL-8, IL-17, MAPK, MPO, PGES-1, p38 MAPK, p53, TNF-α<br>↓ HIF-1α-Th17, PI3K/Akt/VEGFA, Wnt pathway	-	I	[88-97]
Quercitrin	DSS-induced UC rats	-	↓ MPO, TNF-α	-	A	[98]
Quercetin	DSS-induced UC mice;<br>Ochratoxin A-induced UC rats	↑ GSH, SOD<br>↓ MDA	↓ IL-1β, IL-6, MPO, NO, TNF-α<br>↓ PI3K/AKT pathway	↑ *Bacteroidaceae*, *Erysipelotrichia*, *Oscillospirales*, *Ruminococcaceae*	A	[99-101]

Puerarin	TNBS-induced UC rats;<br>DSS-induced UC mice	↑ CAT, Claudin-1, Gobelt cells, GSH, HO-1, Muc2, NQO1, Nrf2, Occludin, SOD, ZO-1<br>↓ MDA	↓ COX-2, iNOS, IFN-γ, IL-1β, IL-6, MPO, NO, TNF-α<br>↓ NF-κB pathway	↑ SCFAs<br>↓ *Ruminococcus 1*, *Ruminococcaceae UCG-009*	A	[102, 103]
Procyanidin A1	DSS-induced UC mice;<br>LPS-induced HT-29 and IEC-6 cells	↑ AMP/ATP	↑ AMPK/mTOR pathway<br>↓ IL-1β, IL-6, TNF-α	-	I	[104]
Prim-O-Glucosylcimifugin	DSS-induced UC mice;<br>LPS-induced RAW 264.7 cells	↑ Claudin-3, Occludin, ZO-1	↓ COX-2, iNOS, IL-1β, IL-6, TNF-α<br>↓ AKT, MAPK, NF-κB pathway	↑ *Bacteroidetes*, *Firmicute*, *Lactobacillus*, *Proteobacteria*<br>↓ Enterobacteriales, Gammaproteobacteria, Helicobacteraceae	I	[105]
Polydatin	DNBS-induced UC mice;<br>DSS-induced UC mice	↑ Bcl-2, GSH-Px, SOD<br>↓ Bax, Caspase-3, ICAM-1, MDA	↓ IL-1β, IL-6, MPO, NF-κB p65, TNF-α<br>↓ NF-κB pathway	-	I	[106-108]
Plumericin	DNBS-induced UC mice;<br>LPS + IFN-induced IEC-6 cells	↑ Bcl-2, Bcl-xL, Claudin-1, E-cadherin, Occludin<br>↓ Bax, Caspase-3, ICAM-1	↓ MPO	-	B	[109]
Platycodin D	DSS-induced UC mice;<br>LPS-induced RAW 264.7 cells	↑ Ocln, TJP1<br>↓ FITC-Dextran	↑ Arg-1, IL-10, M2 macrophages<br>↑ PI3K/Akt pathway<br>↓ CD86, iNOS, IL-1β, IL-6, M1 macrophages, TNF-α<br>↓ NF-κB pathway	-	H	[110]
Piperine	TNBS-induced UC rats;<br>Acetic acid-induced UC mice	↑ Claudin-1, GSH, Occludin, SOD, ZO-1<br>↓ Caspase-1, MDA	↑ IL-10<br>↓ COX-2, iNOS, IFN-γ, IL-1β, IL-6, MPO, NF-κB p65, NO,	-	C	[111, 112]

			TNF-α, regulate IkB-α/NF-kB pathway			
Pinocembrin	DSS-induced UC rats;<br>DSS-induced UC mice;<br>LPS-induced RAW 264.7 cells;<br>LPS-induced Caco-2 cells	↑ Claudin-1, JAM-A, Occludin, ZO-1	↑ TGF-β<br>↓ IFN-γ, iNOS, IL-1β, IL-6, IL-15, MyD88, TNF-α<br>↓ TLR4/MD2/NF-κB pathway	↑ Shannon index, *Alloprevotella* spp., *Bacteroidetes*, *Desulfovibrio* spp, *Firmicutes*, *Lactobacillus* spp, *Lachnospiraceae*, SCFAs<br>↓ *Enterobacteriaceae*, *Enterococcus*, *Escherichia-Shigella*, *Protebacteria*	A	[113, 114]
Picroside II	DSS-induced UC mice;<br>LPS+ATP-induced THP-1 cells	↓ Caspase-1	↓ IL-1β, IL-6, TNF-α, p-p65/p65,<br>NLRP3<br>↓ NF-κB pathway	-	B	[115]
Phytic acid	DSS-induced UC mice;<br>LPS-induced Caco-2 cells	↑ Cludin-3, Occludin, ZO-1	↓ COX2, iNOS, IL-Iβ, IL-6, MPO, TNF-α<br>↓ AKT/NF-κB pathway	-	K	[116]
Physalin B	DSS-induced UC mice;<br>LPS-induced RAW 264.7 cells	-	↓ IL-1β, IL-6, MPO, NLRP3, TNF-α<br>↓ NF-κB, STAT3 pathway	-	E	[117]
Phloretin	DSS-induced UC mice;<br>Acetic acid-induced UC rats;<br>LPS-induced RAW 264.7 cells	↑ Claudin-1, GSH, Muc2, Occludin, SOD, ZO-1<br>↓ Caspase-1, MDA	↑ IL-10, PPARγ<br>↓ iNOS, IFN-γ, IL-1β, IL-6, IL-12, IL-17A, IκB, MPO, NO, NLRP3, TNF-α, TLR4<br>↓ NF-κB pathway	↑ *Alistipes*, *Akkermansia*, *Bacteroidetes*, *Lactobacillus*<br>↓ *Acetatifactor*, *Butyricicoccus*, *Escherichia coli*, *Firmicutes*, *Oscillibacter*, *Ruminiclostridium_6*, *Ruminiclostridium_9*, *Tyzzerella_3*, *Tyzzerella*	A	[118-120]
Phillygenin	DSS-induced UC mice;<br>LPS-induced RAW264.7 Cells	↑ E-cadherin, Gobelt cells, Occludin, SOD, ZO-1<br>↓ MDA	↓ IL-1β, IL-6, MPO, TLR4, TNF-α<br>↓ MAPK, NF-κB Pathway	-	G	[121]

Phellodendrine	DSS-induced UC mice<br>H2O2-induced Caco-2 cells	-	↑ p-AMPK/AMPK<br>↓ p-mTOR/mTOR<br>regulate AMPK/mTOR pathway	-	C	[122]
Perillyl alcohol	DSS+ Restraint stress -induced UC mice	↑ GSH<br>↓ MDA	↓ IL-1β, MPO, NO, TLR-4, TNF-α<br>↓ TLR4/NF-κB, IL-6/JAK2/STAT3 pathway	-	B	[123]
Pedunculoside	DSS-induced UC mice;<br>LPS-induced RAW 264.7 cells and primary peritoneal macrophages	-	↓ COX-2, iNOS, IL-1β, IL-6, MPO, P65, TNF-α<br>↓ MAPK, AKT/NF-κB pathway	-	B	[124]
Patchouli alcohol	DSS-induced UC mice;<br>TNBS-induced UC rats	↑ Bcl-2, Claudin-1, Muc1,, Occludin, ZO-1, ZO-2<br>↓ Bax	↓ COX-2, iNOS, IFN-γ, IL-1β, IL-4, IL-6, MPO, TNF-α<br>↓ NF-κB parthway	-	B	[125, 126]
Parthenolide	DSS-induced UC mice	-	↓ IL-1β, IκBα, MPO, p-NF-κB p65, TNF-α<br>↓ NF-κB pathway	-	B	[127]
Panaxynol	DSS-induced UC mice;<br>IFNγ-stimulated RAW264.7 cells;<br>IFNγ-stimulated ANA-1 cells	-	↓ CD11b, COX-2	-	K	[128]
Palmitoylethanolamide	DSS-induced UC mice;<br>DNBS-induced UC rats	↑ HO-1, Nrf2<br>↓ HIF-1α, ICAM-1, MDA, PARP	↓ IL-1β, MPO, TNF-α, NF-κB<br>↓ Akt/mTOR/P70s6k pathway	-	C	[106, 129]

Palmatine	DSS-induced UC mice;<br>LPS+ATP induced THP-Ms	↑ Bcl-2, Claudin-1, Muc1, Muc2, ZO-1, ZO-2<br>↓ Bax, Caspase-1	↑ IL-10<br>↓ F4/80 +cells, IFN-γ, IL-1β, IL-4, IL-6, MPO, NLRP3, TNF-α	↓ Proteobacteria	C	[130, 131]
Paeonol	DSS-induced UC mice	↑ Occludin, ZO-1	↑ IL-4, IL-10<br>↓ IL-1β, IL-6, IL-8, NF-κB, TNF-α, TLR-2, TLR-4	↑ Bile acid, SCFAs, Bacteroides spp., *Lactobacillus*, Patescibacteria<br>↓ Bacteroides, Escherichia-Shigella, *Romboutsia*, Turicibacter	I	[132, 133]
Paeoniflorin	DSS-induced UC mice;<br>TNBS-induced UC mice;<br>LPS-induced RAW264.7 cells;<br>LPS-treated DCS	↑ Bcl-2<br>↓ Bax, Caspase-3, Caspase-9	↑ Foxp3, IL-10, Treg cells<br>↓ COX-2, CCL11, CCL24, CCR3, Eosinophil cells, iNOS, IFN-γ, IL-1β, IL-2, IL-4, IL-5, IL-6, IL-12, IL-17, MCP-1, MPO, MyD88, NF-κB p65, Th17/Treg, TNF-α<br>↓ MAPK/NF-κB, NF-κB, NF-κB pathway	↓ *Clostridium*, Firmicutes	B	[134-138]
Octacosanol	DSS-induced UC mice	↑ Occludin, ZO-1	↓ IL-6, TNF-α	↑ SCFAs<br>↑ *Prevotellaceae*, *S24- 7*, *Turicibacter*<br>↓ *Enterococcus*, *Stenotrophomonas*	K	[139]
Norisoboldine	DSS-induced UC mice	-	↑ Foxp3, IL-10, TGF-β, Treg cells<br>↓ IFN-γ, IL-1β, IL-6, IL-17, IL-17A, MPO, TNF-α, Th17 cells	-	C	[140]

Nigeglanine	DSS-induced UC mice	↑ Occludin, ZO-1<br>↓ Caspase-1	↑ IL-10<br>↓ IL-1β, IL-6, IL-12, MPO, NLRP3, TNF-α<br>↓ MAPK, NF-κB pathway	-	C	[141]
Nigakinone	DSS-induced UC mice;<br>DSS-induced UC rats	↑ Claudin-1, Occludin, ZO-1<br>↓ Caspase-1	↓ iNOS, IL-1β, MPO, NLRP3, TNF-α	-	C	[142, 143]
Nicotine	DSS-induced UC mice;<br>IL-6-induced Caco-2 cells	-	↓ IL-6, MPO, STAT3, TNF-α	↑ *Clostridium*, *Porphyromonas*	C	[144]<br>[145, 146]
Niacin	DSS-induced UC mice;<br>Peritoneal macrophage treated with niacin	↓ Apoptotic epithelial cells	↓ MPO, TNF-α<br>regulate immune cells	-	C	[147, 148]
Nervonic acid	DSS-induced UC mice;<br>LPS-induced RAW264.7 cells	↑ Bcl-2<br>↓ Bax	↑ IL-10<br>↓ COX-2, iNOS, IL-1β, IL-6, MPO, NO, TLR4, TNF-α<br>↓ MAPK, NF-κB pathway	-	K	[149]
Nerolidol	Acetic acid-induced UC rats	↑ CAT, GSH, SOD<br>↓ MDA	↓ IL-1β, IL-6, IL-23, MPO, TNF-α	-	K	[150]
Neferine	DSS-induced UC mice;<br>LPS/LPS+Z-VAD-induced RAW 264.7 cells	↓ ICAM-1	↓ COX-2, iNOS, IL-6, MPO, NO, TNF-α	-	C	[151, 152]

Naringin	DSS-induced UC mice;<br>TNBS-induced UC mice;<br>TNBS-induced UC rats;<br>LPS-induced IEC-6 and RAW 264.7 cells	↑ CAT, GSH, Occludin, SOD, ZO-1<br>↓ Caspase-1, Caspase3, MDA	↑ PPARγ<br>↓ COX2, iNOS, IFN-γ, IL-1β, IL-6, IL-12, MPO, NLRP3, TNF-α<br>↓ MAPK, NF-κB pathway	↑ *Firmicutes/Bacteroides Firmicutes*<br>↓ *Bacteroidota*, *Proteobacteria*	A	[153-156]
Naringenin	DSS-induced UC mice;<br>Acetic acid-induced UC rats;<br>LPS-inducedd RAW264.7 cells	↑ CAT, SOD, T-GSH<br>↓ ICAM-1, TBARS	↓ COX-2, iNOS, IL-1β, IL-6, MCP-1, NF-kB, NO, TLR4, TNF-α<br>↓ TLR4/NF-kB pathway	-	A	[157, 158]
Myristicin	Acetic acid-induced UC rats	↑ HO-1, Nrf2	↓ COX-2, MPO, NF-κB	-	G	[159]
Myricetin	DSS-induced UC mice	↑ Claudin-1, GSH-Px, Occludin, SOD<br>↓ MDA	↑ Treg cells<br>↓ IL-1β, IL-6, MPO, NO, Th1 cells, Th17 cells	-	A	[160, 161]
Morroniside	DSS-induced UC mice	↑ Claudin-3, E-Cadherin, Muc2, Occludin, ZO-1	↓ IL-1β, IL-6, TNF-α<br>↓ STAT3/NF-κB pathway	-	B	[162]
Mollugin	DSS-induced UC mice	-	↓ TNF-α	-	D	[163]
Mogrol	DSS-induced UC mice;<br>TNF-α-induced NCM460;<br>LPS-induced THP-M	↑ Occludin, ZO-1	↑ IL-10, IκB-α<br>↓IL-1β, IL-17, NLRP3, SIRT1	-	B	[164]

Mimulone	DSS-induced UC rats	-	↑ MMP2<br>↓ COX-2	-	A	[165]
Methyl gallate	DSS-induced UC mice;<br>LPS-inducedd RAW264.7 cells	-	↑ Arg-1, IL-10, IL-4, M2 macrophages,<br>↓ IL-1β, IL-6, M1 macrophages, NO, TNF-α<br>↓ TLR4/NF-κB pawthway	↑ Cyanobacteria, *Muribaculum*, *unclassified_f_Lachnospiraceae*<br>↓ *Faecalibaculu, Turicibacter*	I	[166]
Menthol	Acetic acid-induced UC rats	-	↓ IL-1β, IL-6, MPO, TNF-α	-	B	[167]
Melittin	Acetic acid-induced UC mice	↑ GSH, SOD<br>↓ MDA	↓ COX-2, IL-6, p38 MAPK, TNF-α, TLR4, TRAF6<br>↓ NF-κB, p38MAPK pathway	-	C	[168]
Matrine	DSS-induced UC mice;<br>TNBS+Ethyl alcohol-induced UC rats	↑ Claudin-1, Claudin-2, Occludin, Tff3, ZO-1	↓ IL-1β, IL-6, IL-8, TNF-α<br>↓ PPARα pathway	↑ *Barnesiella intestinihominis*<br>↓ Helicobacter ganmani	C	[61, 169, 170]
Lycopene	Acetic acid-induced UC rats;<br>Ochratoxin A-induced UC rats	↑ CAT, GSH, GSH-Px, Nrf-2, SOD<br>↓ MDA	↓ COX-2, IL-1β, IL-6, MPO, NF-κB p65, NO, TNF-α<br>↓ TLR4/TRIF/NF-κB pathway	-	B	[101, 171, 172]
Luteolin	DSS-induced UC mice;<br>DSS-induced UC rats	↑ Occludin, ZO-1<br>↓ Caspase-3, Caspase-9, FITC-Dextran, PARP	↑ ERK1/2, IL-22, PPARγ<br>↓ COX-2, IFN-γ, IL-17, IL-17A, IL-23, TNF-α<br>↓JNK1/2, p38, NF-κB, STAT3 pathway	↑ Bacteroidetes, *Bacteroides, Lachnospiraceae_NK4A136_group*<br>↓ *Lactobacillus, Prevotella_9*	A	[173-175]

Loganin	DSS-induced UC mice	↑ Claudin-3, E-cadherin, Muc2, Occludin, ZO-1	↓ COX-2, CXCL10, IL-1β, IL-6, M1 macrophages, MCP-1, MPO, NF-κB-p65, TNF-α<br>↓ STAT3/NF-κB pathway<br>regulate Sirt1/NF-κB pathway	-	B	[162, 176]
Liriodendrin	DSS-induced UC mice;<br>LPS-induced RAW 264.7 cells	↑ GPX-Px, SOD<br>↓ MDA	↓ IL-1β, IL-6, MPO, TNF-α<br>↓ Akt, NF-κB pathway	-	K	[177]
Limonin	DSS-induced UC mice<br>LPS-induced RAW 264.7 cells	-	↑ IL-10<br>↓ COX-2, iNOS, IL-1β, IL-6, miR-214, MPO, NF-κB p65, pSTAT3/STAT3, TNF-α,<br>regulate STAT3/miR-214 pathway	-	B	[178, 179]
Licoflavone B	DSS-induced UC mice	↑ Claudin-1, Occludin, ZO-1	↑ IL-10<br>↓ IL-1β, IL-4, IL-6, TNF-α<br>↓ MAPK pathway	↑ *Adlercreutzia*, *Bacteroides*, *Faecalibaculum*<br>↓ *Alloprevotella*	A	[180]
Licochalcone A	DSS-induced UC mice	↑ Claudin-1, GSH, Occludin, SOD, ZO-1<br>↑ Nrf2 pathway	↑ IL-10<br>↓ IL-1β, IL-6, MPO, NO, TNF-α<br>↓ MAPK, NF-κB pathway	↑ *Akkermansiaceae*, *Bacteroidaceae*, *Bifidobacteriales*, *Moraxellaceae*<br>↓ *Bacillaceae*, *Defluviitaleaceae*, *Lachnospiraceae*, *Prevotellaceae*	A	[181, 182]
Leonurine	DSS-induced UC mice	-	↓ NF-κB pathway	↑ *Ackermania*, *Parasutterella*<br>↓ *Bifidobacterium*, *Escherichia coli-Shigella*, *Helicobacter*, *Turicibacter*	C	[183]

L-arginine	DSS-induced UC mice	-	↓ IL-1α, IL-1β, IL-6, IL-17, MCP-1, MIP-1α, MIP-1β, MPO	-	C	[184]
Kynurenine	TNBS+Ethyl alcohol-induced UC mice	-	↑ Foxp3, IL-10	-	C	[185]
Kushenin	TNBS-induced UC rats	-	↓ IL-6, NF-κB p65, NOD2	-	I	[186]
Kolaviron	DSS-induced UC rats	↓ H_2O_2, LPO	↓ IL-1β, MPO, NO, TNF-α	-	A	[187]
Kaempferol	DSS-induced UC mice	↑ Claudin-1, Occludin, Tff3, ZO-1<br>↓ Epithelial permeability	↓ COX-2, iNOS, IL-1β, IL-6, MPO, TNF-α<br>↓ LPS-TLR4-NF-κB pathway	↑ Shannon, Simpson, Chao1, index, *Firmicutes/Bacteroidetes*<br>↓ *Enterobacteriales*, *Enterobacteriaceae*, *Escherichia_Shigella species*, *Gammaproteobacteria*, *Proteobacteria*	A	[188, 189]
Juglone	DSS-induced UC mice	-	↑ Foxp3, IL-10<br>↓ IL-1β, IL-6, STAT3, TNF-α	↑ Shannon, Simpson index<br>↑ *Actinobacteriota, Akkermansia, Blautia,* Firmicutes/Bacteroidota, *Bifidobacterium, Lactobacillus*<br>↓ *Bacteroides*, *Desulfovibrio*, *Escherichia-Shigella*, *Proteobacteria, Parasutterella, Turicibacter*, *Verrucomicrobiota*	D	[190]
Jatrorrhizine	DSS-induced UC mice	-	↑ IL-10, TGF-β<br>↓ COX-2, MPO, NOS2, TNF-α<br>↓ TLR4/MyD88/NF-κB pathway	↑ Chao, Shannon index<br>↑ *Akkermansia*<br>↓ Deferribacteres, Proteobacteria, *Desulfovibrio*, *Escherichia-Shigella*, *Mucispirillum*, *Ruminiclostridium_9*, *Rikenella*	C	[191, 192]

Isovitexin	DSS-induced UC mice;<br>LPS-induced RAW264.7 cells	↑ CAT, GSH-Px, SOD	↑ IL-10<br>↓ IL-1β, IL-6, IL-17A, NO, TNF-α<br>↓ MAPK/NF-κB pathway	-	A	[193]
Isobavachalcone	DSS-induced UC mice;<br>LPS-induced RAW264.7 cells	-	↓ COX-2, iNOS, IL-1β, IL-6, MPO, NF-κB p65, NO, TLR4, TNF-α	-	A	[194]
Isatin	DSS-induced UC mice;<br>TNBS+Ethyl alcohol-induced UC rats	↑ Bcl-2, GSH, SOD<br>↓ Bax, Caspase-3, GSH-Px, GSH-Rd, MDA	↑ IL-10, Foxp3<br>↓ COX-2, iNOS, IFN-γ, IL-6, MPO, NO, TNF-α<br>↓ NF-κB, MAPK pathway	-	C	[195, 196]
Irisin	DSS-induced UC mice	-	-	↓ *Erysipelotrichia, Bacteroides-Unclassified, Ruminococcaceae-UCG-014*	A	[197]
Indole-3-Carbinol	DSS-induced UC mice	-	↓ IL-1β, MPO, NF-κB p65, TNF-α	-	C	[198]
Indirubin	DSS-induced UC mice	↑ Bcl-2, GSH, SOD<br>↓ Bax, Caspase-3, MDA	↑ Foxp3, IL-4, IL-10, Treg cells<br>↓ CD4+ T cells, COX-2, iNOS, IFN-γ, IL-2, IL-6, MPO, NO, TNF-α<br>↓ MAPK, NF-κB pathway	-	C	[195, 199]

Imperatorin	TNBS-induced UC rats	↓ ARE, HO-1, ROS<br>regulate Nrf-2/ARE/HO-1 pathway	↓ IL-6, TNF-α	-	G	[200]
Icariin	DSS-induced UC mice	-	↓ NF-κB pathway	↑ *Akkermansia*, *Lactobacillus*, *Lachnospiraceae*<br>↓ *Bacteroides, Helicobacteraceae, Turicibacter*	A	[201]
Hyperoside	DSS-induced UC mice	↑ Claudin-5, Muc2, Occludin, TJP1, ZO-1	↑ FOXP3, IL-10, PPARγ, TGF-β, Treg cells<br>↓ IL-1β, IL-6, IL-17, IL-22, IL-23, TNF-α, Th17 cells	-	A	[202]
Hypaconitine	TNBS+Ethyl alcohol-induced UC rats;<br>LPS-induced HT29 cells	↓ LBP	↓ PGE2<br>↓ TLR4/NF-κB pathway	-	C	[62]
Hydroxytyrosol	Acetic acid-induced UC rats;<br>DSS-induced UC mice	↑ Bcl2, CAT, GPX, GSH-Px, SOD<br>↓ Bax, Caspase-1, MDA, ROS	↑ IL-10<br>↓ COX-2, iNOS, IL-1β, IL-18, MPO, MCP-1, NF-κB, NLRP3, NO, TGF-β, TNF-α	↑ ACE, Chao, Shannon index<br>↑ Actinobacteria, Firmicutes, *Lactobacillus*, Lachnospiraceae NK4A136 group, [*Ruminococcus*] *torques* group, *Roseburia*, SCFAs<br>↓ Simpson index, *Desulfovibrio*, Epsilonbacteraeota, *Helicobacter*, Proteobacteria, *Staphylococcus, Streptococcus*	I	[203, 204]
Hydroxysafflor yellow A	DSS-induced UC rats;<br>LPS-induced RAW264.7 cells	-	↓ IL-1β, IL-6, MPO, TNF-α<br>↓ TLR4/NF-κB pathway	-	A	[205]
Honokiol	DSS-induced UC mice;<br>LPS-induced RAW264.7 cells	↑ Claudin-1, Occludin, ZO-1	↑ PPAR-α, PPARγ<br>↓ COX2, iNOS, IFN-γ, IL-1β, IL-6, NF-κB p65, TNF-α<br>↓ TLR4-NF-κB pathway	-	G	[206, 207]

Homoharringtonine	DSS-induced UC mice;<br>LPS-induced RAW264.7 cells	-	↓ CCL2, IL-1β, IL-6, M1 macrophages polarization, NOS2, TNF-α<br>↓ NF-κB pathway	-	C	[208]
Heterophyllin B	DSS-induced UC mice;<br>TNF-α-induced NCM460 cells	↑ Muc2, Occludin, ZO-1	↑ IL-10,<br>↓ IL-1β, IL-6, IL-17, NLRP3<br>↓ NF-κB/NLRP3 pathway	↑ *Akkermansia muciniphila*, *Akkermansia*, *Blautia*, *Bacteroidetes*, *Bilophila*, *Dubosiella*, *Epsilonbacteraeota*, *Firmicutes*, *Micromonospora*, *Muribaculaceae*, *Proteobacteria*, *Verrucomicrobia*<br>↓ *Escherichia-Shigellaabundance*, *Helicobacter*	C	[209]
Hesperidin methyl chalcone	Acetic acid-induced UC mice	↑ GSH	↑ Total p65/p-p65<br>↓ IL-1β, IL-6, IL-33, TNF-α	-	A	[210]
Hesperidin	DSS-induced UC mice;<br>DSS-induced UC rats;<br>TNBS+Ethyl alcohol-induced UC rats;<br>LPS-induced RAW264.7 cells	↑ Occludin, SOD, ZO-1<br>↓ Bax, Caspase-3, HMGB1 MDA	↑ IL-10<br>↓ IL-1β, IL-6, IL-18, MPO, NO, TNF-α	-	A	[211-214]
Glycyrrhizin	Acetic acid-induced UC rats;<br>DSS-induced UC rats	↑ Catalase, GSH-Px, SOD	↑ PPARγ<br>↓ MPO, TNF-α	-	B	[215, 216]
Glycyrrhetic acid	DSS-induced UC mice	-	↓ COX-2, NF-κB, p-IκBα, NF-κB p65	-	B	[217]
Glutamine	TNBS-induced UC rats;<br>DSS-induced UC mice;<br>Acetic acid-induced UC mice	↑ GSH, SOD<br>↓ Caspase-3, MDA	↓ MPO, NF-κB<br>↓ PI3K/Akt pathway	-	C	[218-220]

Glutamate	TNBS-induced UC rats	↑ Bcl-2, SOD<br>↓ Bax, Caspase-3, MDA	↓ IL-1β, TNF-α	-	K	[221]
Glabridin	DSS-induced UC rats	↑ AMP	↓ iNOS, MPO, NO, TNF-α	-	A	[222]
Ginsenoside Rk3	DSS-induced UC mice	↑Claudin-1, Occludin, ZO-1<br>↓ Caspase-1	↓ iNOS, IL-1β, IL-6, MPO, NLRP3, TNF-α	↑ SCFAs	B	[223]
Ginsenoside Rh2	DSS-induced UC mice;<br>IL-6-induced NCM460 cells	-	↑ TGF-β<br>↓ F4/80, IL-1β, IFN-γ, IL-6, TNF-α<br>↓ STAT3/miR-214 pathway	-	B	[224, 225]
Ginsenoside Rg3	DSS-induced UC mice;<br>LPS+ATP-induced BMDMs	↑ Claudin-1, E-cadherin, Muc1, Occludin<br>↓Caspase-1	↓ IL-1β, IL-18, NLRP3	↑ *Actinobacteriota*, *g__Chlamydia*, *Paraprevotella*, *Prevotellaceae__NK3B31__group*, *Verrucomicrobiota*<br>↓ *Cyanobacteria*, *Clostridium__sensu__stricto1*, *Patescibacteria*, *Rikenellaceae__RC9__gut__group*	B	[226]
Ginsenoside Rg1	DSS-induced UC mice	-	↑Arg-1, IL-4, IL-10, M2 macrophages<br>↓ CCL-2, F4/80, IL-2, IL-6, IL-33, M1 macrophages, MIF-1, PIM-1, TLR2, TNF-α<br>↓ Nogo-B/RhoA pathway	↑ ACE, *Allobaculum*, *Akkermansia*, Chao, *Eubacterium_fissicatena_group*, *Lachnospiraceae*, *Lactobacillus*, *Norank_f_Muribaculaceae*<br>↓ *Bacteroides*, *Clostridia_UCG-014*, *Odoribacter*, *Proteobacteria*, *Turicibacter*	B	[227, 228]

Ginsenoside Rd	TNBS-induced UC rats;<br>DSS-induced UC mice	↑ GSH-Px, SOD<br>↓ Caspase-1, MDA	↑ AMPK/ULK1 pathway<br>↓ F4/80, iNOS, IFN-γ, IL-12/23p40, IL-17A, IL-1β, IL-6, MPO, NLRP3, NO, p-JNK, P-P38, p-65, p-ERK, TNF-α<br>↓ NF-κB pathway	-	B	[229-231]
Ginsenoside compound K	DSS-induced UC mice	↑ Occludin, ZO-1<br>↓ FITC-Dextran	↓ IL-1β, IL-17α, Th17/Treg cells, TNF-α	↑ *Akkermansia, Candidatus_ Saccharmonas, Patescibacteria, Ruminococcacee_UCG-014, Verrucomicrobia*<br>↓ *Proteobacteria*	H	[232]
Ginkgolide C	DSS-induced UC mice	↑Claudin-3, Goblet cells, Occludin, ZO-1	↓COX-2, iNOS, IL-1β, IL-6, MPO, TNF-α<br>↓ MAPK, NF-κB pathway	↑ Chao1, Shannon index, *Allobaculum, Lactobacillus, Lactobacillaceae, S24-7*<br>↓ *Alistipes, Bacteroides, Bacteroidaceae, Desulfovibrionaceae, Lachnospiraceae, Oscillospira, Prevotella, Prevotellaceae, Ruminococcus, Ruminococcaceae, Turicibacteraceae, Turicibacter*	B	[233]
Genistein	DSS-induced UC mice	↓ Caspase-1	↓ IL-1β, IL-18, MPO, TNF-α	-	A	[234]
Geniposide	DSS-induced UC mice;<br>DSS-induced UC rats;<br>LPS-induced BMDM cells;<br>LPS-induced RAW264.7 cells;<br>TNBS-induced UC rats;<br>LPS-treated Caco-2 cells;<br>TNBS-induced UC mice;<br>LPS-induced Caco-2 cells	↑ Cluadin-1, ccludin, SOD, ZO-1<br>↑ Nrf-2/HO-1 pathway<br>↓ Caspase-1, MDA, ROS	↑ IL-10, p-AMPK, p-ACC, PPARγ, STRT1<br>↓ CCL-2, COX-2, iNOS, IFN-γ, IL-1β, IL-6, IL-17, MPO, NLRP3, NOS2, p-p65, MLCK,, p-IκBα, TNF-α<br>↓ NF-κB, p38 MAPK pathway, regulate AMPK/MLCK pathway	-	B	[21, 235-239]

Garlicin	TNBS-induced UC rats	↓ Bcl-2	-	-	K	[240]
Gallotannin corilagin	DSS-induced UC mice	↓ Caspase-3, Caspase-9	↑ IκB-α<br>↓ IL-1β, IL-6, MPO, TNF-α	-	I	[241]
Gallic acid	DSS-induced UC mice;<br>LPS-induced RAW 264.7 cell;<br>TNBS-induced UC mice;<br>IL-1β-induced HIEC-6 cells	↓ Bcl-xl, Caspase-1, Caspase-4	↑ IκB-α, IL-4, IL-10,<br>↓ COX-2, ERK, iNOS, IFN-γ, IL-1, IL-1β, IL-6, IL-12, IL-17, IL-18, IL-23, IL-33, MPO, NO, p65, p-p65, NLRP3, TGF-β, TNF-α<br>↓ NF-κB pathway	-	I	[242-244]
Galangin	DSS-induced UC mice;<br>LPS-induced RAW 264.7 cells	↑ GST, GSH, SOD<br>↑ Nrf2/HO-1 pathway<br>↓ MDA, TBARS	↑ IL-10<br>↓ COX-2, HMGB1, iNOS, IL-1β, IL-6, MPO, NF-κB p65, TLR4, TNF-α<br>↓ NF-κB pathway	↑ Shannon index, Bacteroidetes/Firmicutes, SCFAs<br>↓ Simpson index	A	[245-247]
Fumigaclavine C	DSS-induced UC mice	↓ Caspase-1	↓ IL-1β, IL-17A, p-p65, p-STAT1, p-STAT3, TNF-α	-	C	[248]
Fucoxanthin	DSS-induced UC mice<br>LPS-induced RAW 264.7 macrophage	-	↓ COX-2, NF-κB, PGE2	-	B	[249]

Friedelin	DSS-induced UC mice	-	↑ IL-10, ATG5<br>↓ IL-6, IL-1β, MPO, p-MAPK, p-mTOR	-	B	[250]
Flavocoxid	Acetic acid-induced UC rats	↑ GSH, SOD<br>↓ Caspase-3, MDA	↓ iNOS, MPO, NOx, NF-κB/p65, TNF-α	-	A	[251]
Fisetin	DSS-induced UC mice;<br>LPS-induced mouse;<br>peritoneal macrophages	↑ GSH<br>↓ TBARS	↑ IκBα, p-ERK/ERK<br>↓ Akt, COX-2, iNOS, IL-1β, IL-6, MPO, NF-κB, p-IκBα, p-p38/p38, pAkt/Akt, TNF-α, regulate p38 MAPK<br>↓ NF-κB pathway	-	A	[252]
Ferulic Acid	TNBS-induced UC rats;<br>TNF-α-indeced HIMECs	↑ Bcl-2, GSH, SOD<br>↓ Caspase-1, Caspase-3, MDA	↑ IL-10<br>↓ COX-2, iNOS, IL-1β, IL-6, IL-12, MPO, NO, TNF-α<br>↓ TXNIP/NLRP3 pathway	-	G	[253, 254]
Evodiamine	DSS-induced UC mice;<br>DSS-induced UC rats	↑ Claudin-1, Claudin-2, Muc-2, Occludin, Tff3, ZO-1<br>↓ Caspase-1	↑ IL-10<br>↓ IL-1β, IL-6, IL-8, IκB, IFN-γ, MPO, Myd88, NLRP3, p-p65, p-IκBα, TLR4, TNF-α<br>↓ NF-κB pathway	↑ *Firmicutes*, *Lachnospiraceae*, *Ruminococcus*, SCFAs<br>↓ *Bacteroidetes*	C	[255-257]
Eupatilin	TNF-α-induced NCM460<br>LPS-induced THP-M<br>DSS-induced UC mice	↑ Occludin, ZO-1<br>↓ ROS	↑ p-AMPK/AMPK<br>↓ IL-1β, MPO, NOX4, TNF-α<br>↓ NF-κB, MAPK pathway	-	A	[258]

Ethyl rosmarinate	DSS-induced UC mice;<br>LPS-induced RAW264.7 cells	-	↓ IL-1β, IL-6, MPO, NO, TNF-α	-	I	[259]
Esculentoside A	TNBS-induced UC rats;<br>DSS-induced UC rats;<br>LPS-induced primary intestinal neuronal cells	-	↓ IL-6, nNOS, NO, TNF-α	-	H	[260, 261]
Eriodictyol	TNBS-induced UC rats;<br>DSS-induced UC mice	↑ Bcl-2, CAT, GSH-Px, Occludin, SOD, ZO-1<br>↓ MDA	↑ IL-10<br>↓ MPO, IL-1β, IL-2, IL-6, IL-12, IL-17, IL-23, TNF-α, regulate TLR4/NF-κB pathway	-	A	[262, 263]
Eriocitrin	DSS-induced UC mice	↓ MMP-9	↓ COX-2, iNOS, IL-1β, IL-6, MPO, NF-κB , TNF-α	-	A	[264]
Erianin	DSS-induced UC mice	↑ SOD<br>↓ ROS	↓ IL-1β, IL-6, IL-8, IFN-γ, IκBα, TNF-α, TRAF6<br>↓ NF-κB, TLR4, STAT3, Jak2/STAT3 pathway	-	K	[265]
Ergothioneine	DSS-induced UC mice;<br>DSS-induced UC rats	↑ Occludin	↓ CD4+ T cells, IL-1β, IL-6, MPO, MyD88, NF-κB p65, TNF-α, TLR4<br>↓ TLR4/MyD88/NF-κB pathway	-	C	[266, 267]
Epoxymicheliolide	DSS-induced UC mice;<br>LPS-induced RAW264.7 cells	↑ Occludin<br>↑ Keap1-Nrf2 pathway<br>↓ MDA, ROS	↓ COX2, iNOS, IL-1β, IL-6, MPO, NO, TNF-α	-	B	[268]

			↓ TAK1-NF-κB, NF-κB pathway			
Epigallocatechin gallate	DSS-induced UC mice	↑ GLP-2, Muc2, Nrf2, Occludin, ZO-1<br>↓ MDA	↑ IL-10, TGF-β,<br>↓ IL-1β, IL-6, IL-17A, MCP-1, TNF-α	-	I	[269-271]
Epicatechin	DSS-induced UC mice;<br>LPS-induced RAW 264.7 cells	↑ CAT, GSH-Px, SOD<br>↓ MDA	↓ IL-6, MPO, NO, TNF-α<br>↓ NF-κB pathway	-	I	[272]
Emodin	DSS-induced UC mice;<br>LPS-induced RAW 264.7 cells;<br>IFN-γ + IL-22-induced Caco-2 cells;<br>Flagellin-inducedd HT-29 cells	-	↑ IκB, PPARγ<br>↓ iNOS, IL-1β, IL-6, MPO, MyD88, NOS2, p65, TLR4, TLR5<br>↓ PI3K/Akt pathway, regulate flagellin/TLR5, TLR5/NF-κB, TLR4/NF-κB, PPARγ pathway	↑ Cyanobacteria, Peptococcaceae and Rikenellaceae, Shannon index<br>↓Aerococcaceae, Proteobacteria, Enterobacteriaceae, Enterococcaceae, Lactobacillaceae	D	[86, 273-275]
Ellagic Acid	DSS-induced UC mice	-	↓ COX-2, iNOS, IFN-γ, IL-6, p-IκBα, NF-κB p65, p-p38/p38, p-STAT3/ STAT3, TNF-α	-	I	[276]
D-Pinitol	DSS-induced UC mice	↑ CAT, GSH, SOD<br>↑ Nrf2/ARE pathway<br>↓MDA	↑ IL-10<br>↑ PPARγ/NF-κB pathway<br>↓ COX-2, iNOS, IFN-γ, IL-1β, IL-6, IL-17, MPO, NF-κB p65, IκBα, TNF-α	-	K	[277]

Docosapentaenoic acid	DSS-induced UC mice	-	↑ IL-10, PGE2<br>↓ 5-LOX, COX, IL-1β, IL-6, LTB4, MPO, TNF-α	↑ ACE, Chao index, Shanno index<br>↑ *Bacteroidetes, Firmicutes*<br>↓ *Firmicutes/Bacteroidetes*	K	[278, 279]
D-limonene	DSS-induced UC rats	↑ GSH, SOD<br>↓ MMP-2, MMP-9	↑ p-ERK1/2<br>↓ COX-2, iNOS, IL-1β, IL-6, NF-κB p65, TGF-β, TNF-α	-	B	[280]
Diplacone	DSS-induced UC rats	↑ pro-MMP2/MMP2	↓ COX-2	-	A	[165]
Diosmin	Acetic acid-induced UC rats	↑ GSH<br>↓ MDA, Caspase-3	↓ COX-2, MPO, TNF-α	-	A	[281]
Diosgenin	TNBS-induced UC rats	↑ GSH, SOD<br>↓ MDA<br>↓ Bax/Caspase-1 pathway	↑ IL-10<br>↓ COX-2, iNOS, IκBα, IL-1β, IL-6, IFN-γ, MPO, NO, TNF-α<br>↓ NF-κB/IκB-α pathway	-	E	[282]
Dioscin	DSS-induced UC mice; LPS+ IFN-γ-induced RAW 264.7 cells	↑ GSH, Occludin, SOD, ZO-1<br>↓ Caspase-1, FITC-Dextran, HIF-1α, MDA	↑ IL-10, p-AMPK<br>↑ mTORC2/PPARγ pathway<br>↓ CD80, iNOS, IFN-γ, IL-1β, IL-6, MPO, NLRP3, NO, p-mTOR, p-p38, TNF-α<br>↓ MAPK, mTORC1/ HIF-1α, NF-κB pathway	-	H	[283-285]

Dihydrotanshinone I	DSS-induced UC mic; LPS-induced RAW 264.7 cells	↑ Caspase-8	↓ COX-2, HMGB1, iNOS, IL-1β, IL-6, MPO, MLKL, RIP1, RIP3, TNF-α	-	B	[286]
Dihydroberberine	DSS-induced UC mice	↑ Claudin-1, JAM-A, Muc1, Muc2, Occludin, ZO-1, ZO-2	↓ IFN-γ, IL-1β, IL-6, IL-17, TNF-α, MPO<br>↓ TLR4/MyD88/NF-κB pathway	-	C	[287]
Dihydroartemisinin	DSS-induced UC mice	↑ Occludin, ZO-1	↑ IL-10<br>↓ IL-1β, IL-4, IL-6, IL-17, TNF-α<br>↓ JAK2/STAT3, NF-κB, PI3K/AKT pathway	-	B	[288, 289]
Didymin	DSS-induced UC mice	-	↑ IL-10, M2 Macrophages<br>↓ IL-6, M1 Macrophages, MPO, NOS2, TNF	-	A	[290]
Diammonium Glycyrrhizinate	Acetic acid-induced UC rats	↓ ICAM-1	↓ MPO, NF-κB p65, TNF-α	-	B	[291]
Diacetylrhein	DSS-induced UC mice	↑ Bcl-2/Bax, Bcl-2, Occludin, ZO-1<br>↓ Caspase-3, Bax, Caspase-1 activity, MDA, ROS	↑ IL-10<br>↓ IL-1β, IL-6, IL-18, MPO, MCP-1, NLRP3, NF-κB DNA binding activity, TGF-β	-	D	[292]

Desmethylbellidifolin	TNBS-induced UC rats;<br>LPS-induced RAW 264.7 cells;<br>DSS-induced UC mice<br>Caco-2 cells	↑ Claudin-2, GSH, Occludin, ZO-1	↓ COX-2, iNOS, IL-6, MPO, NO, TNF-α	↓ Bacteroidaceae	A	[293, 294]
Deoxyschizandrin	DSS-induced UC mice	↑ Bcl-2, CAT, SOD<br>↓ Bax, Caspase-3, MDA	↓ IL-1β, IL-6, MyD88, TNF-α<br>↓ TLR4/NF-κB pathway	-	G	[295, 296]
Demethyleneberberine	DSS-induced UC rats	↓ Caspase-1	↓ IL-1β, NLRP3, TNF-α<br>↓ NF-κB pathway	-	C	[297]
Dehydrocostus Lactone	DSS-induced UC mice	↑ Muc2<br>↓ α-Defensin, SOD, XBP1s	↓ IL-1β, IL-6, IL-17, IL-23, MCP-1, MPO, TNF-α<br>↓ IL-6/STAT3 Pathway	-	B	[298]
Daidzein	DSS-induced UC mice<br>LPS-induced RAW 264.7 cells	-	↓ IL-1β, IL-6, MPO, NO, TNF-α<br>↓ NF-κB pathway	-	A	[299]
Cyclosporine	DSS-induced UC mice<br>Caco-2 cells	↓ Caspase-8	↑ IL-10, TGF-β	-	C	[300, 301]
Cycloastragenol	Acetic acid-induced UC rats	↑ Bcl2<br>↓ BAX, Caspase-3	↓ MIP-1α, SphK, TNF-α<br>Regulate SphK/MIP-1α/miR-143 pathway	-	B	[302]

Curcumin	DSS-induced UC mice;<br>Acetic acid-induced UC rats;<br>TNB-induced UC mice	↓ Apoptotic epithelial cells<br>↓ ATP, CAT, HIF-1α, MDA	↑ IL-10, IL-33, M2 macrophages, mTOR, SIRT1, STAT3, TGF-β<br>↓ CCL2, CD4 T cells, CD8 T cells, F4/ 80, Foxp1, IL-1, IL-1β, IL-6, IL-12, IL-17α, IL-23, M1 macrophages, MCP-1, MPO, p-STAT3, TGF-β, Th17 cells, Th17/Treg cells, TNF-α<br>↓ TLRs pathway, p38MAPK pathway	-	I	[89, 303-308]
Corynoline	DSS-induced UC mice	↑ CAT, GSH/GSSG, SOD<br>↑ Nrf2 pathway	↓ IL-1β, IL-6, MPO, TNF-α<br>↓ NF-κB pathway	-	C	[309]
Corylin	DSS-induced UC mice	↑ ZO-1, Occludin	↓ IL-6, TNF-α	↑ *Candidatus_Stoquefichus*, *Dubosiella*, *Enterorhabdus*<br>↓ *Bacteroides*, *Escherichia-Shigella*, *Turicibacter*	A	[310]
Coptisine	DSS-induced UC mice	↑ Bcl-2, Claudin-1, Occludin, ZO-1, ZO-2<br>↓ Bax, Bax/Bcl-2, Caspase-3, ICAM-1, VCAM-1	↑ IL-10, TGF-β<br>↓ IL-1β, IL-6, IL-17, IFN-γ, MPO TNF-α<br>↓ NF-κB pathway	-	C	[311]

Convallatoxin	DSS-induced UC mice;<br>LPS-induced RAW264.7 cells and BMDMs	-	↑ PPARγ<br>↓ COX-2, iNOS, IL-1β, IL-6, NF-κB p65, TNF-α<br>↓ NF-κB pathway	-	E	[312]
Citrulline	TNBS+Ethyl alcohol-induced UC rats	-	↓ CD68, IL-6, IL-17A, MCP-1, p-STAT3	-	C	[313]
Cinnamtannin D1	DSS-induced UC mice	-	↑AMPK/mTOR pathway<br>↓ IL-1β, IL-6, MPO, TNF-α, restore the balance of Th17-Treg cells	-	I	[314]
Cinnamaldehyde	DSS-induced UC mice<br>LPS-induced RAW264.7 cells<br>DSS+ *Candida albicans*-induced UC mice	↓ Caspase-1, ROS	↑ IL-10<br>↓ CDC42, COX-2, Dectin-1, IL-1β, IL-6, IL-8, IL-17A, IL-18, MPO, NF-Kb, NLRP3, NO, STAT3, Th17, TLR2, TNF-α<br>↓ TLR4/NF-κB pathway	-	G	[315-318]

			Regulate dectin-1/TLRs/NF-κB pathway			
Chelidonic acid	DSS-induced UC mice	-	↓ COX-2, HIF-1α	-	K	[319]
Cepharanthine	DSS-induced UC mice	-	-	↑ *Acetatifactor*, *Family_XIII_AD3011_group*, *Ruminococcaceae_N-K4A214_group*<br>↓ *Escherichia-Shigella*, *Romboutsia*, *Romboutsia*, *Turicibacter*	C	[320]
Celastrol	DSS-induced UC mice	↑ Caspase-8, CDH1, E-cadherin, Muc2, Occludin, ZO-1	↑ IL-10, NOx, TGF-β,<br>↓ IFN-γ, IL-1β, IL-6, IL-17A, IL-23, MPO, MLKL, Th1, Th17, TNF-α	↑ *Alloprevotella*, *Butyricicoccus*, *Paraprevotella*, *Prevotellaceae*	B	[320-322]
Cavidine	Acetic acid-induced UC mice<br>LPS-induced peritoneal macrophages	↑ GSH, SOD<br>↓ MDA	↓ IL-6, MPO, TNF-α<br>↓ NF-κB pathway	-	C	[323]
Caulerpin	DSS-induced UC mice	-	↓ IFN-γ, IL-6, IL-17, TNF-α<br>↓ NF-κB pathway	-	C	[324]

Catechin-7-O-β-D-glucopyranoside	TNBS-induced UC rats	↑ GSH, Muc2, Muc3<br>↓ MDA, ICAM-1	↓ COX-2, iNOS, IL-1β, MCP-1, MPO, TNF-α<br>↓ p38 MAPK-NF-κB pathway	-	A	[325]
Casticin	DSS-induced UC mice;<br>LPS-induced RAW264.7 cells;<br>H2O2-treated Caco-2 cells	↑ E-cadherin, GSH, ICAM-1, SOD<br>↓ MDA, ROS	↓ CD4+ cells, iNOS, IL-1β, IL-6, MPO, TNF-α<br>↓ AKT/NF-κB pathway	-	A	[326]
Carnosol	DSS-induced UC mice;<br>Thapsigargin -induced HCT-116 cells	↑ Claudin-1, Muc2, Occludin, Tff3, ZO-1	↑ IEL subpopulations<br>↓ Dendritic cell, IFN-γ, TNF-α, IL-6, IL-1β, monocytes/macrophages, neutrophils	-	B	[327]
Carnosic acid	DSS-induced UC mice	↑ GCLM, GPX2, GSH, HO-1, Nrf2, SOD, SOD2<br>↓ Caspase-1, MDA<br>regulate Keap1/Nrf2 pathway	↓ F4/80, IFN-γ, IL-6, IL-1β, IL-17A, IL-18, MPO, TNF-α<br>↓ C-Jun, NF-κB pathway	-	B	[328]
Cardamonin	Acetic acid-induced UC rats	↓ MDA	↓ iNOS, MPO, NF-κB, TNF-α	-	A	[329]

Capsaicin	DSS-induced UC mice	-	↓ IFN-γ, IL-17A, IL-22, MPO	↑ *Ruminoclostridium_1*, *Oscillibacter*, *Marvinbryantia*, *Faecalibaculum*<br>↓ *Bacteroidales*	C	[330, 331]
Cannabidivarin	DNBS-induced UC mice	↓ FITC-Dextran	↑ TRPV1<br>↓ IL-1β, IL-6, MCP-1, MPO, TRPA1, TRPV2	↑ Bacteroidales, Proteobacteria	I	[332]
Camptothecin	DSS-induced UC mice;<br>LPS-induced RAW264.7 cells	-	↓ COX-2, iNOS, IL-1β, IL-6, MPO, TNF-α<br>↓ AKT/NF-κB, MAPK pathway	-	C	[333]
Caffeic acid phenethyl ester	DSS-induced UC mice;<br>LPS-induced RAW264.7 cells	↓ ICAM-1, VCAM	↓ IFN-γ, IL-1β, IL-6, IL-17, MPO, NF-κB p65, p-IκB-α, TNF-α	-	G	[334, 335]
Caffeic acid	DSS-induced UC mice;<br>LPS-induced primary BMDMs and BMDCs	-	↓ CD11; F4/80; IL-1β, IL-6, IL-12, IFN-γ, TNF-α<br>↓ NF-κB pathway	↑ *Akkermansia*, *Verrucomicrobia*<br>↓ *Firmicutes/Bacteroidetes*	G	[336, 337]
Butyrate	Acetic acid-induced UC rats;<br>TNBS-induced UC rats;<br>DSS-induced UC mice	↑ Goblet cells	↑ IL-10, IL-10/IL-12, TGF-β<br>↓ IL-6, IL-12, IL-17, TNF-α<br>↓ CCR9/NF-κB pathway	↑ SCFAs	K	[338-340]

Bryodulcosigenin	DSS-induced UC mice;<br>NCM460 cells;<br>MLE-12 cells	↑ Occludin, ZO-1<br>↓ Bax/Bcl, Caspase-3, Caspase-9	↑ IκBα, IL-10<br>↓ IL-Iβ, IL-6, IL-17, NLRP3	-	B	[341]
Brusatol	TNBS-induced UC rats;<br>LPS-induced RAW 264.7 macrophages	↑ CAT, GSH, Nrf2, SOD<br>↓ Caspase-1, Keap-1	↑ TGF-β, IL-4<br>↓ IL-1β, IL-18, NO, NF-κB p65, NLRP3, TNF-α<br>↓ NF-κB pathway	-	B	[342]
Bruceine D	TNBS-induced UC rats	↑ GSH-Px, SOD<br>↓ MDA, ROS	↑ IL-10, TGF-β<br>↓ COX-2, iNOS, IL-1β, IL-6, IL-8, MPO, MyD88, NF-κB p65, TLR4, TNF-α, TRAF-6	-	B	[342]
Boldine	DSS-induced UC mice	↑ CAT, SOD<br>↓ MDA	↑ IκB-α<br>↓ CD 68+, IL-6, IL-17, MPO, p65-NF-κB , TNF-α	-	C	[343]
Bilobalide	DSS-induced UC mice;<br>LPS-induced RAW 264.7 Cells	↑ Claudin-3, Occludin, ZO-1	↓ COX-2, iNOS, IL-1β, IL-6, MPO, TNF-α<br>↓ AKT/NF-κB, MAPK pathway	↑ *Firmicutes*, *Lactobacillus*<br>↓ *Bacteroidetes*, *Dubosiella*	B	[344]

Betulinic acid hydroxamate	DSS-induced UC mice; TNBS-induced UC mice; Caco-2 cells	↑ Claudin-1, Muc2, Muc3, ZO-1 ↓ MMP-3, MMP-8, Mrc-1	↑ IL-13 ↓ CD3, F4/80, HIF-1α, IL-1β	-	B	[345]
Betulin	Acetic acid-induced UC rats	↓Caspase-8, Caspase-3	↓ CD68 cells, IL-6, IL-1β, NF-κB, TLR4, TNF-α	-	B	[346]
Betaine	DSS-induced UC mice	↑ CAT, caspase-1, GSH, GSH/GSSG, Nrf2 ↓ GSSG, MDA, SOD1	↓ COX-2, IL-1β, IL-6, IL-18, MPO, NLRP3, NOS1, NOS2, NOS3	-	C	[347]
Bergenin	DSS-induced UC mice; LPS-induced RAW 264.7 cells; TNBS-induced UC rats	↓ Caspase 11	↑ IκB, PPARγ, SIRT1 ↑ NF-κB pathway ↓ COX-2, iNOS, IFN-γ, IL-1β, IL-6, IL-18, MPO, NFκB-p65, p-STAT3, TNF-α ↓ NLRP3 Regulate PPARγ/SIRT1/NF-κB-p65 pathway	-	G	[348, 349]
Berberrubine	DSS-induced UC mice	↑ Bcl-2, Claudin-1, Muc1, Muc2, Occludin, ZO-1, ZO-2 ↓ Bax/Bcl-2	↓ IFN-γ, IL-1β, IL-4, IL-6, MPO, TNF-α	-	C	[350]

Berberine hydrochloride	DSS-induced UC rats	↑ Claudin-1, Occludin, ZO-1, VCAM-1	↑ IL-4, IL-10<br>↓ IL-1, IL-1β, IL-6, IL-12, TNF-α, TGF-β, IFN-γ	-	C	[351]
Berberine	Acetic acid-induced UC rats;<br>Alcohol-induced UC rats;<br>DSS-induced UC mice;<br>DSS-induced UC rats;<br>TNBS-induced UC rats;<br>DSS- induced UC cats;<br>LPS-induced HT29 cells;<br>LPS-induced Raw 264.7 cells;<br>LPS-induced inflammatory damage of NCM460 cells;<br>LPS-induced IEC-18 Inflammatory Model;<br>TNF-α-primed Caco-2 cells monolayers	↑ Bcl-2, CAT, E-cadherin, GPX, GR, GSH, Muc2, Occludin, SOD, ZO-1<br>↑ Nrf2/HO-1 pathway<br>↓ Bax, Caspase-1, Caspase-12, Caspase-3, FITC-Dextran, GRP78, ICAM-1, IECs apoptosis, LBP, LPO, MDA, MMP-9, MadCAM-1<br>↓ Caspase12/caspase-3 apoptosis pathway	↑ IL-10, IL-13, IL-4<br>↑ p38 MAPK, Wnt/β-catenin pathway<br>↓ Dendritic cells, NK cells, NKp46, COX-2, GATA3, iNOS, IFN-γ, IL-1β, IL-2, IL-5, IL-6, IL-17, IL-17A, IL-18, IL-23, IL-23R, ILC1, JNK, MAPK14, MPO, NLRP3, NO, NOS2, neutrophils, p-STAT3, Th17, TNF-α,<br>↓ p-JAK2/STAT3, TLR4/NF-κB pathway<br>Regulate AMPK/MTOR/ULK1 pathway	↑ ACE, Chao index<br>↑ Bacillibacteria, *Bacteroides*, *Bacteroides fragilis*, *Eubacterium*, *Lactobacillius/Lactococcus*, *Sutterella*<br>↓*Allobaculum*, *Akkermansia*, *Bacteroides*, *Desulfovibrio*, Enterobacteriaceae, *Mucispirillum*, *Oscillospira*, Verrucomicrobiales	C	[62, 352-364]
Barbaloin	DSS-induced UC rats;<br>LPS-induced Caco-2 cell	↑ E-cadherin, Occludin, ZO-1	↑ AMPK, IL-10, IL-4<br>↓ IFN-γ, IL-1β, IL-6, MLCK, TNF-α, Regulated the AMPK/MLCK pathway	-	D	[365]

Baicalin	LPS-induced RAW 264.7 cells; TNBS-induced UC rats; DSS-induced UC mice	↑ ATP, Bcl-2, CAT, GSH-Px, Occludin, SOD, ZO-1<br>↓ Bax, Bcl-2/Bax, Caspase-3, Caspase-9, Cyt-c, Fas, FasL, ICAM-1, MDA, ROS	↑ Foxp3, IL-10, TGF-β, PPARγ, Treg cells<br>↓ COX-2, F4/80, IFN-γ, iNOS, IL-1β, IL-6, IL-12, IL-13, IL-17, IL-33, MCP-1, MIP-3α, MPO, NF-κB p65, NO, p-IκB-α, TLR2, TLR4, TLR 9, Th17/Treg cells, TNF-α<br>↓ CD14/TLR4/NF-κB, IKK/IKB/NF-kB, MAPK, NF-κB, TLR4/NF-κB-p65/IL-6, TLR4/NF-κB<br>regulate PPARγ pathway	↑ Shannon index, *Firmicutes*, SCFAs<br>↓ *Actinobacteria*, *Firmicutes/Bacteroidetes* ratios, *Proteobacteria*	A	[273, 354, 366-375]
Procyanidins	DSS-induced UC mice	↑ Claudin-1, Muc2, Occludin, SOD<br>↓MDA	↑ IL-10<br>↓ COX-2, iNOS, IL-1β, IL-6, MPO, NO, TNF-α	↑ *Anaerotruncus*, *Clostridium XlVb*, SCFAs<br>↓ *Alistipes*	K	[376, 377]
Atractylodin	DSS-induced UC mice; LPS-induced RAW264.7 cells	-	↓ IL-6, IL-1β, iNOS, TNF-α<br>↓ MAPK Pathway	↑ *Akkermansia*, *Alistipes*, *Firmicutes*, *Muribaculum*<br>↓ *Bacteroides*, *Deferribacteres*, *Desulfovibrio*, *Flavonifractor*, *Mucispirillum*, *Proteobacteria*	K	[378]

Atractylenolide I	DSS-induced UC mice	-	↓ PI3K, AKT	↑ Chao1, Shannon, Simpson index<br>↑ *Erysipelatoclostridium*, *Firmicutes*, *Lactobacillus*, *Lachnospiraceae*,<br>↓ *Enterobacter*, *Helicobacter*, *Proteobacteria*, *Rodentibacter*, *Shigella*	B	[379]
Atractylenolide III	DSS-induced UC mice;<br>LPS-induced IEC-6 cells	↑ GSH, Occludin, SOD, ZO-1<br>↓MDA	↑ AMPK/SIRT1/PGC-1α pathway<br>↓ COX-2, iNOS, IL-6, TNF-α	-	B	[380]
Astragaloside IV	DSS-induced UC mice;<br>LPS-induced CCD-18Co cells	↑ CAT, Claudin-1, GSH-Px, SOD, ZO-1<br>↓ MDA	↑ Foxp3, IL-10, TGF-β1, STAT5, Treg cells<br>↓ AHR, IL-17, IL-1β, IL-21, IL-6, MPO, NO, TNF-α, Th17 cells<br>↓ NF-κB, Notch pathway	-	B	[381-383]
Astragaloside II	DSS-induced UC mice;<br>LPS-induced UC CCD-18Co cells	↑ SOD<br>↓ MDA	↓ IL-1β, IL-6, MPO, NO, TNF-α<br>↓ HIF-α/NF-κB pathway	-	H	[384]
Astragalin	DSS-induced UC mice	↑ Muc2, Occludin, ZO-1	↓ COX-2, IFN-γ, IL-1β, IL-6, MCP-1, MPO, TLR4, TNF-α<br>↓ NF-κB pathway	↑ ACE, Shannon index<br>↑ *Butyricicoccus*, Lachnospiraceae, *Family_XIII_UCG-001*, *Oscillibacter*, *Ruminiclostridium_9*, *Ruminiclostridium*, *Ruminococcaceae_NK4A214_group*, *Ruminococcaceae_UCG-009*, *Peptococcus*, Ruminococcaceae, *Unclassified _f__ Lachnospiraceae*<br>↓ Bacteroidaceae, *Escherichia-Shigella*, *Ruminococcus_1*, Erysipelotrichaceae, Prevotellaceae, Peptostreptococcaceae, norank_o__Rhodospirillales	A	[385]

Asperuloside	DSS-induced UC mice;<br>LPS-induced RAW 264.7 cell	↑ GSH-Px, SOD<br>↑ Nrf2/HO-1 pathway<br>↓ ROS	↑ IL-10<br>↓ IL-6, MPO, TNF-α<br>↓ NF-κB pathway	-	B	[386]
Aspartate	DSS-induced UC mice	↑ GPX4	↑ PIPK3, MLKL<br>↓ IL-1β, RIPK1, TNF-α	↑ *Alistipes*, *Bacteroidetes*, *Lactobacillus*<br>↓ *Actinobacteria*, *Akkermansia*, *Verrucomicrobia*	C	[387]
Artesunate	TNBS+Ethyl alcohol-induced UC mice;<br>LPS-induced RAW264.7 cells;<br>DSS-induced UC mice;<br>DSS-induced UC rats	↑ Bcl-2/Bax<br>↓ Bax, Caspase-3, Caspase-9, Caspases-12	↑ IL-10<br>↓ IFN-γ, IL-1β, IL-6, IL-8, IL-12, IL-17, IL-23, MPO, TNF-α<br>↓ TLR4-NF-κB pathway	-	B	[388-391]
Artemisinin	DSS-induced UC rats	-	↑ PPARγ<br>↓ NF-κB	-	B	[392]
Aromatic-turmerone	Acetic acid-induced UC mice	-	↓ COX-2, TNF-α	↑ *Lachnospiraceae*, *Muribaculaceae*, *Ruminococcaceae*, uncultured-bacteroidales-bacterium-g-alloprevotella<br>↓ gut_metagenome_G_ alloprevotella	B	[393]
Arctigenin	DSS-induced UC mice	↑ GSH, ICAM-1, SOD<br>↓ MDA, MAdCAM-1, VCAM-1, E-selectin	↓ IL-6, MCP-1, MIP-2, MPO, TNF-α<br>↓ MAPK, NF-κB pathway	-	G	[394]
Arbutin	DSS-induced UC mice	↑ Bcl-2, Claudin-1, Occludin, ZO-1<br>↓ Bax, PARP	↑ IL-10<br>↓ IL-6, MPO, TNF-α<br>↓ MAPK/ELK1 pathway	-	K	[395]

Apocynin	DSS-induced UC mice	↓ ROS	↓ COX-2, iNOS, IFN-γ, IL-1β, IL-6, NO, NOXs, TNF-α<br>↓ NOXs-ROS-p38MAPK pathway	-	K	[396]
Apigenin	Acetic acid-induced UC mice;<br>DSS-induced UC mice;<br>Acetic acid-induced UC rats	↑ Claudin-1, Goblet cells, GSH, Occludin, ZO-1<br>↓ Caspase-1, Caspase-11, MDA, MAdCAM-1	↑ IL-10<br>↓ COX-2, iNOS, IL-1β, IL-6, IL-18, MPO, mPGES, MMP-3, TNF-α	↑ ACE, Chao1 index, SCFAs<br>↑ *Lactobacillus*, *Akkermansia*, *Dubosiella*, *Firmicutes*, *Faecalibaculum*, *Verrucomicrobia*<br>↓ *Bacteroides*, *Proteobacteria*, *Turicibacter*, *Klebsiella*, *Romboutsia*	A	[397-400]
Anemoside B4	DSS-induced UC mice;<br>LPS-induced RAW 264.7 cell;<br>TNBS-induced UC mice	↓ Bcl-2/Bax, Caspase-3	↓ IL-1β, IL-6, IL-17, MPO, NO, TNF-α<br>↓TLR4/ NF-κB/MAPK, S100A9/MAPK/NF-κB pathway	↑ *Akkermansia*<br>↓ *Bacteroidetes*	H	[401-403]
Anemonin	DSS-induced UC mice	-	↓ IL-1β, IL-6, TNF-α	-	K	[404]
Andrographolide sodium bisulfite	DSS-induced UC mice	↑ α-Catenin, β-Catenin, ZO-1<br>↓ YAP	↓ IL-1β, IL-6, IL-17A, TNF-α	-	B	[405]
Andrographolide	Oxazolone-induced UC rats	-	↓ IL-13, IL-4, MPO, NF-κB p-p65, TNF-α<br>↓ IL-4R–STAT6 pathway	-	B	[406]

Amentoflavone	Acetic acid-induced UC rats	↑ GSH, SOD<br>↓ LPO, LDH	↓ COX-2, iNOS, IL-1β, IL-6, MPO, NO, TNF-α<br>↓ NF-κB pathway	-	A	[407]
Alpinetin	DSS-induced UC mice;<br>TNF-α-stimulated Caco -2 cells	↑ Occludin, occludens-1, claudin -7, SOD, ZO-1<br>↑ Nrf2/HO-1 pathway<br>↓ Caspase-3, Claudin-2, IECs apoptosis, MDA<br>↓ mTORC1 pathway	↑ Treg cells, Foxp3, IL-10<br>↓ IL-1β, IL-17, MPO, RORγt, TNF-α	-	A	[408-410]
Alliin	DSS-induced UC mice<br>LPS-induced 264.7 cells	↓ MDA	↓ ERK, iNOS, IL-6, IL-1β, JNK, MPO, NO, p38, TNF-α<br>↓ MAPKs-PPARγ/NF-κB/AP-1/STAT-1 pathway	-	C	[411]
Aloin A	DSS-induced UC mice<br>LPS-induced LS174T cells	↑ Muc2, Occludin<br>↓ Caspase-3<br>↓ Notch/Hes1 pathway	↑ IL-10<br>↓ IL-1β, MPO, TNF-α	-	D	[412]
Albiflorin	DSS-induced UC mice	↑ GSH, SOD<br>↓ MDA	↑ Foxp3, STAT5<br>↓ IL-1β, IL-6, MPO, TLR4, TNF-α<br>↓ NF-κB, MAPK pathway	-	K	[413]
Alanyl-glutamine	DSS-induced UC mice	↑ Bcl-xL, Muc2, Tff3	↑ IκBα/NF-κB p65<br>↓ IFN-γ, IL-4, IL-17A, IL-17F, TLR4, TNF-α	-	K	[414, 415]

Aesculin	DSS-induced UC mice;<br>LPS-induced RAW 264.7 cells	-	↑ PPARγ pathway<br>↓ iNOS, IL-1β, TNF-α, NO<br>↓ NF-κB pathway	-	G	[416]
Acteoside	DSS-induced UC mice;<br>DSS-treated Caco-2 cells	↑ Bcl-2, CAT, GSH, HO-1, Occludin, SOD, ZO-1<br>↓ Bax, Caspase-3, Claudin-2, MDA	↓ IL-1β, IL-6, TNF-α	-	G	[417]
Acanthoic acid	DSS-induced UC mice	-	↓ COX-2, MPO, TNF-α	-	B	[418]
Acacetin	DSS-induced UC mice;<br>LPS-induced RAW264.7 cells	-	↓ COX-2, iNOS, IL-1β, IL-6, NO, TNF-α	↑ Shannon index, *Firmicutes*<br>↓ *Bacteroidaceae*, *Deferribacteres*, *Deferribacteraceae*, *Enterobacteriaceae*, *Escherichia-Shigella*, *Faecalibaculum*, *Proteobacteria*	A	[419]
8-Oxypalmatine	DSS-induced UC mice	↑ CAT, GSH, GSH-Px, HO-1, Nrf2, SOD, T-AOC<br>↓ MDA	↑ IL-10<br>↓ IFN-γ, IL-1β, IL-6, IL-17A, MPO, NLRP3, NO, TNF-α	-	C	[420]
8-Gingerol	DSS-induced UC rats	↑ SOD<br>↓ MDA	↓ MPO	-	I	[421]

6-Shogaol	DSS-induced UC mice	-	↓ IL-1β, IL-6, TNF-α	↑ ACE, Chao, Shannon, Simpson index<br>↓ Verrucomicrobia, Verrucomicrobiae, Verrucomicrobiales	I	[422]
6-Paradol	Acetic acid-induced UC rats	↑ GSH<br>↓ MDA	↓ MPO	-	I	[423]
6-Gingerol	DSS-induced UC mice;<br>DSS-induced UC rats	↑ CAT, GPX, GSH, GST, SOD<br>↓ H2O2, MDA	↑ Foxp3, IL-10<br>↓ COX-2, iNOS, IL-17, IL-1β, IL-6, MCP-1, MPO, NF-κB, p38, NO, TNF-α<br>↓Wnt/-catenin pathway	-	I	[421, 424-426]
6,7-Dihydroxy-2,4-Dimethoxyphenanthrene	DSS-induced UC mice	↑ Occludin<br>↓ Caspase-3, Caspase-8	↓ ERK, IFN-γ, IL-23, MPO, NO<br>↓ NF-κB/COX-2 pathway	-	K	[427]
4-Geranyloxy-2,6-Dihydroxybenzophenonel	DSS-induced UC mice	↑ E-cadherin, ZO-1	↑ PKA/CREB pathway<br>↓ COX-2, iNOS, IFN-γ, IL-1β, IL-6, TNF-α, MPO<br>↓ NF-κB pathway	-	K	[428]
3,4-Oxo-isopropylidene-shikimic acid	TNBS-induced UC rats;<br>Acetic acid-induced UC rats;<br>TNBS-induced UC mice	↑ GSH, GSH-Px, ICAM-1, SOD<br>↓MDA,	↓ IFN-γ, iNOS, IL-1β, IL-8, MPO, NF-κB p65, NO, TNF-α	-	K	[429, 430]

3,3'-Diselenodipropionic acid	DSS-induced UC mice	-	↓ F4/80, IL-1β, IL-6, IL-17A, IL-17F, NLRP3, p-STAT3/STAT3, TNF-α	-	K	[431]
2,3,5,4'-Tetrahydroxystilbene-2-O-β-D-glucoside	DSS-induced UC mice	↑ Occludin, ZO-1	↑ IL-10<br>↓ IL-1β, IL-6, TNF-α	↑ ACE, Chao1 index, Bacteroidetes, Firmicutes, Lachnospiraceae_NK4A136<br>↓ Simpson index, Bacteroides, Firmicutes/Bacteroidetes ratio, Helicobacter, Parabacteroides, Proteobacteria	K	[432]
14-O-Acetylneoline	TNBS-induced UC mice	-	↓ IFN-γ	-	C	[433]
11-Hydroxy-1'-O-Methylamentadione	DSS-induced UC mice	-	↓ COX-2, iNOS, IL-1β, MPO, TNF-α	-	B	[434]
10-Gingerol	DSS-induced UC rats	↑ SOD<br>↓ MDA	↓ MPO	-	I	[421]
1,25-Dihydroxyvitamin D3	DSS-induced UC mice; ATP+LPS-induced peritoneal macrophages	↓ ROS	↑ M2 macrophages<br>↓ CD4+ T cells, Dendritic cells, IFN-γ, IL-1β, IL-6, IL-17, IL-18, M1 macrophages, MPO, NLRP3, Th1, Th17, TNF-α	-	K	[435, 436]

Linalool	Acetic acid-induced UC rats	↑ CAT, GPX, Nrf2<br>↓ MDA	↓ COX-2, IL-1β, NF-κB	-	B	[171]
Chlorogenic Acid	DSS-induced UC mice;<br>LPS/ATP-induced RAW264.7 cells	↑ Bcl-2, SOD<br>↓ Bax, Caspase-1, Caspase-3	↑ IL-10<br>↓ IL-1β, IL-18, IL-6, MPO, NLRP3, NO, PAF, p-NF-κB, TNF-α<br>↓ MAPK/ERK/JNK, NF-κB/NLRP3 pathway	↑ Simpson index<br>↑ *Akkermansia*, *Bifidobacterium*, *Clostridium_sensu_stricto_1*, *Firmicutes/Bacteroidetes*, *Dubosiella*, *Lactobacillus*, *Verrucomicrobia*<br>↓ *Bacteroides*, *Streptococcus*, *Subdoligranulum*	G	[437-440]

Note: A indicate Flavonoids, B indicate Terpenoids, C indicate Alkaloids, D indicate Quinonoids, E indicate Steroids, G indicate Phenylpropanoids, H indicate Saponins, I indicate Phenols, J indicate Vitamins, K indicate Other.

References

[1] Liu Y, Wu J, Chen L, Wu X, Gan Y, Xu N, Li M, Luo H, Guan F, Su Z, Chen J, Li Y, beta-patchoulene simultaneously ameliorated dextran sulfate sodium-induced colitis and secondary liver injury in mice via suppressing colonic leakage and flora imbalance, Biochem Pharmacol 182 (2020) 114260, https://doi.org/10.1016/j.bcp.2020.114260.

[2] Wu YT, Zhong LS, Huang C, Guo YY, Jin FJ, Hu YZ, Zhao ZB, Ren Z, Wang YF, beta-Caryophyllene Acts as a Ferroptosis Inhibitor to Ameliorate Experimental Colitis, International journal of molecular sciences 23 (2022) 16055, https://doi.org/10.3390/ijms232416055.

[3] Yeom JE, Kim SK, Park SY, Regulation of the Gut Microbiota and Inflammation by beta-Caryophyllene Extracted from Cloves in a Dextran Sulfate Sodium-Induced Colitis Mouse Model, Molecules 27 (2022) 7782, https://doi.org/10.3390/molecules27227782.

[4] Zhu L, Song Y, Liu H, Wu M, Gong H, Lan H, Zheng X, Gut microbiota regulation and anti-inflammatory effect of beta-carotene in dextran sulfate sodium-stimulated ulcerative colitis in rats, Journal of food science 86 (2021) 2118-2130, https://doi.org/10.1111/1750-3841.15684.

[5] Trivedi PP, Jena GB, Mechanistic insight into beta-carotene-mediated protection against ulcerative colitis-associated local and systemic damage in mice, Eur J Nutr 54 (2015) 639-652, https://doi.org/10.1007/s00394-014-0745-5.

[6] You BH, Chae HS, Song J, Ko HW, Chin YW, Choi YH, alpha-Mangostin ameliorates dextran sulfate sodium-induced colitis through inhibition of NF-kappaB and MAPK pathways, Int Immunopharmacol 49 (2017) 212-221, https://doi.org/10.1016/j.intimp.2017.05.040.

[7] Tatiya-Aphiradee N, Chatuphonprasert W, Jarukamjorn K, Ethanolic Garcinia mangostana extract and alpha-mangostin improve dextran sulfate sodium-induced ulcerative colitis via the suppression of inflammatory and oxidative responses in ICR mice, Journal of ethnopharmacology 265 (2021) 113384, https://doi.org/10.1016/j.jep.2020.113384.

[8] Gutierrez-Orozco F, Thomas-Ahner JM, Berman-Booty LD, Galley JD, Chitchumroonchokchai C, Mace T, Suksamrarn S, Bailey MT, Clinton SK, Lesinski GB, Failla ML, Dietary alpha-mangostin, a xanthone from mangosteen fruit, exacerbates experimental colitis and promotes dysbiosis in mice, Mol Nutr Food Res 58 (2014) 1226-1238, https://doi.org/10.1002/mnfr.201300771.

[9] Yamada S, Koyama T, Noguchi H, Ueda Y, Kitsuyama R, Shimizu H, Tanimoto A, Wang KY, Nawata A, Nakayama T, Sasaguri Y, Satoh T, Marine hydroquinone zonarol prevents inflammation and apoptosis in dextran sulfate sodium-induced mice ulcerative colitis, PLoS One 9 (2014) e113509, https://doi.org/10.1371/journal.pone.0113509.

[10] Zhang Z, Cui Y, Liu S, Huang J, Liu Y, Zhou Y, Zhu Z, Short-term treatment with zingerone ameliorates dextran sulfate sodium-induced mouse experimental colitis, J Sci Food Agric 102 (2022) 4873-4882, https://doi.org/10.1002/jsfa.11850.

[11] Murakami A, Hayashi R, Tanaka T, Kwon KH, Ohigashi H, Safitri R, Suppression of dextran sodium sulfate-induced colitis in mice by zerumbone, a subtropical ginger sesquiterpene, and nimesulide: separately and in combination, Biochem Pharmacol 66 (2003) 1253-1261, https://doi.org/10.1016/s0006-2952(03)00446-5.

[12] El-Akabawy G, El-Sherif NM, Zeaxanthin exerts protective effects on acetic acid-induced colitis in rats via modulation of pro-inflammatory cytokines and oxidative stress, Biomed Pharmacother 111 (2019) 841-851, https://doi.org/10.1016/j.biopha.2019.01.001.

[13] Huang S, Fu Y, Xu B, Liu C, Wang Q, Luo S, Nong F, Wang X, Huang S, Chen J, Zhou L, Luo X, Wogonoside alleviates colitis by improving intestinal epithelial barrier function via the MLCK/pMLC2 pathway, Phytomedicine 68 (2020) 153179, https://doi.org/10.1016/j.phymed.2020.153179.

[14] Zhou Y, Dou F, Song H, Liu T, Anti-ulcerative effects of wogonin on ulcerative colitis induced by dextran sulfate sodium via Nrf2/TLR4/NF-kappaB signaling pathway in BALB/c mice, Environ Toxicol 37 (2022) 954-963, https://doi.org/10.1002/tox.23457.

[15] Prakash T, Janadri S, Anti-inflammatory effect of wedelolactone on DSS induced colitis in rats: IL-6/STAT3 signaling pathway, J Ayurveda Integr Med 14 (2023) 100544, https://doi.org/10.1016/j.jaim.2022.100544.

[16] Wei W, Ding M, Zhou K, Xie H, Zhang M, Zhang C, Protective effects of wedelolactone on dextran sodium sulfate induced murine colitis partly through inhibiting the NLRP3 inflammasome activation via AMPK signaling, Biomedicine & Pharmacotherapy 94 (2017) 27-36, https://doi.org/10.1016/j.biopha.2017.06.071.

[17] Zhang J, Liang F, Chen Z, Chen Y, Yuan J, Xiong Q, Hou S, Huang S, Liu C, Liang J, Vitexin Protects against Dextran Sodium Sulfate-Induced Colitis in Mice and Its Potential Mechanisms, Journal of Agricultural and Food Chemistry 70 (2022) 12041-12054, https://doi.org/10.1021/acs.jafc.2c05177.

[18] Duan S, Du X, Chen S, Liang J, Huang S, Hou S, Gao J, Ding P, Effect of vitexin on alleviating liver inflammation in a dextran sulfate sodium (DSS)-induced colitis model, Biomedicine & pharmacotherapy 121 (2020) 109683, https://doi.org/10.1016/j.biopha.2019.109683.

[19] Tahan G, Aytac E, Aytekin H, Gunduz F, Dogusoy G, Aydin S, Tahan V, Uzun H, Vitamin E has a dual effect of anti-inflammatory and antioxidant activities in acetic acid-induced ulcerative colitis in rats, Canadian Journal of Surgery 54 (2011) 333-338, https://doi.org/10.1503/cjs.013610.

[20] Fan X, Yin J, Yin J, Weng X, Ding R, Comparison of the anti-inflammatory effects of vitamin E and vitamin D on a rat model of dextran sulfate sodium-induced ulcerative colitis, Experimental and Therapeutic Medicine 25 (2023) 98, https://doi.org/10.3892/etm.2023.11797.

[21] Lu Y, Chen J, He X, Xu S, Chen YE, Gao J, Hou S, Combined Administration of Vitamin D(3) and Geniposide Is Less Effective than Single Use of Vitamin D(3) or Geniposide in the Treatment of Ulcerative Colitis, Frontiers in Pharmacology 12 (2021) 714065, https://doi.org/10.3389/fphar.2021.714065.

[22] Gao H, Zhou H, Zhang Z, Gao J, Li J, Li X, Vitamin D3 alleviates inflammation in ulcerative colitis by activating the VDR-NLRP6 signaling pathway, Frontiers in Immunology 14 (2023) 1135930, https://doi.org/10.3389/fimmu.2023.1135930.

[23] Kondo K, Hiramoto K, Yamate Y, Goto K, Sekijima H, Ooi K, Ameliorative Effect of High-Dose Vitamin C Administration on Dextran Sulfate Sodium-Induced Colitis Mouse Model, Biological & Pharmaceutical Bulletin 42 (2019) 954-959, https://doi.org/10.1248/bpb.b18-00967.

[24] Yan HY, Wang HG, Zhang XL, Li XQ, Yu J, Ascorbic acid ameliorates oxidative stress and inflammation in dextran sulfate sodium-induced ulcerative colitis in mice, International Journal of Clinical and Experimental Medicine 8 (2015) 20245-20253,

[25] Pang B, Jin H, Liao N, Li J, Jiang C, Shi J, Vitamin A supplementation ameliorates ulcerative colitis in gut microbiota-dependent manner, Food Res Int 148 (2021) 110568, https://doi.org/10.1016/j.foodres.2021.110568.

[26] Yin Y, Ye L, Niu Z, Fang W, Anti-inflammatory effects of Vicenin-2 on dextran sulfate sodium-induced colitis in mice, Drug Dev Res 80 (2019) 546-555, https://doi.org/10.1002/ddr.21529.

[27] Sheng Q, Li F, Chen G, Li J, Li J, Wang Y, Lu Y, Li Q, Li M, Chai K, Ursolic Acid Regulates Intestinal Microbiota and Inflammatory Cell Infiltration to Prevent Ulcerative Colitis, J Immunol Res 2021 (2021) 6679316, https://doi.org/10.1155/2021/6679316.

[28] Liu B, Piao X, Guo L, Liu S, Chai F, Gao L, Ursolic acid protects against ulcerative colitis via anti-inflammatory and antioxidant effects in mice, Mol Med Rep 13 (2016) 4779-4785, https://doi.org/10.3892/mmr.2016.5094.

[29] Martínez-Moya P, Romero-Calvo I, Requena P, Hernández-Chirlaque C, Aranda CJ, González R, Zarzuelo A, Suárez MD, Martínez-Augustin O, Marín JJG, de Medina FS, Dose-dependent antiinflammatory effect of ursodeoxycholic acid in experimental colitis, International Immunopharmacology 15 (2013) 372-380, https://doi.org/10.1016/j.intimp.2012.11.017.

[30] Guvenc M, Cellat M, Ozkan H, Tekeli IO, Uyar A, Gokcek I, Isler CT, Yakan A, Protective Effects of Tyrosol Against DSS-Induced Ulcerative Colitis in Rats, Inflammation 42 (2019) 1680-1691, https://doi.org/10.1007/s10753-019-01028-8.

[31] Shizuma T, Mori H, Fukuyama N, Protective effect of tryptophan against dextran sulfate sodium- induced experimental colitis, Turk J Gastroenterol 24 (2013) 30-35,

https://doi.org/10.4318/tjg.2013.0558.

[32] Islam J, Sato S, Watanabe K, Watanabe T, Ardiansyah, Hirahara K, Aoyama Y, Tomita S, Aso H, Komai M, Shirakawa H, Dietary tryptophan alleviates dextran sodium sulfate-induced colitis through aryl hydrocarbon receptor in mice, J Nutr Biochem 42 (2017) 43-50, https://doi.org/10.1016/j.jnutbio.2016.12.019.

[33] Zhang H, Gong C, Qu L, Ding X, Cao W, Chen H, Zhang B, Zhou G, Therapeutic effects of triptolide via the inhibition of IL-1beta expression in a mouse model of ulcerative colitis, Exp Ther Med 12 (2016) 1279-1286, https://doi.org/10.3892/etm.2016.3490.

[34] Tang B, Zhu J, Zhang B, Wu F, Wang Y, Weng Q, Fang S, Zheng L, Yang Y, Qiu R, Chen M, Xu M, Zhao Z, Ji J, Therapeutic Potential of Triptolide as an Anti-Inflammatory Agent in Dextran Sulfate Sodium-Induced Murine Experimental Colitis, Front Immunol 11 (2020) 592084, https://doi.org/10.3389/fimmu.2020.592084.

[35] Zhang H, Chen W, Interleukin 6 inhibition by triptolide prevents inflammation in a mouse model of ulcerative colitis, Exp Ther Med 14 (2017) 2271-2276, https://doi.org/10.3892/etm.2017.4778.

[36] Wu H, Rao Q, Ma GC, Yu XH, Zhang CE, Ma ZJ, Effect of Triptolide on Dextran Sodium Sulfate-Induced Ulcerative Colitis and Gut Microbiota in Mice, Front Pharmacol 10 (2019) 1652, https://doi.org/10.3389/fphar.2019.01652.

[37] Zhuang H, Lv Q, Zhong C, Cui Y, He L, Zhang C, Yu J, Tiliroside Ameliorates Ulcerative Colitis by Restoring the M1/M2 Macrophage Balance via the HIF-1alpha/glycolysis Pathway, Frontiers in Immunology 12 (2021) 649463, https://doi.org/10.3389/fimmu.2021.649463.

[38] Tahmasebi P, Abtahi Froushani SM, Afzale Ahangaran N, Thymol has beneficial effects on the experimental model of ulcerative colitis, Avicenna Journal of Phytomedicine 9 (2019) 538-550, https://doi.org/10.22038/AJP.2019.13383.

[39] Chamanara M, Abdollahi A, Rezayat SM, Ghazi-Khansari M, Dehpour A, Nassireslami E, Rashidian A, Thymol reduces acetic acid-induced inflammatory response through inhibition of NF-kB signaling pathway in rat colon tissue, Inflammopharmacology 27 (2019) 1275-1283, https://doi.org/10.1007/s10787-019-00583-8.

[40] Ghasemi-Pirbaluti M, Motaghi E, Najafi A, Hosseini MJ, The effect of theophylline on acetic acid induced ulcerative colitis in rats, Biomed Pharmacother 90 (2017) 153-159, https://doi.org/10.1016/j.biopha.2017.03.038.

[41] Zhang DK, Cheng LN, Huang XL, Shi W, Xiang JY, Gan HT, Tetrandrine ameliorates dextran-sulfate-sodium-induced colitis in mice through inhibition of nuclear factor -kappaB activation, Int J Colorectal Dis 24 (2009) 5-12, https://doi.org/10.1007/s00384-008-0544-7.

[42] He X, Zheng Z, Yang X, Lu Y, Chen N, Chen W, Tetramethylpyrazine attenuates PPAR-gamma antagonist-deteriorated oxazolone-induced colitis in mice, Mol Med Rep 5 (2012) 645-650, https://doi.org/10.3892/mmr.2011.721.

[43] Zhang Z, Shen P, Lu X, Li Y, Liu J, Liu B, Fu Y, Cao Y, Zhang N, In Vivo and In Vitro Study on the Efficacy of Terpinen-4-ol in Dextran Sulfate Sodium-Induced Mice Experimental Colitis, Front Immunol 8 (2017) 558, https://doi.org/10.3389/fimmu.2017.00558.

[44] Li W, Zhang L, Xu Q, Yang W, Zhao J, Ren Y, Yu Z, Ma L, Taxifolin Alleviates DSS-Induced Ulcerative Colitis by Acting on Gut Microbiome to Produce Butyric Acid,

Nutrients 14 (2022) 1069, https://doi.org/10.3390/nu14051069.
[45] Yang Y, He J, Suo Y, Zheng Z, Wang J, Lv L, Huo C, Wang Z, Li J, Sun W, Zhang Y, Tauroursodeoxycholate improves 2,4,6-trinitrobenzenesulfonic acid-induced experimental acute ulcerative colitis in mice, Int Immunopharmacol 36 (2016) 271-276, https://doi.org/10.1016/j.intimp.2016.04.037.
[46] Lv L, Chen Z, Bai W, Hao J, Heng Z, Meng C, Wang L, Luo X, Wang X, Cao Y, He J, Taurohyodeoxycholic acid alleviates trinitrobenzene sulfonic acid induced ulcerative colitis via regulating Th1/Th2 and Th17/Treg cells balance, Life Sci 318 (2023) 121501, https://doi.org/10.1016/j.lfs.2023.121501.
[47] He J, Liang J, Zhu S, Zhao W, Zhang Y, Sun W, Protective effect of taurohyodeoxycholic acid from Pulvis Fellis Suis on trinitrobenzene sulfonic acid induced ulcerative colitis in mice, Eur J Pharmacol 670 (2011) 229-235, https://doi.org/10.1016/j.ejphar.2011.08.036.
[48] Laukens D, Devisscher L, Van den Bossche L, Hindryckx P, Vandenbroucke RE, Vandewynckel YP, Cuvelier C, Brinkman BM, Libert C, Vandenabeele P, De Vos M, Tauroursodeoxycholic acid inhibits experimental colitis by preventing early intestinal epithelial cell death, Lab Invest 94 (2014) 1419-1430, https://doi.org/10.1038/labinvest.2014.117.
[49] Yang Y, He J, Suo Y, Lv L, Wang J, Huo C, Zheng Z, Wang Z, Li J, Sun W, Zhang Y, Anti-inflammatory effect of taurocholate on TNBS-induced ulcerative colitis in mice, Biomed Pharmacother 81 (2016) 424-430, https://doi.org/10.1016/j.biopha.2016.04.037.
[50] Giris M, Depboylu B, Dogru-Abbasoglu S, Erbil Y, Olgac V, Alis H, Aykac-Toker G, Uysal M, Effect of taurine on oxidative stress and apoptosis-related protein expression in trinitrobenzene sulphonic acid-induced colitis, Clin Exp Immunol 152 (2008) 102-110, https://doi.org/10.1111/j.1365-2249.2008.03599.x.
[51] Che L, Li Y, Song R, Qin C, Hao W, Wang B, Yang L, Peng P, Xu F, Anti-inflammatory and anti-apoptosis activity of taraxasterol in ulcerative colitis in vitro and in vivo, Exp Ther Med 18 (2019) 1745-1751, https://doi.org/10.3892/etm.2019.7736.
[52] Fang W, Zhu S, Niu Z, Yin Y, The protective effect of syringic acid on dextran sulfate sodium-induced experimental colitis in BALB/c mice, Drug Dev Res 80 (2019) 731-740, https://doi.org/10.1002/ddr.21524.
[53] Ekhtiar M, Ghasemi-Dehnoo M, Mirzaei Y, Azadegan-Dehkordi F, Amini-Khoei H, Lorigooini Z, Samiei-Sefat A, Bagheri N, The coumaric acid and syringic acid ameliorate acetic acid-induced ulcerative colitis in rats via modulator of Nrf2/HO-1 and pro-inflammatory cytokines, Int Immunopharmacol 120 (2023) 110309, https://doi.org/10.1016/j.intimp.2023.110309.
[54] He C, Gao M, Zhang X, Lei P, Yang H, Qing Y, Zhang L, The Protective Effect of Sulforaphane on Dextran Sulfate Sodium-Induced Colitis Depends on Gut Microbial and Nrf2-Related Mechanism, Front Nutr 9 (2022) 893344, https://doi.org/10.3389/fnut.2022.893344.
[55] Zhang Y, Tan L, Li C, Wu H, Ran D, Zhang Z, Sulforaphane alter the microbiota and mitigate colitis severity on mice ulcerative colitis induced by DSS, AMB Express 10 (2020) 119, https://doi.org/10.1186/s13568-020-01053-z.
[56] Mostafa AF, Elalfy MM, Shata A, Elhadidy MG, Prophylactic effect of aquatic extract of stevia on acetic acid induced-ulcerative colitis in male rats: a possible role of

Nrf2 and PPARgamma, J Basic Clin Physiol Pharmacol 32 (2020) 1093-1104, https://doi.org/10.1515/jbcpp-2020-0039.
[57] Alavala S, Sangaraju R, Nalban N, Sahu BD, Jerald MK, Kilari EK, Sistla R, Stevioside, a diterpenoid glycoside, shows anti-inflammatory property against Dextran Sulphate Sodium-induced ulcerative colitis in mice, Eur J Pharmacol 855 (2019) 192-201, https://doi.org/10.1016/j.ejphar.2019.05.015.
[58] Sanchez-Fidalgo S, Villegas I, Rosillo MA, Aparicio-Soto M, de la Lastra CA, Dietary squalene supplementation improves DSS-induced acute colitis by downregulating p38 MAPK and NFkB signaling pathways, Mol Nutr Food Res 59 (2015) 284-292, https://doi.org/10.1002/mnfr.201400518.
[59] Medicherla K, Sahu BD, Kuncha M, Kumar JM, Sudhakar G, Sistla R, Oral administration of geraniol ameliorates acute experimental murine colitis by inhibiting pro-inflammatory cytokines and NF-kappaB signaling, Food Funct 6 (2015) 2984-2995, https://doi.org/10.1039/c5fo00405e.
[60] Cheng T, Xu C, Wu D, Yan G, Wang C, Wang T, Shao J, Sodium houttuyfonate derived from Houttuynia cordata Thunb improves intestinal malfunction via maintaining gut microflora stability in Candida albicans overgrowth aggravated ulcerative colitis, Food Funct 14 (2023) 1072-1086, https://doi.org/10.1039/d2fo02369e.
[61] Ni L, Jing S, Zhu L, Yang X, Wang X, Tu S, The Immune Change of the Lung and Bowel in an Ulcerative Colitis Rat Model and the Protective Effect of Sodium Houttuyfonate Combined With Matrine, Front Immunol 13 (2022) 888918, https://doi.org/10.3389/fimmu.2022.888918.
[62] Zhang M, Long Y, Sun Y, Wang Y, Li Q, Wu H, Guo Z, Li Y, Niu Y, Li C, Liu L, Mei Q, Evidence for the complementary and synergistic effects of the three-alkaloid combination regimen containing berberine, hypaconitine and skimmianine on the ulcerative colitis rats induced by trinitrobenzene-sulfonic acid, Eur J Pharmacol 651 (2011) 187-196, https://doi.org/10.1016/j.ejphar.2010.10.030.
[63] Zhou Y, Chen S, Gu W, Sun X, Wang L, Tang L, Sinomenine hydrochloride ameliorates dextran sulfate sodium-induced colitis in mice by modulating the gut microbiota composition whilst suppressing the activation of the NLRP3 inflammasome, Exp Ther Med 22 (2021) 1287, https://doi.org/10.3892/etm.2021.10722.
[64] Zhou Y, Chen S, Dai Y, Wu L, Jin M, Zhao J, Li Y, Tang L, Sinomenine attenuated dextran sulfate sodium-induced inflammatory responses by promoting 14-3-3theta protein and inhibiting NF-kappaB signaling, J Ethnopharmacol 303 (2023) 116037, https://doi.org/10.1016/j.jep.2022.116037.
[65] Zhou Y, Liu H, Song J, Cao L, Tang L, Qi C, Sinomenine alleviates dextran sulfate sodium-induced colitis via the Nrf2/NQO-1 signaling pathway, Mol Med Rep 18 (2018) 3691-3698, https://doi.org/10.3892/mmr.2018.9378.
[66] Kotipalli RSS, Tirunavalli SK, Pote AB, Sahu BD, Kuncha M, Jerald MK, Sistla R, Andugulapati SB, Sinigrin Attenuates the Dextran Sulfate Sodium-induced Colitis in Mice by Modulating the MAPK Pathway, Inflammation 46 (2023) 787-807, https://doi.org/10.1007/s10753-022-01780-4.
[67] Qian B, Wang C, Zeng Z, Ren Y, Li D, Song JL, Ameliorative Effect of Sinapic Acid on Dextran Sodium Sulfate- (DSS-) Induced Ulcerative Colitis in Kunming (KM) Mice, Oxid Med Cell Longev 2020 (2020) 8393504, https://doi.org/10.1155/2020/8393504.
[68] Shahid M, Raish M, Ahmad A, Bin Jardan YA, Ansari MA, Ahad A, Alkharfy KM, Alaofi AL, Al-Jenoobi FI, Sinapic Acid Ameliorates Acetic Acid-Induced Ulcerative Colitis in Rats by Suppressing Inflammation, Oxidative Stress, and Apoptosis, Molecules 27 (2022) 4139, https://doi.org/10.3390/molecules27134139.

[69] Huang B, Wang Q, Jiang L, Lu S, Li C, Xu C, Wang C, Zhang E, Zhang X, Shikonin ameliorated mice colitis by inhibiting dimerization and tetramerization of PKM2 in macrophages, Front Pharmacol 13 (2022) 926945, https://doi.org/10.3389/fphar.2022.926945.
[70] Bai X, Gou X, Cai P, Xu C, Cao L, Zhao Z, Huang M, Jin J, Sesamin Enhances Nrf2-Mediated Protective Defense against Oxidative Stress and Inflammation in Colitis via AKT and ERK Activation, Oxid Med Cell Longev 2019 (2019) 2432416, https://doi.org/10.1155/2019/2432416.
[71] Zhang H, Hua R, Zhang B, Zhang X, Yang H, Zhou X, Serine Alleviates Dextran Sulfate Sodium-Induced Colitis and Regulates the Gut Microbiota in Mice, Front Microbiol 9 (2018) 3062, https://doi.org/10.3389/fmicb.2018.03062.
[72] Lifei L, Zhang J, Li X, Zhu Y, Wang Y, Liu D, Sericic Acid Ameliorates DSS-induced Ulcerative Colitis in Mice by Modulating the NF-kappaB and Nrf2 Pathways, Curr Mol Pharmacol 16 (2023) 759-770, https://doi.org/10.2174/1874467215666220928100319.
[73] Li J, Lu Y, Wang D, Quan F, Chen X, Sun R, Zhao S, Yang Z, Tao W, Ding D, Gao X, Cao Q, Zhao D, Qi R, Chen C, He L, Hu K, Chen Z, Yang Y, Luo Y, Schisandrin B prevents ulcerative colitis and colitis-associated-cancer by activating focal adhesion kinase and influence on gut microbiota in an in vivo and in vitro model, Eur J Pharmacol 854 (2019) 9-21, https://doi.org/10.1016/j.ejphar.2019.03.059.
[74] Zhang W, Wang W, Shen C, Wang X, Pu Z, Yin Q, Network pharmacology for systematic understanding of Schisandrin B reduces the epithelial cells injury of colitis through regulating pyroptosis by AMPK/Nrf2/NLRP3 inflammasome, Aging-Us 13 (2021) 23193-23209,
[75] Wu K, Liu X, Meng X, Cao L, Li H, Bi Y, Wang M, Wang M, Jiang Y, Sauchinone alleviates dextran sulfate sodium-induced ulcerative colitis via NAD(P)H dehydrogenase [quinone] 1/NF-kB pathway and gut microbiota, Front Microbiol 13 (2022) 1084257, https://doi.org/10.3389/fmicb.2022.1084257.
[76] Mandlik DS, Mandlik SK, Patel S, Protective effect of sarsasapogenin in TNBS induced ulcerative colitis in rats associated with downregulation of pro-inflammatory mediators and oxidative stress, Immunopharmacol Immunotoxicol 43 (2021) 571-583, https://doi.org/10.1080/08923973.2021.1955919.
[77] Li X, Wu X, Wang Q, Xu W, Zhao Q, Xu N, Hu X, Ye Z, Yu S, Liu J, He X, Shi F, Zhang Q, Li W, Sanguinarine ameliorates DSS induced ulcerative colitis by inhibiting NLRP3 inflammasome activation and modulating intestinal microbiota in C57BL/6 mice, Phytomedicine 104 (2022) 154321, https://doi.org/10.1016/j.phymed.2022.154321.
[78] Niu X, Fan T, Li W, Huang H, Zhang Y, Xing W, Protective effect of sanguinarine against acetic acid-induced ulcerative colitis in mice, Toxicol Appl Pharmacol 267 (2013) 256-265, https://doi.org/10.1016/j.taap.2013.01.009.
[79] Liu J, Cai J, Fan P, Zhang N, Cao Y, The Abilities of Salidroside on Ameliorating Inflammation, Skewing the Imbalanced Nucleotide Oligomerization Domain-Like Receptor Family Pyrin Domain Containing 3/Autophagy, and Maintaining Intestinal Barrier Are Profitable in Colitis, Front Pharmacol 10 (2019) 1385, https://doi.org/10.3389/fphar.2019.01385.
[80] Liu X, Zhou M, Dai Z, Luo S, Shi Y, He Z, Chen Y, Salidroside alleviates ulcerative colitis via inhibiting macrophage pyroptosis and repairing the dysbacteriosis-associated Th17/Treg imbalance, Phytother Res 37 (2023) 367-382, https://doi.org/10.1002/ptr.7636.

[81] Li P, Wu M, Xiong W, Li J, An Y, Ren J, Xie Y, Xue H, Yan D, Li M, Zhong G, Saikosaponin-d ameliorates dextran sulfate sodium-induced colitis by suppressing NF-kappaB activation and modulating the gut microbiota in mice, Int Immunopharmacol 81 (2020) 106288, https://doi.org/10.1016/j.intimp.2020.106288.
[82] Zhou F, Wang N, Yang L, Zhang LC, Meng LJ, Xia YC, Saikosaponin A protects against dextran sulfate sodium-induced colitis in mice, Int Immunopharmacol 72 (2019) 454-458, https://doi.org/10.1016/j.intimp.2019.04.024.
[83] Formiga RO, Alves Junior EB, Vasconcelos RC, Guerra GCB, Antunes de Araujo A, Carvalho TG, Garcia VB, de Araujo Junior RF, Gadelha F, Vieira GC, Sobral MV, Barbosa Filho JM, Spiller F, Batista LM, p-Cymene and Rosmarinic Acid Ameliorate TNBS-Induced Intestinal Inflammation Upkeeping ZO-1 and MUC-2: Role of Antioxidant System and Immunomodulation, Int J Mol Sci 21 (2020) 5870, https://doi.org/10.3390/ijms21165870.
[84] Jin BR, Chung KS, Cheon SY, Lee M, Hwang S, Noh Hwang S, Rhee KJ, An HJ, Rosmarinic acid suppresses colonic inflammation in dextran sulphate sodium (DSS)-induced mice via dual inhibition of NF-kappaB and STAT3 activation, Sci Rep 7 (2017) 46252, https://doi.org/10.1038/srep46252.
[85] Karakoyun B, Ertas B, Yuksel M, Akakin D, Cevik O, Sener G, Ameliorative effects of riboflavin on acetic acid-induced colonic injury in rats, Clin Exp Pharmacol Physiol 45 (2018) 563-572, https://doi.org/10.1111/1440-1681.12894.
[86] Gao F, Zhong HY, Chen KX, Dong LL, Lin MS, Du HL, Mechanism of combined treatment of rhein and emodin in Rhubarb for ulcerative colitis, China J. Chin. Mater. Med. 47 (2022) 4148-4155, https://doi.org/10.19540/j.cnki.cjcmm.20220509.702.
[87] Dong L, Du H, Zhang M, Xu H, Pu X, Chen Q, Luo R, Hu Y, Wang Y, Tu H, Zhang J, Gao F, Anti-inflammatory effect of Rhein on ulcerative colitis via inhibiting PI3K/Akt/mTOR signaling pathway and regulating gut microbiota, Phytother Res 36 (2022) 2081-2094, https://doi.org/10.1002/ptr.7429.
[88] Zhu F, Zheng J, Xu F, Xi Y, Chen J, Xu X, Resveratrol Alleviates Dextran Sulfate Sodium-Induced Acute Ulcerative Colitis in Mice by Mediating PI3K/Akt/VEGFA Pathway, Front Pharmacol 12 (2021) 693982, https://doi.org/10.3389/fphar.2021.693982.
[89] Zhang L, Xue H, Zhao G, Qiao C, Sun X, Pang C, Zhang D, Curcumin and resveratrol suppress dextran sulfate sodium-induced colitis in mice, Mol Med Rep 19 (2019) 3053-3060, https://doi.org/10.3892/mmr.2019.9974.
[90] Cui X, Jin Y, Hofseth AB, Pena E, Habiger J, Chumanevich A, Poudyal D, Nagarkatti M, Nagarkatti PS, Singh UP, Hofseth LJ, Resveratrol suppresses colitis and colon cancer associated with colitis, Cancer Prev Res (Phila) 3 (2010) 549-559, https://doi.org/10.1158/1940-6207.CAPR-09-0117.
[91] Pan HH, Zhou XX, Ma YY, Pan WS, Zhao F, Yu MS, Liu JQ, Resveratrol alleviates intestinal mucosal barrier dysfunction in dextran sulfate sodium-induced colitis mice by enhancing autophagy, World Journal of Gastroenterology 26 (2020) 16, https://doi.org/10.3748/wjg.v26.i33.4945.
[92] Abdallah DM, Ismael NR, Resveratrol abrogates adhesion molecules and protects against TNBS-induced ulcerative colitis in rats, Canadian Journal of Physiology and Pharmacology 89 (2011) 811-818, https://doi.org/10.1139/y11-080.
[93] Yao J, Wang JY, Liu L, Li YX, Xun AY, Zeng WS, Jia CH, Wei XX, Feng JL, Zhao L, Wang LS, Anti-oxidant effects of resveratrol on mice with DSS-induced ulcerative

colitis, Arch Med Res 41 (2010) 288-294, https://doi.org/10.1016/j.arcmed.2010.05.002.

[94] Liu X, Wu YL, Liu KL, Cui XL, Du XX, Zhang WQ, Effects of resveratrol on ulcerative colitis in mice and its mechanism, Chinese Journal of Applied Physiology 35 (2019) 447-453,

[95] Yao J, Wei C, Wang JY, Zhang R, Li YX, Wang LS, Effect of resveratrol on Treg/Th17 signaling and ulcerative colitis treatment in mice, World J Gastroenterol 21 (2015) 6572-6581, https://doi.org/10.3748/wjg.v21.i21.6572.

[96] Abdin AA, Targeting sphingosine kinase 1 (SphK1) and apoptosis by colon-specific delivery formula of resveratrol in treatment of experimental ulcerative colitis in rats, Eur J Pharmacol 718 (2013) 145-153, https://doi.org/10.1016/j.ejphar.2013.08.040.

[97] Sanchez-Fidalgo S, Cardeno A, Villegas I, Talero E, de la Lastra CA, Dietary supplementation of resveratrol attenuates chronic colonic inflammation in mice, Eur J Pharmacol 633 (2010) 78-84, https://doi.org/10.1016/j.ejphar.2010.01.025.

[98] Donder Y, Arikan TB, Baykan M, Akyuz M, Oz AB, Effects of quercitrin on bacterial translocation in a rat model of experimental colitis, Asian Journal of Surgery 41 (2018) 543-550, https://doi.org/10.1016/j.asjsur.2017.12.002.

[99] Zhang Q, Wen F, Sun F, Xu Z, Liu Y, Tao C, Sun F, Jiang M, Yang M, Yao J, Efficacy and Mechanism of Quercetin in the Treatment of Experimental Colitis Using Network Pharmacology Analysis, Molecules 28 (2022) 146, https://doi.org/10.3390/molecules28010146.

[100] Kottakis G, Kambouri K, Giatromanolaki A, Valsami G, Kostomitsopoulos N, Tsaroucha A, Pitiakoudis M, Effects of the Antioxidant Quercetin in an Experimental Model of Ulcerative Colitis in Mice, Medicina (Kaunas) 59 (2022) 87, https://doi.org/10.3390/medicina59010087.

[101] Yin Z, Wang Q, Cheng H, Synergistic Protective Effect of Interactions of Quercetin with Lycopene Against Ochratoxin A-Induced Ulcerative Colitis, Appl Biochem Biotechnol 195 (2023) 5253-5266, https://doi.org/10.1007/s12010-022-04287-8.

[102] Wu Y, Li Y, Ruan Z, Li J, Zhang L, Lu H, Xu Z, Puerarin Rebuilding the Mucus Layer and Regulating Mucin-Utilizing Bacteria to Relieve Ulcerative Colitis, J Agric Food Chem 68 (2020) 11402-11411, https://doi.org/10.1021/acs.jafc.0c04119.

[103] Jeon YD, Lee JH, Lee YM, Kim DK, Puerarin inhibits inflammation and oxidative stress in dextran sulfate sodium-induced colitis mice model, Biomed Pharmacother 124 (2020) 109847, https://doi.org/10.1016/j.biopha.2020.109847.

[104] Zhang H, Lang W, Liu X, Bai J, Jia Q, Shi Q, Procyanidin A1 alleviates DSS-induced ulcerative colitis via regulating AMPK/mTOR/p70S6K-mediated autophagy, J Physiol Biochem 78 (2022) 213-227, https://doi.org/10.1007/s13105-021-00854-5.

[105] Yin Y, Liu K, Li G, Protective Effect of Prim-O-Glucosylcimifugin on Ulcerative Colitis and Its Mechanism, Front Pharmacol 13 (2022) 882924, https://doi.org/10.3389/fphar.2022.882924.

[106] Peritore AF, D'Amico R, Cordaro M, Siracusa R, Fusco R, Gugliandolo E, Genovese T, Crupi R, Di Paola R, Cuzzocrea S, Impellizzeri D, PEA/Polydatin: Anti-

Inflammatory and Antioxidant Approach to Counteract DNBS-Induced Colitis, Antioxidants (Basel) 10 (2021) 464, https://doi.org/10.3390/antiox10030464.
[107] Yao J, Wang JY, Liu L, Zeng WS, Li YX, Xun AY, Zhao L, Jia CH, Feng JL, Wei XX, Wang LS, Polydatin ameliorates DSS-induced colitis in mice through inhibition of nuclear factor-kappaB activation, Planta Med 77 (2011) 421-427, https://doi.org/10.1055/s-0030-1250462.
[108] Lv T, Shen L, Yang L, Diao W, Yang Z, Zhang Y, Yu S, Li Y, Polydatin ameliorates dextran sulfate sodium-induced colitis by decreasing oxidative stress and apoptosis partially via Sonic hedgehog signaling pathway, Int Immunopharmacol 64 (2018) 256-263, https://doi.org/10.1016/j.intimp.2018.09.009.
[109] Rapa S, Di Paola R, Cordaro M, Siracusa R, D'Amico R, Fusco R, Autore G, Cuzzocrea S, Stuppner H, Marzocco S, Plumericin Protects against Experimental Inflammatory Bowel Disease by Restoring Intestinal Barrier Function and Reducing Apoptosis, Biomedicines 9 (2021) 67, https://doi.org/10.3390/biomedicines9010067.
[110] Guo R, Meng Q, Wang B, Li F, Anti-inflammatory effects of Platycodin D on dextran sulfate sodium (DSS) induced colitis and E. coli Lipopolysaccharide (LPS) induced inflammation, Int Immunopharmacol 94 (2021) 107474, https://doi.org/10.1016/j.intimp.2021.107474.
[111]Guo G, Shi F, Zhu J, Shao Y, Gong W, Zhou G, Wu H, She J, Shi W, Piperine, a functional food alkaloid, exhibits inhibitory potential against TNBS-induced colitis via the inhibition of IkappaB-alpha/NF-kappaB and induces tight junction protein (claudin-1, occludin, and ZO-1) signaling pathway in experimental mice, Hum Exp Toxicol 39 (2020) 477-491, https://doi.org/10.1177/0960327119892042.
[112] Gupta RA, Motiwala MN, Dumore NG, Danao KR, Ganjare AB, Effect of piperine on inhibition of FFA induced TLR4 mediated inflammation and amelioration of acetic acid induced ulcerative colitis in mice, J Ethnopharmacol 164 (2015) 239-246, https://doi.org/10.1016/j.jep.2015.01.039.
[113] Hu L, Wu C, Zhang Z, Liu M, Maruthi Prasad E, Chen Y, Wang K, Pinocembrin Protects Against Dextran Sulfate Sodium-Induced Rats Colitis by Ameliorating Inflammation, Improving Barrier Function and Modulating Gut Microbiota, Front Physiol 10 (2019) 908, https://doi.org/10.3389/fphys.2019.00908.
[114] Yue B, Ren J, Yu Z, Luo X, Ren Y, Zhang J, Mani S, Wang Z, Dou W, Pinocembrin alleviates ulcerative colitis in mice via regulating gut microbiota, suppressing TLR4/MD2/NF-kappaB pathway and promoting intestinal barrier, Biosci Rep 40 (2020) BSR20200986, https://doi.org/10.1042/BSR20200986.
[115] Yao H, Yan J, Yin L, Chen W, Picroside II alleviates DSS-induced ulcerative colitis by suppressing the production of NLRP3 inflammasomes through NF-kappaB signaling pathway, Immunopharmacol Immunotoxicol 44 (2022) 437-446, https://doi.org/10.1080/08923973.2022.2054425.
[116] Hou X, Sang Y, Dong L, The improved effect and its mechanism of phytic acid on DSS-induced UC mice, Life Sci 311 (2022) 121139, https://doi.org/10.1016/j.lfs.2022.121139.
[117] Zhang Q, Xu N, Hu X, Zheng Y, Anti-colitic effects of Physalin B on dextran sodium sulfate-induced BALB/c mice by suppressing multiple inflammatory signaling pathways, J Ethnopharmacol 259 (2020) 112956, https://doi.org/10.1016/j.jep.2020.112956.
[118] Zhang Z, Li S, Cao H, Shen P, Liu J, Fu Y, Cao Y, Zhang N, The protective role of phloretin against dextran sulfate sodium-induced ulcerative colitis in mice, Food Funct 10 (2019) 422-431, https://doi.org/10.1039/c8fo01699b.

[119] Wu M, Li P, An Y, Ren J, Yan D, Cui J, Li D, Li M, Wang M, Zhong G, Phloretin ameliorates dextran sulfate sodium-induced ulcerative colitis in mice by regulating the gut microbiota, Pharmacol Res 150 (2019) 104489, https://doi.org/10.1016/j.phrs.2019.104489.

[120] V SA, S KK, Phloretin Ameliorates Acetic Acid Induced Colitis Through Modulation of Immune and Inflammatory Reactions in Rats, Endocr Metab Immune Disord Drug Targets 21 (2021) 163-172, https://doi.org/10.2174/1871530320666200624120257.

[121] Xue HH, Li JJ, Li SF, Guo J, Yan RP, Chen TG, Shi XH, Wang JD, Zhang LW, Phillygenin Attenuated Colon Inflammation and Improved Intestinal Mucosal Barrier in DSS-induced Colitis Mice via TLR4/Src Mediated MAPK and NF-kappaB Signaling Pathways, Int J Mol Sci 24 (2023) 2238, https://doi.org/10.3390/ijms24032238.

[122] Su S, Wang X, Xi X, Zhu L, Chen Q, Zhang H, Qin Y, Yang B, Che N, Cao H, Zhong W, Wang B, Phellodendrine promotes autophagy by regulating the AMPK/mTOR pathway and treats ulcerative colitis, J Cell Mol Med 25 (2021) 5707-5720, https://doi.org/10.1111/jcmm.16587.

[123] Puppala ER, Aochenlar SL, Shantanu PA, Ahmed S, Jannu AK, Jala A, Yalamarthi SS, Borkar RM, Tripathi DM, Naidu VGM, Perillyl alcohol attenuates chronic restraint stress aggravated dextran sulfate sodium-induced ulcerative colitis by modulating TLR4/NF-kappaB and JAK2/STAT3 signaling pathways, Phytomedicine 106 (2022) 154415, https://doi.org/10.1016/j.phymed.2022.154415.

[124] Liu K, Li G, Guo W, Zhang J, The protective effect and mechanism of pedunculoside on DSS (dextran sulfate sodium) induced ulcerative colitis in mice, Int Immunopharmacol 88 (2020) 107017, https://doi.org/10.1016/j.intimp.2020.107017.

[125] Wu Z, Zeng H, Zhang L, Pu Y, Li S, Yuan Y, Zhang T, Wang B, Patchouli Alcohol: a Natural Sesquiterpene Against Both Inflammation and Intestinal Barrier Damage of Ulcerative Colitis, Inflammation 43 (2020) 1423-1435, https://doi.org/10.1007/s10753-020-01219-8.

[126] Qu C, Yuan ZW, Yu XT, Huang YF, Yang GH, Chen JN, Lai XP, Su ZR, Zeng HF, Xie Y, Zhang XJ, Patchouli alcohol ameliorates dextran sodium sulfate-induced experimental colitis and suppresses tryptophan catabolism, Pharmacol Res 121 (2017) 70-82, https://doi.org/10.1016/j.phrs.2017.04.017.

[127] Zhao ZJ, Xiang JY, Liu L, Huang XL, Gan HT, Parthenolide, an inhibitor of the nuclear factor-kappaB pathway, ameliorates dextran sulfate sodium-induced colitis in mice, Int Immunopharmacol 12 (2012) 169-174, https://doi.org/10.1016/j.intimp.2011.11.007.

[128] Chaparala A, Poudyal D, Tashkandi H, Witalison EE, Chumanevich AA, Hofseth JL, Nguyen I, Hardy O, Pittman DL, Wyatt MD, Windust A, Murphy EA, Nagarkatti M, Nagarkatti P, Hofseth LJ, Panaxynol, a bioactive component of American ginseng, targets macrophages and suppresses colitis in mice, Oncotarget 11 (2020) 2026-2036, https://doi.org/10.18632/oncotarget.27592.

[129] Sarnelli G, D'Alessandro A, Iuvone T, Capoccia E, Gigli S, Pesce M, Seguella L, Nobile N, Aprea G, Maione F, de Palma GD, Cuomo R, Steardo L, Esposito G, Palmitoylethanolamide Modulates Inflammation-Associated Vascular Endothelial Growth Factor (VEGF) Signaling via the Akt/mTOR Pathway in a Selective Peroxisome Proliferator-Activated Receptor Alpha (PPAR-alpha)-Dependent Manner, PLoS One 11 (2016) e0156198, https://doi.org/10.1371/journal.pone.0156198.

[130] Mai CT, Wu MM, Wang CL, Su ZR, Cheng YY, Zhang XJ, Palmatine attenuated dextran sulfate sodium (DSS)-induced colitis via promoting mitophagy-mediated

NLRP3 inflammasome inactivation, Mol Immunol 105 (2019) 76-85, https://doi.org/10.1016/j.molimm.2018.10.015.
[131] Zhang XJ, Yuan ZW, Qu C, Yu XT, Huang T, Chen PV, Su ZR, Dou YX, Wu JZ, Zeng HF, Xie Y, Chen JN, Palmatine ameliorated murine colitis by suppressing tryptophan metabolism and regulating gut microbiota, Pharmacol Res 137 (2018) 34-46, https://doi.org/10.1016/j.phrs.2018.09.010.
[132] Zheng J, Li H, Zhang P, Yue S, Zhai B, Zou J, Cheng J, Zhao C, Guo D, Wang J, Paeonol Ameliorates Ulcerative Colitis in Mice by Modulating the Gut Microbiota and Metabolites, Metabolites 12 (2022) 956, https://doi.org/10.3390/metabo12100956.
[133] Ge Y, Pan M, Zhang C, Wang C, Ma K, Yan G, Wang T, Wu D, Shao J, Paeonol alleviates dextran sodium sulfate induced colitis involving Candida albicans-associated dysbiosis, Med Mycol 59 (2021) 335-344, https://doi.org/10.1093/mmy/myaa053.
[134] Li J, Ren S, Li M, Bi J, Yang G, Li E, Paeoniflorin protects against dextran sulfate sodium (DSS)-induced colitis in mice through inhibition of inflammation and eosinophil infiltration, Int Immunopharmacol 97 (2021) 107667, https://doi.org/10.1016/j.intimp.2021.107667.
[135] Zheng K, Jia J, Yan SH, Shen H, Zhu P, Yu JY, Paeoniflorin ameliorates ulcerative colitis by modulating the dendritic cell-mediated T(H)17/T(reg)balance, Inflammopharmacology 28 (2020) 1705-1716, https://doi.org/10.1007/s10787-020-00722-6.
[136] Luo X, Wang X, Huang S, Xu B, Luo S, Li Y, Wang Q, Chen Y, Deng X, Liu L, Zhou L, Paeoniflorin ameliorates experimental colitis by inhibiting gram-positive bacteria-dependent MDP-NOD2 pathway, Int Immunopharmacol 90 (2021) 107224, https://doi.org/10.1016/j.intimp.2020.107224.
[137] Wang XJ, Luo X, Zhao ZZ, Luo S, Chen JY, Zhou L, Paeoniflorin attenuates dextran sulfate sodium-induced ulcerative colitis in mice by inhibiting TLＲ5 expression and T cell activation, Chinese Journal of Cellular and Molecular Immunology 36 (2020) 673-679, https://doi.org/10.13423/j.cnki.cjcmi.009043.
[138] Gu P, Zhu L, Liu Y, Zhang L, Liu J, Shen H, Protective effects of paeoniflorin on TNBS-induced ulcerative colitis through inhibiting NF-kappaB pathway and apoptosis in mice, Int Immunopharmacol 50 (2017) 152-160, https://doi.org/10.1016/j.intimp.2017.06.022.
[139] Miao ST, Lu QS, Zhou YJ, Chang YN, Xu T, Zhu MY, Oral administration of octacosanol modulates the gut bacteria and protects the intestinal barrier in ulcerative colitis mice, J Food Biochem 46 (2022) e14284, https://doi.org/10.1111/jfbc.14284.
[140] Lv Q, Qiao SM, Xia Y, Shi C, Xia YF, Chou GX, Wang ZT, Dai Y, Wei ZF, Norisoboldine ameliorates DSS-induced ulcerative colitis in mice through induction of regulatory T cells in colons, Int Immunopharmacol 29 (2015) 787-797, https://doi.org/10.1016/j.intimp.2015.08.040.
[141] Gao XJ, Tang B, Liang HH, Yi L, Wei ZG, The protective effect of nigeglanine on dextran sulfate sodium-induced experimental colitis in mice and Caco-2 cells, J Cell Physiol 234 (2019) 23398-23408, https://doi.org/10.1002/jcp.28909.
[142] Liu F, Yao Y, Wang Q, Zhang F, Wang M, Zhu C, Lin C, Nigakinone alleviates DSS-induced experimental colitis via regulating bile acid profile and FXR/NLRP3 signaling pathways, Phytother Res 37 (2023) 15-34, https://doi.org/10.1002/ptr.7588.
[143] Liu JF, Shao M, Zhai DW, Liu K, Wu LJ, Protective Effect of 4-Methoxy-5-hydroxycanthin-6-one, A Natural Alkaloid, on Dextran Sulfate Sodium Induced Rat

Colitis, Planta medica 75 (2009) 142–145, https://doi.org/10.1055/s-0028-1088390.
[144] Hayashi S, Hamada T, Zaidi SF, Oshiro M, Lee J, Yamamoto T, Ishii Y, Sasahara M, Kadowaki M, Nicotine suppresses acute colitis and colonic tumorigenesis associated with chronic colitis in mice, Am J Physiol Gastrointest Liver Physiol 307 (2014) G968-978, https://doi.org/10.1152/ajpgi.00346.2013.
[145] Qin Z, Wan JJ, Sun Y, Wu T, Wang PY, Du P, Su DF, Yang Y, Liu X, Nicotine protects against DSS colitis through regulating microRNA-124 and STAT3, J Mol Med (Berl) 95 (2017) 221-233, https://doi.org/10.1007/s00109-016-1473-5.
[146] Nakajima A, Shibuya T, Sasaki T, Lu YJ, Ishikawa D, Haga K, Takahashi M, Kaga N, Osada T, Sato N, Nagahara A, Nicotine Oral Administration Attenuates DSS-Induced Colitis Through Upregulation of Indole in the Distal Colon and Rectum in Mice, Front Med (Lausanne) 8 (2021) 789037, https://doi.org/10.3389/fmed.2021.789037.
[147] Salem HA, Wadie W, Effect of Niacin on Inflammation and Angiogenesis in a Murine Model of Ulcerative Colitis, Sci Rep 7 (2017) 7139, https://doi.org/10.1038/s41598-017-07280-y.
[148] Li J, Kong D, Wang Q, Wu W, Tang Y, Bai T, Guo L, Wei L, Zhang Q, Yu Y, Qian Y, Zuo S, Liu G, Liu Q, Wu S, Zang Y, Zhu Q, Jia D, Wang Y, Yao W, Ji Y, Yin H, Nakamura M, Lazarus M, Breyer RM, Wang L, Yu Y, Niacin ameliorates ulcerative colitis via prostaglandin D(2)-mediated D prostanoid receptor 1 activation, EMBO Mol Med 9 (2017) 571-588, https://doi.org/10.15252/emmm.201606987.
[149] Yuan SN, Wang MX, Han JL, Feng CY, Wang M, Wang M, Sun JY, Li NY, Simal-Gandara J, Liu C, Improved colonic inflammation by nervonic acid via inhibition of NF-kappaB signaling pathway of DSS-induced colitis mice, Phytomedicine 112 (2023) 154702, https://doi.org/10.1016/j.phymed.2023.154702.
[150] Bastaki SMA, Amir N, Adeghate E, Ojha S, Nerolidol, a sesquiterpene, attenuates oxidative stress and inflammation in acetic acid-induced colitis in rats, Mol Cell Biochem 476 (2021) 3497-3512, https://doi.org/10.1007/s11010-021-04094-5.
[151] Min X, Guo Y, Zhou Y, Chen X, Protection against Dextran Sulfate Sodium-Induced Ulcerative Colitis in Mice by Neferine, A Natural Product from Nelumbo nucifera Gaertn, Cell Journal 22 (2021) 523-531, https://doi.org/10.22074/cellj.2021.6918.
[152] Wu X, Guo Y, Min X, Pei L, Chen X, Neferine, a Bisbenzylisoquinoline Alkaloid, Ameliorates Dextran Sulfate Sodium-Induced Ulcerative Colitis, Am J Chin Med 46 (2018) 1263-1279, https://doi.org/10.1142/S0192415X18500660.
[153] Cao H, Liu J, Shen P, Cai J, Han Y, Zhu K, Fu Y, Zhang N, Zhang Z, Cao Y, Protective Effect of Naringin on DSS-Induced Ulcerative Colitis in Mice, J Agric Food Chem 66 (2018) 13133-13140, https://doi.org/10.1021/acs.jafc.8b03942.
[154] Cao R, Wu X, Guo H, Pan X, Huang R, Wang G, Liu J, Naringin Exhibited Therapeutic Effects against DSS-Induced Mice Ulcerative Colitis in Intestinal Barrier-Dependent Manner, Molecules 26 (2021) 6604, https://doi.org/10.3390/molecules26216604.
[155] Dong J, Chen Y, Yang F, Zhang W, Wei K, Xiong Y, Wang L, Zhou Z, Li C, Wang J, Chen D, Naringin Exerts Therapeutic Effects on Mice Colitis: A Study Based on Transcriptomics Combined With Functional Experiments, Front Pharmacol 12 (2021) 729414, https://doi.org/10.3389/fphar.2021.729414.

[156] Hambardikar VR, Mandlik DS, Protective effect of naringin ameliorates TNBS-induced colitis in rats via improving antioxidant status and pro-inflammatory cytokines, Immunopharmacol Immunotoxicol 44 (2022) 373-386, https://doi.org/10.1080/08923973.2022.2049813.
[157] Al-Rejaie SS, Abuohashish HM, Al-Enazi MM, Al-Assaf AH, Parmar MY, Ahmed MM, Protective effect of naringenin on acetic acid-induced ulcerative colitis in rats, World J Gastroenterol 19 (2013) 5633-5644, https://doi.org/10.3748/wjg.v19.i34.5633.
[158] Dou W, Zhang J, Sun A, Zhang E, Ding L, Mukherjee S, Wei X, Chou G, Wang ZT, Mani S, Protective effect of naringenin against experimental colitis via suppression of Toll-like receptor 4/NF-kappaB signalling, Br J Nutr 110 (2013) 599-608, https://doi.org/10.1017/S0007114512005594.
[159] Ismail Abo El-Fadl HM, Mohamed MFA, Targeting endoplasmic reticulum stress, Nrf-2/HO-1, and NF-kappaB by myristicin and its role in attenuation of ulcerative colitis in rats, Life Sci 311 (2022) 121187, https://doi.org/10.1016/j.lfs.2022.121187.
[160] Zhao J, Hong T, Dong M, Meng Y, Mu J, Protective effect of myricetin in dextran sulphate sodium-induced murine ulcerative colitis, Mol Med Rep 7 (2013) 565-570, https://doi.org/10.3892/mmr.2012.1225.
[161] Qu X, Li Q, Song Y, Xue A, Liu Y, Qi D, Dong H, Potential of myricetin to restore the immune balance in dextran sulfate sodium-induced acute murine ulcerative colitis, J Pharm Pharmacol 72 (2020) 92-100, https://doi.org/10.1111/jphp.13197.
[162] Yuan J, Cheng W, Zhang G, Ma Q, Li X, Zhang B, Hu T, Song G, Protective effects of iridoid glycosides on acute colitis via inhibition of the inflammatory response mediated by the STAT3/NF-small ka, CyrillicB pathway, Int Immunopharmacol 81 (2020) 106240, https://doi.org/10.1016/j.intimp.2020.106240.
[163] Li J, Zhang JL, Gong XP, Xiao M, Song YY, Pi HF, Du G, Anti-inflammatory Activity of Mollugin on DSS-induced Colitis in Mice, Curr Med Sci 40 (2020) 910-916, https://doi.org/10.1007/s11596-020-2262-5.
[164] Liang H, Cheng R, Wang J, Xie H, Li R, Shimizu K, Zhang C, Mogrol, an aglycone of mogrosides, attenuates ulcerative colitis by promoting AMPK activation, Phytomedicine 81 (2021) 153427, https://doi.org/10.1016/j.phymed.2020.153427.
[165] Vochyanova Z, Bartosova L, Bujdakova V, Fictum P, Husnik R, Suchy P, Smejkal K, Hosek J, Diplacone and mimulone ameliorate dextran sulfate sodium-induced colitis in rats, Fitoterapia 101 (2015) 201-207, https://doi.org/10.1016/j.fitote.2015.01.012.
[166] Zhou P, Lai J, Li Y, Deng J, Zhao C, Huang Q, Yang F, Yang S, Wu Y, Tang X, Huang F, Wang L, Huang X, Zou W, Wu J, Methyl Gallate Alleviates Acute Ulcerative Colitis by Modulating Gut Microbiota and Inhibiting TLR4/NF-kappaB Pathway, Int J Mol Sci 23 (2022) 14024, https://doi.org/10.3390/ijms232214024.
[167] Ghasemi-Pirbaluti M, Motaghi E, Bozorgi H, The effect of menthol on acute experimental colitis in rats, Eur J Pharmacol 805 (2017) 101-107, https://doi.org/10.1016/j.ejphar.2017.03.003.
[168] Ahmedy OA, Ibrahim SM, Salem HH, Kandil EA, Antiulcerogenic effect of melittin via mitigating TLR4/TRAF6 mediated NF-kappaB and p38MAPK pathways in acetic acid-induced ulcerative colitis in mice, Chem Biol Interact 331 (2020) 109276, https://doi.org/10.1016/j.cbi.2020.109276.

[169] Yan X, Lu QG, Zeng L, Li XH, Liu Y, Du XF, Bai GM, Synergistic protection of astragalus polysaccharides and matrine against ulcerative colitis and associated lung injury in rats, World J Gastroenterol 26 (2020) 55-69, https://doi.org/10.3748/wjg.v26.i1.55.

[170] Yao H, Shi Y, Yuan J, Sa R, Chen W, Wan X, Matrine protects against DSS-induced murine colitis by improving gut barrier integrity, inhibiting the PPAR-alpha signaling pathway, and modulating gut microbiota, Int Immunopharmacol 100 (2021) 108091, https://doi.org/10.1016/j.intimp.2021.108091.

[171] Tekeli IO, Atessahin A, Sakin F, Aslan A, Ceribasi S, Yipel M, Protective effects of conventional and colon-targeted lycopene and linalool on ulcerative colitis induced by acetic acid in rats, Inflammopharmacology 27 (2018) 313-322, https://doi.org/10.1007/s10787-018-0485-x.

[172] Li Y, Pan X, Yin M, Li C, Han L, Preventive Effect of Lycopene in Dextran Sulfate Sodium-Induced Ulcerative Colitis Mice through the Regulation of TLR4/TRIF/NF-kappaB Signaling Pathway and Tight Junctions, J Agric Food Chem 69 (2021) 13500-13509, https://doi.org/10.1021/acs.jafc.1c05128.

[173] Vukelic I, Detel D, Baticic L, Potocnjak I, Domitrovic R, Luteolin ameliorates experimental colitis in mice through ERK-mediated suppression of inflammation, apoptosis and autophagy, Food Chem Toxicol 145 (2020) 111680, https://doi.org/10.1016/j.fct.2020.111680.

[174] Xie X, Zhao M, Huang S, Li P, Chen P, Luo X, Wang Q, Pan Z, Li X, Chen J, Chen B, Zhou L, Luteolin alleviates ulcerative colitis by restoring the balance of NCR(-)ILC3/NCR(+)ILC3 to repairing impaired intestinal barrier, Int Immunopharmacol 112 (2022) 109251, https://doi.org/10.1016/j.intimp.2022.109251.

[175] Li B, Du P, Du Y, Zhao D, Cai Y, Yang Q, Guo Z, Luteolin alleviates inflammation and modulates gut microbiota in ulcerative colitis rats, Life Sci 269 (2021) 119008, https://doi.org/10.1016/j.lfs.2020.119008.

[176] Liu S, Shen H, Li J, Gong Y, Bao H, Zhang J, Hu L, Wang Z, Gong J, Loganin inhibits macrophage M1 polarization and modulates sirt1/NF-kappaB signaling pathway to attenuate ulcerative colitis, Bioengineered 11 (2020) 628-639, https://doi.org/10.1080/21655979.2020.1774992.

[177] Zhang Z, Yang L, Wang B, Zhang L, Zhang Q, Li D, Zhang S, Gao H, Wang X, Protective role of liriodendrin in mice with dextran sulphate sodium-induced ulcerative colitis, Int Immunopharmacol 52 (2017) 203-210, https://doi.org/10.1016/j.intimp.2017.09.012.

[178] Liu S, Zhang S, Lv X, Lu J, Ren C, Zeng Z, Zheng L, Zhou X, Fu H, Zhou D, Chen Y, Limonin ameliorates ulcerative colitis by regulating STAT3/miR-214 signaling pathway, Int Immunopharmacol 75 (2019) 105768, https://doi.org/10.1016/j.intimp.2019.105768.

[179] Song C, Chen J, Li X, Yang R, Cao X, Zhou L, Zhou Y, Ying H, Zhang Q, Sun Y, Limonin ameliorates dextran sulfate sodium-induced chronic colitis in mice by inhibiting PERK-ATF4-CHOP pathway of ER stress and NF-kappaB signaling, Int Immunopharmacol 90 (2021) 107161, https://doi.org/10.1016/j.intimp.2020.107161.

[180] Zhang J, Xu X, Li N, Cao L, Sun Y, Wang J, He S, Si J, Qing D, Licoflavone B, an isoprene flavonoid derived from licorice residue, relieves dextran sodium sulfate-induced ulcerative colitis by rebuilding the gut barrier and regulating intestinal microflora, Eur J Pharmacol 916 (2022) 174730, https://doi.org/10.1016/j.ejphar.2021.174730.

[181] Zhang J, Cao L, Sun Y, Qing DG, Xu XQ, Wang JC, Si JY, Li N, The Regulatory Effects of Licochalcone A on the Intestinal Epithelium and Gut Microbiota in Murine Colitis, Molecules 26 (2021) 4149, https://doi.org/10.3390/molecules26144149.

[182] Liu D, Huo X, Gao L, Zhang J, Ni H, Cao L, NF-kappaB and Nrf2 pathways contribute to the protective effect of Licochalcone A on dextran sulphate sodium-induced ulcerative colitis in mice, Biomed Pharmacother 102 (2018) 922-929, https://doi.org/10.1016/j.biopha.2018.03.130.

[183] Zheng S, Zhuang T, Tang Y, Wu R, Xu T, Leng T, Wang Y, Lin Z, Ji M, Leonurine protects against ulcerative colitis by alleviating inflammation and modulating intestinal microflora in mouse models, Exp Ther Med 22 (2021) 1199, https://doi.org/10.3892/etm.2021.10633.

[184] Coburn LA, Gong X, Singh K, Asim M, Scull BP, Allaman MM, Williams CS, Rosen MJ, Washington MK, Barry DP, Piazuelo MB, Casero RA, Jr., Chaturvedi R, Zhao Z, Wilson KT, L-arginine supplementation improves responses to injury and inflammation in dextran sulfate sodium colitis, PLoS One 7 (2012) e33546, https://doi.org/10.1371/journal.pone.0033546.

[185] Tashita C, Hoshi M, Hirata A, Nakamoto K, Ando T, Hattori T, Yamamoto Y, Tezuka H, Tomita H, Hara A, Saito K, Kynurenine plays an immunosuppressive role in 2,4,6-trinitrobenzene sulfate-induced colitis in mice, World Journal of Gastroenterology 26 (2020) 918-932, https://doi.org/10.3748/wjg.v26.i9.918.

[186] Tang Q, Fan H, Shou ZX, Liu XX, Study on protective mechanism of kushenin injectionon colonic mucosa of experimental colitis rats, China J. Chin. Mater. Med. 37 (2012) 1814-1817,

[187] Farombi EO, Adedara IA, Ajayi BO, Ayepola OR, Egbeme EE, Kolaviron, a natural antioxidant and anti-inflammatory phytochemical prevents dextran sulphate sodium-induced colitis in rats, Basic & Clinical Pharmacology & Toxicology 113 (2013) 49-55, https://doi.org/10.1111/bcpt.12050.

[188] Qu Y, Li X, Xu F, Zhao S, Wu X, Wang Y, Xie J, Kaempferol Alleviates Murine Experimental Colitis by Restoring Gut Microbiota and Inhibiting the LPS-TLR4-NF-kappaB Axis, Front Immunol 12 (2021) 679897, https://doi.org/10.3389/fimmu.2021.679897.

[189] Park MY, Ji GE, Sung MK, Dietary kaempferol suppresses inflammation of dextran sulfate sodium-induced colitis in mice, Dig Dis Sci 57 (2012) 355-363, https://doi.org/10.1007/s10620-011-1883-8.

[190] Hua Y, Liu R, Lu M, Guan X, Zhuang S, Tian Y, Zhang Z, Cui L, Juglone regulates gut microbiota and Th17/Treg balance in DSS-induced ulcerative colitis, Int Immunopharmacol 97 (2021) 107683, https://doi.org/10.1016/j.intimp.2021.107683.

[191] Zhang JL, Zhang MN, Wang HG, Yang XZ, Yu CG, Jatrorrhizine alleviates ulcerative colitis via regulating gut microbiota and NOS2 expression, Gut Pathog 14 (2022) 41, https://doi.org/10.1186/s13099-022-00514-z.

[192] Niu S, Jing M, Wen J, Wei S, Li H, Li X, Ma X, Zhao Y, Jatrorrhizine Alleviates DSS-Induced Ulcerative Colitis by Regulating the Intestinal Barrier Function and Inhibiting TLR4/MyD88/NF-kappaB Signaling Pathway, Evid Based Complement Alternat Med 2022 (2022) 3498310, https://doi.org/10.1155/2022/3498310.

[193] Liu MX, Li T, Wang WG, Guo J, Wang RR, He HP, Li SQ, Li YP, Regulatory effect of isovitexin on MAPK/NF-kappaB signal in mice with acute ulcerative colitis, J Asian Nat Prod Res 25 (2023) 765-782, https://doi.org/10.1080/10286020.2022.2142121.

[194] Zhou Y, Zhong B, Min X, Hou Y, Lin L, Wu Q, Shi J, Chen X, Therapeutic potential of isobavachalcone, a natural flavonoid, in murine experimental colitis by

inhibiting NF-kappaB p65, Phytother Res 35 (2021) 5861-5870, https://doi.org/10.1002/ptr.7246.
[195] Gao WY, Zhang LD, Wang XQ, Yu L, Wang CH, Gong Y, The combination of indirubin and isatin attenuates dextran sodium sulfate induced ulcerative colitis in mice, Biochemistry and Cell Biology 96 (2018) 636-645, https://doi.org/10.1139/bcb-2018-0041.
[196] Socca EA, Luiz-Ferreira A, de Faria FM, de Almeida AC, Dunder RJ, Manzo LP, Brito AR, Inhibition of tumor necrosis factor-alpha and cyclooxigenase-2 by Isatin: a molecular mechanism of protection against TNBS-induced colitis in rats, Chem Biol Interact 209 (2014) 48-55, https://doi.org/10.1016/j.cbi.2013.11.019.
[197] Huangfu LX, Cai XT, Yang JN, Wang HC, Li YX, Dai ZF, Yang RL, Lin XH, Irisin attenuates inflammation in a mouse model of ulcerative colitis by altering the intestinal microbiota, Exp Ther Med 22 (2021) 1433, https://doi.org/10.3892/etm.2021.10868.
[198] Peng C, Wu C, Xu X, Pan L, Lou Z, Zhao Y, Jiang H, He Z, Ruan B, Indole-3-carbinol ameliorates necroptosis and inflammation of intestinal epithelial cells in mice with ulcerative colitis by activating aryl hydrocarbon receptor, Exp Cell Res 404 (2021) 112638, https://doi.org/10.1016/j.yexcr.2021.112638.
[199] Gao W, Guo Y, Wang C, Lin Y, Yu L, Sheng T, Wu Z, Gong Y, Indirubin ameliorates dextran sulfate sodium-induced ulcerative colitis in mice through the inhibition of inflammation and the induction of Foxp3-expressing regulatory T cells, Acta Histochem 118 (2016) 606-614, https://doi.org/10.1016/j.acthis.2016.06.004.
[200] Luo M, Luo Y, Imperatorin Relieved Ulcerative Colitis by Regulating the Nrf-2/ARE/HO-1 Pathway in Rats, Inflammation 44 (2021) 558-569, https://doi.org/10.1007/s10753-020-01353-3.
[201] Zhang H, Zhuo S, Song D, Wang L, Gu J, Ma J, Gu Y, Ji M, Chen M, Guo Y, Icariin Inhibits Intestinal Inflammation of DSS-Induced Colitis Mice Through Modulating Intestinal Flora Abundance and Modulating p-p65/p65 Molecule, Turk J Gastroenterol 32 (2021) 382-392, https://doi.org/10.5152/tjg.2021.20282.
[202] Cheng C, Zhang W, Zhang C, Ji P, Wu X, Sha Z, Chen X, Wang Y, Chen Y, Cheng H, Shi L, Hyperoside Ameliorates DSS-Induced Colitis through MKRN1-Mediated Regulation of PPARgamma Signaling and Th17/Treg Balance, J Agric Food Chem 69 (2021) 15240-15251, https://doi.org/10.1021/acs.jafc.1c06292.
[203] Elmaksoud HAA, Motawea MH, Desoky AA, Elharrif MG, Ibrahimi A, Hydroxytyrosol alleviate intestinal inflammation, oxidative stress and apoptosis resulted in ulcerative colitis, Biomedicine & Pharmacotherapy 142 (2021) 112073, https://doi.org/10.1016/j.biopha.2021.112073.
[204] Miao F, Hydroxytyrosol alleviates dextran sodium sulfate-induced colitis by inhibiting NLRP3 inflammasome activation and modulating gut microbiota in vivo, Nutrition 97 (2022) 111579, https://doi.org/10.1016/j.nut.2021.111579.
[205] Feng Z, Zhou P, Wu X, Zhang J, Zhang M, Hydroxysafflor yellow A protects against ulcerative colitis via suppressing TLR4/NF-kappaB signaling pathway, Chemical Biology & Drug Design 99 (2022) 897-907, https://doi.org/10.1111/cbdd.14045.
[206] Wang N, Kong R, Han W, Bao W, Shi Y, Ye L, Lu J, Honokiol alleviates ulcerative colitis by targeting PPAR-gamma-TLR4-NF-kappaB signaling and suppressing gasdermin-D-mediated pyroptosis in vivo and in vitro, Int Immunopharmacol 111 (2022) 109058, https://doi.org/10.1016/j.intimp.2022.109058.
[207] Wang L, Wang J, Honokiol Ameliorates DSS-Induced Mouse Colitis by Inhibiting Inflammation and Oxidative Stress and Improving the Intestinal Barrier, Oxid

Med Cell Longev 2022 (2022) 1755608, https://doi.org/10.1155/2022/1755608.
[208] Liu J, Shi L, Huang W, Zheng Z, Huang X, Su Y, Homoharringtonine Attenuates Dextran Sulfate Sodium-Induced Colitis by Inhibiting NF-kappaB Signaling, Mediators Inflamm 2022 (2022) 3441357, https://doi.org/10.1155/2022/3441357.
[209] Chen C, Liang H, Wang J, Ren G, Li R, Cui ZG, Zhang C, Heterophyllin B an Active Cyclopeptide Alleviates Dextran Sulfate Sodium-Induced Colitis by Modulating Gut Microbiota and Repairing Intestinal Mucosal Barrier via AMPK Activation, Mol Nutr Food Res 66 (2022) e2101169, https://doi.org/10.1002/mnfr.202101169.
[210] Guazelli CFS, Fattori V, Ferraz CR, Borghi SM, Casagrande R, Baracat MM, Verri WA, Jr., Antioxidant and anti-inflammatory effects of hesperidin methyl chalcone in experimental ulcerative colitis, Chem Biol Interact 333 (2021) 109315, https://doi.org/10.1016/j.cbi.2020.109315.
[211] Xu L, Yang ZL, Li P, Zhou YQ, Modulating effect of Hesperidin on experimental murine colitis induced by dextran sulfate sodium, Phytomedicine 16 (2009) 989-995, https://doi.org/10.1016/j.phymed.2009.02.021.
[212] Shafik NM, Gaber RA, Mohamed DA, Ebeid AM, Hesperidin modulates dextran sulfate sodium-induced ulcerative colitis in rats: Targeting sphingosine kinase-1-sphingosine 1 phosphate signaling pathway, mitochondrial biogenesis, inflammation, and apoptosis, J Biochem Mol Toxicol 33 (2019) e22312, https://doi.org/10.1002/jbt.22312.
[213] Polat FR, Karaboga I, Immunohistochemical examination of anti-inflammatory and anti-apoptotic effects of hesperetin on trinitrobenzene sulfonic acid induced colitis in rats, Biotechnic & Histochemistry 94 (2019) 151-158, https://doi.org/10.1080/10520295.2018.1530454.
[214] Zhang J, Lei H, Hu X, Dong W, Hesperetin ameliorates DSS-induced colitis by maintaining the epithelial barrier via blocking RIPK3/MLKL necroptosis signaling, Eur J Pharmacol 873 (2020) 172992, https://doi.org/10.1016/j.ejphar.2020.172992.
[215] Sethuraman SN, Swaminathan S, Nelson SB, Palaninathan PS, Gopalan TK, Velayudham P, Modulation of PPARgamma and TNFalpha by emu oil and glycyrrhizin in ulcerative colitis, Inflammopharmacology 23 (2015) 47-56, https://doi.org/10.1007/s10787-014-0226-8.
[216] Kudo T, Okamura S, Zhang Y, Masuo T, Mori M, Topical application of glycyrrhizin preparation ameliorates experimentally induced colitis in rats, World J Gastroenterol 17 (2011) 2223-2228, https://doi.org/10.3748/wjg.v17.i17.2223.
[217] Jeon YD, Kang SH, Bang KS, Chang YN, Lee JH, Jin JS, Glycyrrhetic Acid Ameliorates Dextran Sulfate Sodium-Induced Ulcerative Colitis in Vivo, Molecules 21 (2016) 523, https://doi.org/10.3390/molecules21040523.
[218] Deger C, Erbil Y, Giris M, Yanik BT, Tunca F, Olgac V, Abbasoglu SD, Oztezcan S, Toker G, The effect of glutamine on pancreatic damage in TNBS-induced colitis, Dig Dis Sci 51 (2006) 1841-1846, https://doi.org/10.1007/s10620-006-9189-y.
[219] Yan S, Hui Y, Li J, Xu X, Li Q, Wei H, Glutamine relieves oxidative stress through PI3K/Akt signaling pathway in DSS-induced ulcerative colitis mice, Iran J Basic Med Sci 23 (2020) 1124-1129, https://doi.org/10.22038/ijbms.2020.39815.9436.

[220] Li JM, Jia HY, Wang JJ, Yu Q, Li S, Effects of Glutamine on the colon of mice subjected to colitis gravis, Chinese Journal of Applied Physiology 25 (2009) 268-272, https://doi.org/10.13459/j.cnki.cjap.2009.02.030.

[221] Li TT, Zhang JF, Fei SJ, Zhu SP, Zhu JZ, Qiao X, Liu ZB, Glutamate microinjection into the hypothalamic paraventricular nucleus attenuates ulcerative colitis in rats, Acta Pharmacol Sin 35 (2014) 185-194, https://doi.org/10.1038/aps.2013.140.

[222] El-Ashmawy NE, Khedr NF, El-Bahrawy HA, El-Adawy SA, Downregulation of iNOS and elevation of cAMP mediate the anti-inflammatory effect of glabridin in rats with ulcerative colitis, Inflammopharmacology 26 (2018) 551-559, https://doi.org/10.1007/s10787-017-0373-9.

[223] Tian M, Ma P, Zhang Y, Mi Y, Fan D, Ginsenoside Rk3 alleviated DSS-induced ulcerative colitis by protecting colon barrier and inhibiting NLRP3 inflammasome pathway, Int Immunopharmacol 85 (2020) 106645, https://doi.org/10.1016/j.intimp.2020.106645.

[224] Chen X, Xu T, Lv X, Zhang J, Liu S, Ginsenoside Rh2 alleviates ulcerative colitis by regulating the STAT3/miR-214 signaling pathway, J Ethnopharmacol 274 (2021) 113997, https://doi.org/10.1016/j.jep.2021.113997.

[225] Ye H, Wu Q, Zhu Y, Guo C, Zheng X, Ginsenoside Rh2 alleviates dextran sulfate sodium-induced colitis via augmenting TGFbeta signaling, Mol Biol Rep 41 (2014) 5485-5490, https://doi.org/10.1007/s11033-014-3422-0.

[226] Liu D, Tian Q, Liu K, Ren F, Liu G, Zhou J, Yuan L, Fang Z, Zou B, Wang S, Ginsenoside Rg3 Ameliorates DSS-Induced Colitis by Inhibiting NLRP3 Inflammasome Activation and Regulating Microbial Homeostasis, J Agric Food Chem 71 (2023) 3472-3483, https://doi.org/10.1021/acs.jafc.2c07766.

[227] Long J, Liu XK, Kang ZP, Wang MX, Zhao HM, Huang JQ, Xiao QP, Liu DY, Zhong YB, Ginsenoside Rg1 ameliorated experimental colitis by regulating the balance of M1/M2 macrophage polarization and the homeostasis of intestinal flora, Eur J Pharmacol 917 (2022) 174742, https://doi.org/10.1016/j.ejphar.2022.174742.

[228] Cheng H, Liu J, Zhang D, Wang J, Tan Y, Feng W, Peng C, Ginsenoside Rg1 Alleviates Acute Ulcerative Colitis by Modulating Gut Microbiota and Microbial Tryptophan Metabolism, Front Immunol 13 (2022) 817600, https://doi.org/10.3389/fimmu.2022.817600.

[229] Yang XL, Guo TK, Wang YH, Gao MT, Qin H, Wu YJ, Therapeutic effect of ginsenoside Rd in rats with TNBS-induced recurrent ulcerative colitis, Archives of Pharmacal Research 35 (2012) 1231-1239, https://doi.org/10.1007/s12272-012-0714-6.

[230] Liu C, Wang J, Yang Y, Liu X, Zhu Y, Zou J, Peng S, Le TH, Chen Y, Zhao S, He B, Mi Q, Zhang X, Du Q, Ginsenoside Rd ameliorates colitis by inducing p62-driven mitophagy-mediated NLRP3 inflammasome inactivation in mice, Biochem Pharmacol 155 (2018) 366-379, https://doi.org/10.1016/j.bcp.2018.07.010.

[231] Qu B, Cao T, Wang M, Wang S, Li W, Li H, Ginsenosides Rd monomer inhibits proinflammatory cytokines production and alleviates DSS-colitis by NF-kappaB and P38MAPK pathways in mice, Immunopharmacol Immunotoxicol 44 (2022) 110-118, https://doi.org/10.1080/08923973.2021.2012482.

[232] Wang L, Shao L, Chen MY, Wang L, Zhang W, Tan FB, Huang WH, Effect of ginsenoside compound K on alleviating colitis via modulating gut microbiota, Chin Med 17 (2022) 146, https://doi.org/10.1186/s13020-022-00701-9.

[233] Xu D, Zhuang L, Gao S, Ma H, Cheng J, Liu J, Liu D, Fu S, Hu G, Orally Administered Ginkgolide C Attenuates DSS-Induced Colitis by Maintaining Gut Barrier Integrity, Inhibiting Inflammatory Responses, and Regulating Intestinal Flora, J Agric Food Chem 70 (2022) 14718-14731, https://doi.org/10.1021/acs.jafc.2c06177.
[234] Chen Y, Le TH, Du Q, Zhao Z, Liu Y, Zou J, Hua W, Liu C, Zhu Y, Genistein protects against DSS-induced colitis by inhibiting NLRP3 inflammasome via TGR5-cAMP signaling, Int Immunopharmacol 71 (2019) 144-154, https://doi.org/10.1016/j.intimp.2019.01.021.
[235] Pu Z, Liu Y, Li C, Xu M, Xie H, Zhao J, Using Network Pharmacology for Systematic Understanding of Geniposide in Ameliorating Inflammatory Responses in Colitis Through Suppression of NLRP3 Inflammasome in Macrophage by AMPK/Sirt1 Dependent Signaling, Am J Chin Med 48 (2020) 1693-1713, https://doi.org/10.1142/S0192415X20500846.
[236] Yu Y, Bian Y, Shi JX, Gu Y, Yuan DP, Yu B, Shi L, Dou DH, Geniposide promotes splenic Treg differentiation to alleviate colonic inflammation and intestinal barrier injury in ulcerative colitis mice, Bioengineered 13 (2022) 14616-14631, https://doi.org/10.1080/21655979.2022.2092678.
[237] Xu B, Li YL, Xu M, Yu CC, Lian MQ, Tang ZY, Li CX, Lin Y, Geniposide ameliorates TNBS-induced experimental colitis in rats via reducing inflammatory cytokine release and restoring impaired intestinal barrier function, Acta Pharmacol Sin 38 (2017) 688-698, https://doi.org/10.1038/aps.2016.168.
[238] Zhang Z, Li Y, Shen P, Li S, Lu X, Liu J, Cao Y, Liu B, Fu Y, Zhang N, Administration of geniposide ameliorates dextran sulfate sodium-induced colitis in mice via inhibition of inflammation and mucosal damage, Int Immunopharmacol 49 (2017) 168-177, https://doi.org/10.1016/j.intimp.2017.05.033.
[239] Yang H, Yue Y, Li Y, Su L, Yan S, Geniposide attenuates dextran sulfate sodium-induced colitis in mice via Nrf-2/HO-1/NF-kappaB pathway, Ann Palliat Med 9 (2020) 2826-2836, https://doi.org/10.21037/apm-20-279.
[240] Xu XM, Yu JP, He XF, Li JH, Yu LL, Yu HG, Effects of garlicin on apoptosis in rat model of colitis, World J Gastroenterol 11 (2005) 4579-4582, https://doi.org/10.3748/wjg.v11.i29.4579.
[241] Xiao HT, Lin CY, Ho DH, Peng J, Chen Y, Tsang SW, Wong M, Zhang XJ, Zhang M, Bian ZX, Inhibitory effect of the gallotannin corilagin on dextran sulfate sodium-induced murine ulcerative colitis, J Nat Prod 76 (2013) 2120-2125, https://doi.org/10.1021/np4006772.
[242] Pandurangan AK, Mohebali N, Esa NM, Looi CY, Ismail S, Saadatdoust Z, Gallic acid suppresses inflammation in dextran sodium sulfate-induced colitis in mice: Possible mechanisms, Int Immunopharmacol 28 (2015) 1034-1043, https://doi.org/10.1016/j.intimp.2015.08.019.
[243] Zhu L, Gu P, Shen H, Gallic acid improved inflammation via NF-kappaB pathway in TNBS-induced ulcerative colitis, Int Immunopharmacol 67 (2019) 129-137, https://doi.org/10.1016/j.intimp.2018.11.049.
[244] Yu TY, Feng YM, Kong WS, Li SN, Sun XJ, Zhou G, Xie RF, Zhou X, Gallic acid ameliorates dextran sulfate sodium-induced ulcerative colitis in mice via inhibiting NLRP3 inflammasome, Front Pharmacol 14 (2023) 1095721, https://doi.org/10.3389/fphar.2023.1095721.
[245] Xuan H, Ou A, Hao S, Shi J, Jin X, Galangin Protects against Symptoms of Dextran Sodium Sulfate-induced Acute Colitis by Activating Autophagy and Modulating

the Gut Microbiota, Nutrients 12 (2020) 347, https://doi.org/10.3390/nu12020347.
[246] Gerges SH, Tolba MF, Elsherbiny DA, El-Demerdash E, The natural flavonoid galangin ameliorates dextran sulphate sodium-induced ulcerative colitis in mice: Effect on Toll-like receptor 4, inflammation and oxidative stress, Basic Clin Pharmacol Toxicol 127 (2020) 10-20, https://doi.org/10.1111/bcpt.13388.
[247] Sangaraju R, Nalban N, Alavala S, Rajendran V, Jerald MK, Sistla R, Protective effect of galangin against dextran sulfate sodium (DSS)-induced ulcerative colitis in Balb/c mice, Inflamm Res 68 (2019) 691-704, https://doi.org/10.1007/s00011-019-01252-w.
[248] Guo W, Hu S, Elgehama A, Shao F, Ren R, Liu W, Zhang W, Wang X, Tan R, Xu Q, Sun Y, Jiao R, Fumigaclavine C ameliorates dextran sulfate sodium-induced murine experimental colitis via NLRP3 inflammasome inhibition, J Pharmacol Sci 129 (2015) 101-106, https://doi.org/10.1016/j.jphs.2015.05.003.
[249] Yang YP, Tong QY, Zheng SH, Zhou MD, Zeng YM, Zhou TT, Anti-inflammatory effect of fucoxanthin on dextran sulfate sodium-induced colitis in mice, Nat Prod Res 34 (2020) 1791-1795, https://doi.org/10.1080/14786419.2018.1528593.
[250] Shi B, Liu S, Huang A, Zhou M, Sun B, Cao H, Shan J, Sun B, Lin J, Revealing the Mechanism of Friedelin in the Treatment of Ulcerative Colitis Based on Network Pharmacology and Experimental Verification, Evid Based Complement Alternat Med 2021 (2021) 4451779, https://doi.org/10.1155/2021/4451779.
[251] Mohamed NI, El-Kashef DH, Suddek GM, Flavocoxid halts both intestinal and extraintestinal alterations in acetic acid-induced colitis in rats, Environ Sci Pollut Res Int 29 (2022) 5945-5959, https://doi.org/10.1007/s11356-021-16092-7.
[252] Sahu BD, Kumar JM, Sistla R, Fisetin, a dietary flavonoid, ameliorates experimental colitis in mice: Relevance of NF-kappaB signaling, J Nutr Biochem 28 (2016) 171-182, https://doi.org/10.1016/j.jnutbio.2015.10.004.
[253] Sadar SS, Vyawahare NS, Bodhankar SL, Ferulic acid ameliorates TNBS-induced ulcerative colitis through modulation of cytokines, oxidative stress, iNOs, COX-2, and apoptosis in laboratory rats, EXCLI J 15 (2016) 482-499, https://doi.org/10.17179/excli2016-393.
[254] Yu S, Qian H, Zhang D, Jiang Z, Ferulic acid relieved ulcerative colitis by inhibiting the TXNIP/NLRP3 pathway in rats, Cell Biol Int 47 (2023) 417-427, https://doi.org/10.1002/cbin.11935.
[255] Shen P, Zhang Z, Zhu K, Cao H, Liu J, Lu X, Li Y, Jing Y, Yuan X, Fu Y, Cao Y, Zhang N, Evodiamine prevents dextran sulfate sodium-induced murine experimental colitis via the regulation of NF-kappaB and NLRP3 inflammasome, Biomed Pharmacother 110 (2019) 786-795, https://doi.org/10.1016/j.biopha.2018.12.033.
[256] Wang MX, Lin L, Chen YD, Zhong YP, Lin YX, Li P, Tian X, Han B, Xie ZY, Liao QF, Evodiamine has therapeutic efficacy in ulcerative colitis by increasing Lactobacillus acidophilus levels and acetate production, Pharmacol Res 159 (2020) 104978, https://doi.org/10.1016/j.phrs.2020.104978.
[257] Zhang Y, Zhang Y, Zhao Y, Wu W, Meng W, Zhou Y, Qiu Y, Li C, Protection against ulcerative colitis and colorectal cancer by evodiamine via anti-inflammatory effects, Mol Med Rep 25 (2022) 188, https://doi.org/10.3892/mmr.2022.12704.
[258] Zhou K, Cheng R, Liu B, Wang L, Xie H, Zhang C, Eupatilin ameliorates dextran sulphate sodium-induced colitis in mice partly through promoting AMPK activation,

Phytomedicine 46 (2018) 46-56, https://doi.org/10.1016/j.phymed.2018.04.033.
[259] Zhou B, Liu J, Wang Y, Wu F, Wang C, Wang C, Liu J, Li P, Protective Effect of Ethyl Rosmarinate against Ulcerative Colitis in Mice Based on Untargeted Metabolomics, Int J Mol Sci 23 (2022) 1256, https://doi.org/10.3390/ijms23031256.
[260] Liu Y, Wei W, Liang S, Fang H, Cao J, Esculentoside A could attenuate apoptosis and inflammation in TNBS-induced ulcerative colitis via inhibiting the nuclear translocation of NF-kappaB, Ann Transl Med 10 (2022) 771, https://doi.org/10.21037/atm-22-2675.
[261] Liu Y, Wei W, Liang S, Fang H, Cao J, Esculentoside A Alleviates Intestinal Dysmotility in Ulcerative Colitis by Regulating H(2)S/CSE and NO/nNOS Systems, Evid Based Complement Alternat Med 2022 (2022) 7757833, https://doi.org/10.1155/2022/7757833.
[262] Hu LH, Liu JY, Yin JB, Eriodictyol attenuates TNBS-induced ulcerative colitis through repressing TLR4/NF-kB signaling pathway in rats, Kaohsiung J Med Sci 37 (2021) 812-818, https://doi.org/10.1002/kjm2.12400.
[263] Wang R, Shen L, Li H, Peng H, Eriodictyol attenuates dextran sodium sulphate-induced colitis in mice by regulating the sonic hedgehog signalling pathway, Pharm Biol 59 (2021) 974-985, https://doi.org/10.1080/13880209.2021.1948066.
[264] Guo G, Shi W, Shi F, Gong W, Li F, Zhou G, She J, Anti-inflammatory effects of eriocitrin against the dextran sulfate sodium-induced experimental colitis in murine model, J Biochem Mol Toxicol 33 (2019) e22400, https://doi.org/10.1002/jbt.22400.
[265] Dou B, Hu W, Song M, Lee RJ, Zhang X, Wang D, Anti-inflammation of Erianin in dextran sulphate sodium-induced ulcerative colitis mice model via collaborative regulation of TLR4 and STAT3, Chem Biol Interact 324 (2020) 109089, https://doi.org/10.1016/j.cbi.2020.109089.
[266] Gao Y, Zhou B, Zhang H, Chen L, Wang X, Chen H, Zhou L, l-Ergothioneine Exhibits Protective Effects against Dextran Sulfate Sodium-Induced Colitis in Mice, ACS Omega 7 (2022) 21554-21565, https://doi.org/10.1021/acsomega.2c01350.
[267] Pang L, Wang T, Liao Q, Cheng Y, Wang D, Li J, Fu C, Zhang C, Zhang J, Protective role of ergothioneine isolated from Pleurotus ostreatus against dextran sulfate sodium-induced ulcerative colitis in rat model, J Food Sci 87 (2022) 415-426, https://doi.org/10.1111/1750-3841.15982.
[268] He J, Liu L, Liu X, Chen H, Liu K, Huang N, Wang Y, Epoxymicheliolide prevents dextran sulfate sodium-induced colitis in mice by inhibiting TAK1-NF-kappaB pathway and activating Keap1-NRF2 signaling in macrophages, Int Immunopharmacol 113 (2022) 109404, https://doi.org/10.1016/j.intimp.2022.109404.
[269] Bitzer ZT, Elias RJ, Vijay-Kumar M, Lambert JD, (-)-Epigallocatechin-3-gallate decreases colonic inflammation and permeability in a mouse model of colitis, but reduces macronutrient digestion and exacerbates weight loss, Mol Nutr Food Res 60 (2016) 2267-2274, https://doi.org/10.1002/mnfr.201501042.
[270] Xu Z, Wei C, Zhang RU, Yao J, Zhang D, Wang L, Epigallocatechin-3-gallate-induced inhibition of interleukin-6 release and adjustment of the regulatory T/T helper 17 cell balance in the treatment of colitis in mice, Exp Ther Med 10 (2015) 2231-2238, https://doi.org/10.3892/etm.2015.2824.
[271] Diwan B, Sharma R, Green tea EGCG effectively alleviates experimental colitis in middle-aged male mice by attenuating multiple aspects of oxi-inflammatory stress

and cell cycle deregulation, Biogerontology 23 (2022) 789-807, https://doi.org/10.1007/s10522-022-09976-9.
[272] Zhang H, Deng A, Zhang Z, Yu Z, Liu Y, Peng S, Wu L, Qin H, Wang W, The protective effect of epicatechin on experimental ulcerative colitis in mice is mediated by increasing antioxidation and by the inhibition of NF-kappaB pathway, Pharmacol Rep 68 (2016) 514-520, https://doi.org/10.1016/j.pharep.2015.12.011.
[273] Xu B, Huang SW, Chen YP, Wang Q, Luo S, Li YY, Wang XJ, Chen JY, Luo X, Zhou L, Synergistic effect of combined treatment with baicalin and emodin on DSS-induced colitis in mouse, Phytotherapy Research 35 (2021) 5708-5719, https://doi.org/10.1002/ptr.7230.
[274] Luo S, He J, Huang S, Wang X, Su Y, Li Y, Chen Y, Yang G, Huang B, Guo S, Zhou L, Luo X, Emodin targeting the colonic metabolism via PPARgamma alleviates UC by inhibiting facultative anaerobe, Phytomedicine 104 (2022) 154106, https://doi.org/10.1016/j.phymed.2022.154106.
[275] Luo S, Deng X, Liu Q, Pan Z, Zhao Z, Zhou L, Luo X, Emodin ameliorates ulcerative colitis by the flagellin-TLR5 dependent pathway in mice, Int Immunopharmacol 59 (2018) 269-275, https://doi.org/10.1016/j.intimp.2018.04.010.
[276] Marin M, Maria Giner R, Rios JL, Recio MC, Intestinal anti-inflammatory activity of ellagic acid in the acute and chronic dextrane sulfate sodium models of mice colitis, J Ethnopharmacol 150 (2013) 925-934, https://doi.org/10.1016/j.jep.2013.09.030.
[277] Lin Y, Wu Y, Su J, Wang M, Wu X, Su Z, Yi X, Wei L, Cai J, Sun Z, Therapeutic role of d-pinitol on experimental colitis via activating Nrf2/ARE and PPAR-γ/NF-κB signaling pathways, Food & Function 12 (2021) 2554-2568, https://doi.org/10.1039/d0fo03139a.
[278] Zheng Z, Dai Z, Cao Y, Shen Q, Zhang Y, Docosapentaenoic acid (DPA, 22:5n-3) ameliorates inflammation in an ulcerative colitis model, Food Funct 10 (2019) 4199-4209, https://doi.org/10.1039/c8fo02338g.
[279] Dong Y, Huang C, Yang J, Zheng Z, Dai Z, Docosapentaenoic Acid (DPA, 22:5n-3) Alleviates Ulcerative Colitis via Modification of Gut Microbiota and Their Metabolism, Nutrients 14 (2022) 4204, https://doi.org/10.3390/nu14194204.
[280] Yu L, Yan J, Sun Z, D-limonene exhibits anti-inflammatory and antioxidant properties in an ulcerative colitis rat model via regulation of iNOS, COX-2, PGE2 and ERK signaling pathways, Mol Med Rep 15 (2017) 2339-2346, https://doi.org/10.3892/mmr.2017.6241.
[281] Shalkami AS, Hassan M, Bakr AG, Anti-inflammatory, antioxidant and anti-apoptotic activity of diosmin in acetic acid-induced ulcerative colitis, Human & Experimental Toxicology 37 (2018) 78-86, https://doi.org/10.1177/0960327117694075.
[282] Tang X, Huang G, Zhang T, Li S, Elucidation of colon-protective efficacy of diosgenin in experimental TNBS-induced colitis: inhibition of NF-kappaB/IkB-alpha and Bax/Caspase-1 signaling pathways, Biosci Biotechnol Biochem 84 (2020) 1903-1912, https://doi.org/10.1080/09168451.2020.1776590.
[283] Li H, Pang B, Nie B, Qu S, Zhang K, Xu J, Yang M, Liu J, Li S, Dioscin promotes autophagy by regulating the AMPK-mTOR pathway in ulcerative colitis, Immunopharmacol Immunotoxicol 44 (2022) 238-246, https://doi.org/10.1080/08923973.2022.2037632.
[284] Cai J, Liu J, Fan P, Dong X, Zhu K, Liu X, Zhang N, Cao Y, Dioscin prevents DSS-induced colitis in mice with enhancing intestinal barrier function and reducing

colon inflammation, Int Immunopharmacol 99 (2021) 108015, https://doi.org/10.1016/j.intimp.2021.108015.

[285] Wu MM, Wang QM, Huang BY, Mai CT, Wang CL, Wang TT, Zhang XJ, Dioscin ameliorates murine ulcerative colitis by regulating macrophage polarization, Pharmacol Res 172 (2021) 105796, https://doi.org/10.1016/j.phrs.2021.105796.

[286] Guo Y, Wu X, Wu Q, Lu Y, Shi J, Chen X, Dihydrotanshinone I, a natural product, ameliorates DSS-induced experimental ulcerative colitis in mice, Toxicol Appl Pharmacol 344 (2018) 35-45, https://doi.org/10.1016/j.taap.2018.02.018.

[287] Li C, Dong N, Wu B, Mo Z, Xie J, Lu Q, Dihydroberberine, an isoquinoline alkaloid, exhibits protective effect against dextran sulfate sodium-induced ulcerative colitis in mice, Phytomedicine 90 (2021) 153631, https://doi.org/10.1016/j.phymed.2021.153631.

[288] Jiang M, Zhong G, Zhu Y, Wang L, He Y, Sun Q, Wu X, You X, Gao S, Tang D, Wang D, Retardant effect of dihydroartemisinin on ulcerative colitis in a JAK2/STAT3-dependent manner, Acta Biochim Biophys Sin (Shanghai) 53 (2021) 1113-1123, https://doi.org/10.1093/abbs/gmab097.

[289] Li N, Sun W, Zhou X, Gong H, Chen Y, Chen D, Xiang F, Dihydroartemisinin Protects against Dextran Sulfate Sodium-Induced Colitis in Mice through Inhibiting the PI3K/AKT and NF-kappaB Signaling Pathways, Biomed Res Int 2019 (2019) 1415809, https://doi.org/10.1155/2019/1415809.

[290] Lv Q, Xing Y, Liu Y, Chen Q, Xu J, Hu L, Zhang Y, Didymin switches M1-like toward M2-like macrophage to ameliorate ulcerative colitis via fatty acid oxidation, Pharmacol Res 169 (2021) 105613, https://doi.org/10.1016/j.phrs.2021.105613.

[291] Yuan H, Ji WS, Wu KX, Jiao JX, Sun LH, Feng YT, Anti-inflammatory effect of Diammonium Glycyrrhizinate in a rat model of ulcerative colitis, World J Gastroenterol 12 (2006) 4578-4581, https://doi.org/10.3748/wjg.v12.i28.4578.

[292] Zohny MH, Alrouji M, Alhajlah S, AlOmeir O, Ewees MGE, Ghaffar DMA, El Adle Khalaf N, Mohammed OA, Abdeldaiem MSI, El-Bahouty WB, Elrabat A, Zakaria S, Abdel-Nasser ZM, Haleem AA, El-Gharbawy DM, Abdelhady R, Kaddah MMY, Shata A, Saber S, Diacetylrhein, an anthraquinone antiarthritic agent, suppresses dextran sodium sulfate-induced inflammation in rats: A possible mechanism for a protective effect against ulcerative colitis, Biomed Pharmacother 154 (2022) 113651, https://doi.org/10.1016/j.biopha.2022.113651.

[293] Ni Y, Liu M, Yu H, Chen Y, Liu Y, Chen S, Ruan J, Da A, Zhang Y, Wang T, Desmethylbellidifolin From Gentianella acuta Ameliorate TNBS-Induced Ulcerative Colitis Through Antispasmodic Effect and Anti-Inflammation, Frontiers in Pharmacology 10 (2019) 1104, https://doi.org/10.3389/fphar.2019.01104.

[294] Wu J, Wu Y, Chen Y, Liu M, Yu H, Zhang Y, Wang T, Desmethylbellidifolin Attenuates Dextran Sulfate Sodium-Induced Colitis: Impact on Intestinal Barrier, Intestinal Inflammation and Gut Microbiota, Planta Med 88 (2022) 559-569, https://doi.org/10.1055/a-1506-3476.

[295] Zhang WF, Yang Y, Su X, Xu DY, Yan YL, Gao Q, Duan MH, Deoxyschizandrin suppresses dss-induced ulcerative colitis in mice, Saudi J Gastroenterol 22 (2016) 448-455, https://doi.org/10.4103/1319-3767.195552.

[296] Yu S, Qian HH, Deoxyschizandrin treats mice with ulcerative colitis possibly via the TLR4/NF-κB signaling pathway, American journal of translational research 13

(2021) 3856-3863,
[297] X. ZY, Liu P, Zhang YQ, Jiang H, Luan HF, Xu YM, Zhang YB, Li RY, Demethyleneberberine blocked the maturation of IL-1β in inflammation by inhibiting TLR4-mitochondria signaling, International immunopharmacology 113 (2022) 109319,
[298] Zhou Q, Zhang WX, He ZQ, Wu BS, Shen ZF, Shang HT, Chen T, Wang Q, Chen YG, Han ST, The Possible Anti-Inflammatory Effect of Dehydrocostus Lactone on DSS-Induced Colitis in Mice, Evid Based Complement Alternat Med 2020 (2020) 5659738, https://doi.org/10.1155/2020/5659738.
[299] Shen J, L., Li N, Zhang X, Daidzein Ameliorates Dextran Sulfate Sodium Induced Experimental Colitis in Mice by Regulating NF-κB Signaling, J. Environ. Pathol. Toxicol. Oncol. 38 (2019) 29–39, https://doi.org/10.1615/JEnvironPatholToxicolOncol.2018027531.
[300] Satoh Y, Ishiguro Y, Sakuraba H, Kawaguchi S, Hiraga H, Fukuda S, Nakane A, Cyclosporine regulates intestinal epithelial apoptosis via TGF-beta-related signaling, American Journal of Physiology. Gastrointestinal and Liver Physiology 297 (2009) G514-519, https://doi.org/10.1152/ajpgi.90608.2008.
[301] Ota S, Sakuraba H, Hiraga H, Yoshida S, Satake M, Akemoto Y, Tanaka N, Watanabe R, Takato M, Murai Y, Ueno K, Niioka T, Hayakari M, Ishiguro Y, Fukuda S, Cyclosporine protects from intestinal epithelial injury by modulating butyrate uptake via upregulation of membrane monocarboxylate transporter 1 levels, Biochemistry and Biophysics Reports 24 (2020) 100811, https://doi.org/10.1016/j.bbrep.2020.100811.
[302] Bagalagel A, Diri R, Noor A, Almasri D, Bakhsh HT, Kutbi HI, Al-Gayyar MMH, The therapeutic effects of cycloastragenol in ulcerative colitis by modulating SphK/MIP-1α/miR-143 signalling, Basic & Clinical Pharmacology & Toxicology 131 (2022) 406-419, https://doi.org/10.1111/bcpt.13788.
[303] Chen YL, Chen DL, Zhou CH, Cao XC, He J, Correlation of Macrophages with Inflammatory Reaction in Ulcerative Colitis and Influence of Curcumin on Macrophage Chemotaxis, Alternative Therapies in Health and Medicine 29 (2023) 97-103,
[304] Wang HY, Ge W, Liu SQ, Long J, Jiang QQ, Zhou W, Zuo ZY, Liu DY, Zhao HM, Zhong YB, Curcumin Inhibits T Follicular Helper Cell Differentiation in Mice with Dextran Sulfate Sodium (DSS)-Induced Colitis, Am J Chin Med 50 (2022) 275-293, https://doi.org/10.1142/S0192415X22500100.
[305] Kang ZP, Wang MX, Wu TT, Liu DY, Wang HY, Long J, Zhao HM, Zhong YB, Curcumin Alleviated Dextran Sulfate Sodium-Induced Colitis by Regulating M1/M2 Macrophage Polarization and TLRs Signaling Pathway, Evid Based Complement Alternat Med 2021 (2021) 3334994, https://doi.org/10.1155/2021/3334994.
[306] Deguchi Y, Andoh A, Inatomi O, Yagi Y, Bamba S, Araki Y, Hata K, Tsujikawa T, Fujiyama Y, Curcumin prevents the development of dextran sulfate Sodium (DSS)-induced experimental colitis, Dig Dis Sci 52 (2007) 2993-2998, https://doi.org/10.1007/s10620-006-9138-9.
[307] Li CP, Li JH, He SY, Chen O, Shi L, Effect of curcumin on p38MAPK expression in DSS-induced murine ulcerative colitis, Genet Mol Res 14 (2015) 3450-3458, https://doi.org/10.4238/2015.April.15.8.
[308] Topcu-Tarladacalisir Y, Akpolat M, Uz YH, Kizilay G, Sapmaz-Metin M, Cerkezkayabekir A, Omurlu IK, Effects of curcumin on apoptosis and oxidoinflammatory regulation in a rat model of acetic acid-induced colitis: the roles of c-Jun N-terminal kinase and p38 mitogen-activated protein kinase, J Med Food 16 (2013) 296-305,

https://doi.org/10.1089/jmf.2012.2550.

[309] Zhang H, Lang W, Li S, Xu C, Wang X, Li Y, Zhang Z, Wu T, Feng M, Corynoline ameliorates dextran sulfate sodium-induced colitis in mice by modulating Nrf2/NF-κB pathway, Immunopharmacology and Immunotoxicology 45 (2022) 26-34, https://doi.org/10.1080/08923973.2022.2112218.

[310] Wang ZJ, Chen LH, Xu J, Xu QX, Xu W, Yang XW, Corylin ameliorates chronic ulcerative colitis via regulating the gut-brain axis and promoting 5-hydroxytryptophan production in the colon, Phytomedicine 110 (2023) 154651, https://doi.org/10.1016/j.phymed.2023.154651.

[311] Wang Y, Liu J, Huang Z, Li Y, Liang Y, Luo C, Ni C, Xie J, Su Z, Chen J, Li C, Coptisine ameliorates DSS-induced ulcerative colitis via improving intestinal barrier dysfunction and suppressing inflammatory response, Eur J Pharmacol 896 (2021) 173912, https://doi.org/10.1016/j.ejphar.2021.173912.

[312] Li MY, Zhang ZH, Wang Z, Zuo HX, Wang JY, Xing Y, Jin CH, Xu GH, Piao LX, Ma J, Jin X, Convallatoxin protects against dextran sulfate sodium-induced experimental colitis in mice by inhibiting NF-kappaB signaling through activation of PPARgamma, Pharmacol Res 147 (2019) 104355, https://doi.org/10.1016/j.phrs.2019.104355.

[313] Cai B, Zhou MH, Huang HL, Zhou AC, Chu ZD, Huang XD, Li CW, Protective effects of citrulline supplementation in ulcerative colitis rats, PLoS One 15 (2020) e0240883, https://doi.org/10.1371/journal.pone.0240883.

[314] Yang YX, Yuan Y, Xia B, Cinnamtannin D1 ameliorates DSS-induced colitis by preventing Th17/Treg imbalance through activation of the AMPK/mTOR pathway, Allergol Immunopathol (Madr) 50 (2022) 153-161, https://doi.org/10.15586/aei.v50i5.654.

[315] Qu S, Shen Y, Wang M, Wang X, Yang Y, Suppression of miR-21 and miR-155 of macrophage by cinnamaldehyde ameliorates ulcerative colitis, Int Immunopharmacol 67 (2019) 22-34, https://doi.org/10.1016/j.intimp.2018.11.045.

[316] Tan X, Wen Y, Han Z, Su X, Peng J, Chen F, Wang Y, Wang T, Wang C, Ma K, Cinnamaldehyde Ameliorates Dextran Sulfate Sodium-Induced Colitis in Mice by Modulating TLR4/NF-kappaB Signaling Pathway and NLRP3 Inflammasome Activation, Chemistry & Biodiversity 20 (2023) e202200089, https://doi.org/10.1002/cbdv.202200089.

[317] Ma KL, Han ZJ, Pan M, Chen ML, Ge YZ, Shao J, Wu DQ, Wang TM, Yan GM, Wang CZ, Therapeutic effect of cinnamaldehyde on ulcerative colitis in mice induced bydextran sulfate sodium with Candida albicans colonization and its effect ondectin-1/TLＲs/NF-κB signaling pathway, China J. Chin. Mater. Med. 45 (2020) 3211-3219, https://doi.org/10.19540/j.cnki.cjcmm.20200421.401.

[318] Qu SL, Chen L, Wen XS, Zuo JP, Wang XY, Lu ZJ, Yang YF, Suppression of Th17 cell differentiation via sphingosine-1-phosphate receptor 2 by cinnamaldehyde can ameliorate ulcerative colitis, Biomed Pharmacother 134 (2021) 111116, https://doi.org/10.1016/j.biopha.2020.111116.

[319] Kim D, Kim S, Kim M, Jeon Y, Um J, Hong S, The Therapeutic Effect of Chelidonic Acid on Ulcerative Colitis, Biological & Pharmaceutical Bulletin 35 (2012) 666-671, https://doi.org/10.1248/bpb.35.666.

[320] Li M, Guo W, Dong Y, Wang W, Tian C, Zhang Z, Yu T, Zhou H, Gui Y, Xue K, Li J, Jiang F, Sarapultsev A, Wang H, Zhang G, Luo S, Fan H, Hu D, Beneficial Effects of Celastrol on Immune Balance by Modulating Gut Microbiota in Experimental Ulcerative Colitis Mice, Genomics Proteomics Bioinformatics 20 (2022) 288-303, https://doi.org/10.1016/j.gpb.2022.05.002.

[321] Jia Z, Xu C, Shen J, Xia T, Yang J, He Y, The natural compound celastrol inhibits necroptosis and alleviates ulcerative colitis in mice, Int Immunopharmacol 29 (2015) 552-559, https://doi.org/10.1016/j.intimp.2015.09.029.

[322] Shaker ME, Ashamallah SA, Houssen ME, Celastrol ameliorates murine colitis via modulating oxidative stress, inflammatory cytokines and intestinal homeostasis, Chem Biol Interact 210 (2014) 26-33, https://doi.org/10.1016/j.cbi.2013.12.007.

[323] Niu X, Zhang H, Li W, Wang Y, Mu Q, Wang X, He Z, Yao H, Protective effect of cavidine on acetic acid-induced murine colitis via regulating antioxidant, cytokine profile and NF-kappaB signal transduction pathways, Chem Biol Interact 239 (2015) 34-45, https://doi.org/10.1016/j.cbi.2015.06.026.

[324] Lucena AMM, Souza CRM, Jales JT, Guedes PMM, de Miranda GEC, de Moura AMA, Araujo-Junior JX, Nascimento GJ, Scortecci KC, Santos BVO, Souto JT, The Bisindole Alkaloid Caulerpin, from Seaweeds of the Genus Caulerpa, Attenuated Colon Damage in Murine Colitis Model, Mar Drugs 16 (2018) 318, https://doi.org/10.3390/md16090318.

[325] Kook SH, Choi KC, Cho SW, Cho HK, Lee KD, Lee JC, Catechin-7-O-beta-D-glucopyranoside isolated from the seed of Phaseolus calcaratus Roxburgh ameliorates experimental colitis in rats, Int Immunopharmacol 29 (2015) 521-527, https://doi.org/10.1016/j.intimp.2015.10.003.

[326] Ma J, Yin G, Lu Z, Xie P, Zhou H, Liu J, Yu L, Casticin prevents DSS induced ulcerative colitis in mice through inhibitions of NF-kappaB pathway and ROS signaling, Phytother Res 32 (2018) 1770-1783, https://doi.org/10.1002/ptr.6108.

[327] Xu X, Zhang G, Peng K, Gao Y, Wang J, Gao C, He C, Lu F, Carnosol Maintains Intestinal Barrier Function and Mucosal Immune Homeostasis in DSS-Induced Colitis, Frontiers in Nutrition 9 (2022) 894307, https://doi.org/10.3389/fnut.2022.894307.

[328] Yang N, Xia Z, Shao N, Li B, Xue L, Peng Y, Zhi F, Yang Y, Carnosic acid prevents dextran sulfate sodium-induced acute colitis associated with the regulation of the Keap1/Nrf2 pathway, Sci Rep 7 (2017) 11036, https://doi.org/10.1038/s41598-017-11408-5.

[329] Ali AA, Abd Al Haleem EN, Khaleel SA, Sallam AS, Protective effect of cardamonin against acetic acid-induced ulcerative colitis in rats, Pharmacol Rep 69 (2017) 268-275, https://doi.org/10.1016/j.pharep.2016.11.002.

[330] Wan H, Chen XY, Zhang F, Chen J, Chu F, Sellers ZM, Xu F, Dong H, Capsaicin inhibits intestinal Cl(-) secretion and promotes Na(+) absorption by blocking TRPV4 channels in healthy and colitic mice, J Biol Chem 298 (2022) 101847, https://doi.org/10.1016/j.jbc.2022.101847.

[331] Lian YZ, Chang CC, Chen YS, Tinkov AA, Skalny AV, Chao JC, Lycium barbarum polysaccharides and capsaicin modulate inflammatory cytokines and colonic microbiota in colitis rats induced by dextran sulfate sodium, J Clin Biochem Nutr 71 (2022) 229-237, https://doi.org/10.3164/jcbn.21-174.

[332] Pagano E, Romano B, Iannotti FA, Parisi OA, D'Armiento M, Pignatiello S, Coretti L, Lucafo M, Venneri T, Stocco G, Lembo F, Orlando P, Capasso R, Di Marzo V, Izzo AA, Borrelli F, The non-euphoric phytocannabinoid cannabidivarin counteracts intestinal inflammation in mice and cytokine expression in biopsies from UC pediatric patients, Pharmacol Res 149 (2019) 104464, https://doi.org/10.1016/j.phrs.2019.104464.

[333] Wang Y, Liu K, Qi Z, Chen T, Yu W, Jiang Y, Li G, Xiao H, Therapeutic Mechanism and Effect of Camptothecin on Dextran Sodium Sulfate-Induced Ulcerative Colitis in Mice, J Immunol Res 2021 (2021) 5556659, https://doi.org/10.1155/2021/5556659.

[334] Khan MN, Lane ME, McCarron PA, Tambuwala MM, Caffeic acid phenethyl ester is protective in experimental ulcerative colitis via reduction in levels of pro-inflammatory mediators and enhancement of epithelial barrier function, Inflammopharmacology 26 (2018) 561-569, https://doi.org/10.1007/s10787-017-0364-x.

[335] Pandurangan AK, Mohebali N, Hasanpourghadi M, Esa NM, Caffeic Acid Phenethyl Ester Attenuates Dextran Sulfate Sodium-Induced Ulcerative Colitis Through Modulation of NF-kappaB and Cell Adhesion Molecules, Applied Biochemistry and Biotechnology 194 (2022) 1091-1104, https://doi.org/10.1007/s12010-021-03788-2.

[336] Zhang Z, Wu XY, Cao SY, Wang L, Wang D, Yang H, Feng YM, Wang SL, Li L, Caffeic acid ameliorates colitis in association with increased Akkermansia population in the gut microbiota of mice, Oncotarget 7 (2016) 31790-31799, https://doi.org/10.18632/oncotarget.9306.

[337] Xiang C, Liu M, Lu Q, Fan C, Lu H, Feng C, Yang X, Li H, Tang W, Blockade of TLRs-triggered macrophage activation by caffeic acid exerted protective effects on experimental ulcerative colitis, Cell Immunol 365 (2021) 104364, https://doi.org/10.1016/j.cellimm.2021.104364.

[338] Malago JJ, Sangu CL, Intraperitoneal administration of butyrate prevents the severity of acetic acid colitis in rats, J Zhejiang Univ Sci B 16 (2015) 224-234, https://doi.org/10.1631/jzus.B1400191.

[339] Zhang M, Zhou Q, Dorfman RG, Huang X, Fan T, Zhang H, Zhang J, Yu C, Butyrate inhibits interleukin-17 and generates Tregs to ameliorate colorectal colitis in rats, BMC Gastroenterol 16 (2016) 84, https://doi.org/10.1186/s12876-016-0500-x.

[340] Xiao T, Zhang P, Feng T, Lu K, Wang X, Zhou S, Qiang Y, Butyrate functions in concert with myeloid-derived suppressor cells recruited by CCR9 to alleviate DSS-induced murine colitis, Int Immunopharmacol 99 (2021) 108034, https://doi.org/10.1016/j.intimp.2021.108034.

[341] Li R, Chen C, Liu B, Shi W, Shimizu K, Zhang C, Bryodulcosigenin a natural cucurbitane-type triterpenoid attenuates dextran sulfate sodium (DSS)-induced colitis in mice, Phytomedicine 94 (2022) 153814, https://doi.org/10.1016/j.phymed.2021.153814.

[342] Dou YX, Zhou JT, Wang TT, Huang YF, Chen VP, Xie YL, Lin ZX, Gao JS, Su ZR, Zeng HF, Self-nanoemulsifying drug delivery system of bruceine D: a new approach for anti-ulcerative colitis, Int J Nanomedicine 13 (2018) 5887-5907, https://doi.org/10.2147/IJN.S174146.

[343] Pandurangan AK, Mohebali N, Hasanpourghadi M, Looi CY, Mustafa MR, Mohd Esa N, Boldine suppresses dextran sulfate sodium-induced mouse experimental colitis: NF-kappaB and IL-6/STAT3 as potential targets, Biofactors 42 (2016) 247-258, https://doi.org/10.1002/biof.1267.

[344] Zhang H, Wang Y, Su Y, Fang X, Guo W, The alleviating effect and mechanism of Bilobalide on ulcerative colitis, Food Funct 12 (2021) 6226-6239,

https://doi.org/10.1039/d1fo01266e.
[345] Prados ME, Garcia-Martin A, Unciti-Broceta JD, Palomares B, Collado JA, Minassi A, Calzado MA, Appendino G, Munoz E, Betulinic acid hydroxamate prevents colonic inflammation and fibrosis in murine models of inflammatory bowel disease, Acta Pharmacol Sin 42 (2021) 1124-1138, https://doi.org/10.1038/s41401-020-0497-0.
[346] El-Sherbiny M, Eisa NH, Abo El-Magd NF, Elsherbiny NM, Said E, Khodir AE, Anti-inflammatory/anti-apoptotic impact of betulin attenuates experimentally induced ulcerative colitis: An insight into TLR4/NF-kB/caspase signalling modulation, Environ Toxicol Pharmacol 88 (2021) 103750, https://doi.org/10.1016/j.etap.2021.103750.
[347] Chen L, Liu D, Mao M, Liu W, Wang Y, Liang Y, Cao W, Zhong X, Betaine Ameliorates Acute Sever Ulcerative Colitis by Inhibiting Oxidative Stress Induced Inflammatory Pyroptosis, Mol Nutr Food Res 66 (2022) e2200341, https://doi.org/10.1002/mnfr.202200341.
[348] Lopes de Oliveira GA, Alarcon de la Lastra C, Rosillo MA, Castejon Martinez ML, Sanchez-Hidalgo M, Rolim Medeiros JV, Villegas I, Preventive effect of bergenin against the development of TNBS-induced acute colitis in rats is associated with inflammatory mediators inhibition and NLRP3/ASC inflammasome signaling pathways, Chem Biol Interact 297 (2019) 25-33, https://doi.org/10.1016/j.cbi.2018.10.020.
[349] Wang K, Li YF, Lv Q, Li XM, Dai Y, Wei ZF, Bergenin, Acting as an Agonist of PPARgamma, Ameliorates Experimental Colitis in Mice through Improving Expression of SIRT1, and Therefore Inhibiting NF-kappaB-Mediated Macrophage Activation, Front Pharmacol 8 (2017) 981, https://doi.org/10.3389/fphar.2017.00981.
[350] Yu XT, Xu YF, Huang YF, Qu C, Xu LQ, Su ZR, Zeng HF, Zheng L, Yi TG, Li HL, Chen JP, Zhang XJ, Berberrubine attenuates mucosal lesions and inflammation in dextran sodium sulfate-induced colitis in mice, Plos One 13 (2018) e0194069, https://doi.org/10.1371/journal.pone.0194069.
[351] Zhu L, Gu PQ, Shen H, Protective effects of berberine hydrochloride on DSS-induced ulcerative colitis in rats, International Immunopharmacology 68 (2019) 242-251, https://doi.org/10.1016/j.intimp.2018.12.036.
[352] Jia L, Xue K, Liu J, Habotta OA, Hu L, Abdel Moneim AE, Anticolitic Effect of Berberine in Rat Experimental Model: Impact of PGE2/p38 MAPK Pathways, Mediators Inflamm 2020 (2020) 9419085, https://doi.org/10.1155/2020/9419085.
[353] Shen Y, Liu YC, L. WZ, L. RX, S. L, Y. NS, H. ZJ, Effect of Berberine from Coptis chinensis on Apoptosis of Intestinal Epithelial Cells in a Mouse Model of Ulcerative Colitis: Role of Endoplasmic Reticulum Stress, Evid Based Complement Alternat Med 2020 (2020) 3784671, https://doi.org/10.1155/2020/3784671.
[354] Jia D, Dou Y, Li Z, Zhou X, Gao Y, Chen K, Cong W, Ma M, Wu Z, Li W, Design, synthesis and evaluation of a baicalin and berberine hybrid compound as therapeutic agent for ulcerative colitis, Bioorganic & Medicinal Chemistry 28 (2020) 115697, https://doi.org/10.1016/j.bmc.2020.115697.
[355] Li H, Feng C, Fan C, Yang Y, Yang X, Lu H, Lu Q, Zhu F, Xiang C, Zhang Z, He P, Zuo J, Tang W, Intervention of oncostatin M-driven mucosal inflammation by berberine exerts therapeutic property in chronic ulcerative colitis, Cell Death Dis 11 (2020) 271, https://doi.org/10.1038/s41419-020-2470-8.
[356] Jiang Y, Zhao L, Chen Q, Zhou LH, Exploring the Mechanism of Berberine Intervention in Ulcerative Colitis from the Perspective of Inflammation and Immunity

Based on Systemic Pharmacology, Evidence-Based Complementary and Alternative Medicine 2021 (2021) 9970240, https://doi.org/10.1155/2021/9970240.
[357] Deng JP, Wu ZC, Zhao ZL, Wu CX, Yuan M, Su ZQ, Wang YF, Wang ZP, Berberine-Loaded Nanostructured Lipid Carriers Enhance the Treatment of Ulcerative Colitis, International Journal of Nanomedicine 15 (2020) 3937-3951, https://doi.org/10.2147/ijn.S247406.
[358] Zhai LX, Huang T, Xiao HT, Wu PG, Lin CY, Ning ZW, Zhao L, Kwan H, Hu XJ, Wong HLX, Li XQ, Bian ZX, Berberine Suppresses Colonic Inflammation in Dextran Sulfate Sodium-Induced Murine Colitis Through Inhibition of Cytosolic Phospholipase A2 Activity, Frontiers in Pharmacology 11 (2020) 576496, https://doi.org/10.3389/fphar.2020.576496.
[359] Cui H, Cai Y, Wang L, Jia B, Li J, Zhao S, Chu X, Lin J, Zhang X, Bian Y, Zhuang P, Berberine Regulates Treg/Th17 Balance to Treat Ulcerative Colitis Through Modulating the Gut Microbiota in the Colon, Front Pharmacol 9 (2018) 571, https://doi.org/10.3389/fphar.2018.00571.
[360] Li Q, Qu X, Pang X, Song Y, Chen L, Xiao Q, Sun L, Wang X, Zhang H, Qi D, Wang Z, Berberine Protects Mice Against Dextran Sulfate Sodium-Induced Colitis by Activating mTORC1 Pathway, Front Pharmacol 10 (2019) 786, https://doi.org/10.3389/fphar.2019.00786.
[361] Yang T, Ma X, Wang RL, Liu HH, Wei SZ, Jing MY, Li HT, Zhao YL, Berberine inhibits IFN-γ signaling pathway in DSS-induced ulcerative colitis, Saudi Pharmaceutical Journal 30 (2022) 764-778, https://doi.org/10.1016/j.jsps.2022.03.015.
[362] Xu X, Li W, Yu Z, Zhang L, Duo T, Zhao Y, Qin W, Yang W, Ma L, Berberine Ameliorates Dextran Sulfate Sodium-Induced Ulcerative Colitis and Inhibits the Secretion of Gut Lysozyme via Promoting Autophagy, Metabolites 12 (2022) 676, https://doi.org/10.3390/metabo12080676.
[363] Liao ZQ, Xie YZ, Zhou BJ, Zou B, R., Xiao D, Liu W, Cai Y, Liu DL, Liao QF, Xie ZY, Berberine ameliorates colonic damage accompanied with the modulation of dysfunctional bacteria and functions in ulcerative colitis rats, Applied Microbiology and Biotechnology 104 (2020) 1737-1749, https://doi.org/10.1007/s00253-019-10307-1.
[364] (!!! INVALID CITATION !!!)
[365] Gai L, Chu L, Xia R, Chen Q, Sun X, Barbaloin Attenuates Mucosal Damage in Experimental Models of Rat Colitis by Regulating Inflammation and the AMPK Signaling Pathway, Medical Science Monitor 25 (2019) 10045-10056, https://doi.org/10.12659/MSM.918935.
[366] Cui L, Feng L, Zhang ZH, Jia XB, The anti-inflammation effect of baicalin on experimental colitis through inhibiting TLR4/NF-kappaB pathway activation, Int Immunopharmacol 23 (2014) 294-303, https://doi.org/10.1016/j.intimp.2014.09.005.
[367] Yao J, Cao X, Zhang R, Li YX, Xu ZL, Zhang DG, Wang LS, Wang JY, Protective Effect of Baicalin Against Experimental Colitis via Suppression of Oxidant Stress and Apoptosis, Pharmacogn Mag 12 (2016) 225-234, https://doi.org/10.4103/0973-1296.186342.
[368] Zhang CL, Zhang S, He WX, Lu JL, Xu YJ, Yang JY, Liu D, Baicalin may alleviate inflammatory infiltration in dextran sodium sulfate-induced chronic ulcerative colitis via inhibiting IL-33 expression, Life Sci 186 (2017) 125-132, https://doi.org/10.1016/j.lfs.2017.08.010.

[369] Dai SX, Zou Y, Feng YL, Liu HB, Zheng XB, Baicalin down-regulates the expression of macrophage migration inhibitory factor (MIF) effectively for rats with ulcerative colitis, Phytother Res 26 (2012) 498-504, https://doi.org/10.1002/ptr.3581.

[370] Feng JS, Guo CC, Zhu YZ, Pang LP, Yang Z, Zou Y, Zheng XB, Baicalin down regulates the expression of TLR4 and NFkB-p65 in colon tissue in mice with colitis induced by dextran sulfate sodium, International Journal of Clinical and Experimental Medicine 7 (2014) 4063-4072,

[371] Zou Y, Dai SX, Chi HG, Li T, He ZW, Wang J, Ye CG, Huang GL, Zhao B, Li WY, Wan Z, Feng JS, Zheng XB, Baicalin attenuates TNBS-induced colitis in rats by modulating the Th17/Treg paradigm, Arch Pharm Res 38 (2015) 1873-1887, https://doi.org/10.1007/s12272-014-0486-2.

[372] Shen J, Cheng J, Zhu S, Zhao J, Ye Q, Xu Y, Dong H, Zheng X, Regulating effect of baicalin on IKK/IKB/NF-kB signaling pathway and apoptosis-related proteins in rats with ulcerative colitis, Int Immunopharmacol 73 (2019) 193-200, https://doi.org/10.1016/j.intimp.2019.04.052.

[373] Zhu L, Xu LZ, Zhao S, Shen ZF, Shen H, Zhan LB, Protective effect of baicalin on the regulation of Treg/Th17 balance, gut microbiota and short-chain fatty acids in rats with ulcerative colitis, Appl Microbiol Biotechnol 104 (2020) 5449-5460, https://doi.org/10.1007/s00253-020-10527-w.

[374] Liang S, Deng X, Lei L, Zheng Y, Ai J, Chen L, Xiong H, Mei Z, Cheng YC, Ren Y, The Comparative Study of the Therapeutic Effects and Mechanism of Baicalin, Baicalein, and Their Combination on Ulcerative Colitis Rat, Front Pharmacol 10 (2019) 1466, https://doi.org/10.3389/fphar.2019.01466.

[375] Liu C, Li Y, Chen Y, Huang S, Wang X, Luo S, Su Y, Zhou L, Luo X, Baicalein Restores the Balance of Th17/Treg Cells via Aryl Hydrocarbon Receptor to Attenuate Colitis, Mediators Inflamm 2020 (2020) 5918587, https://doi.org/10.1155/2020/5918587.

[376] Huang B, Wang L, Liu M, Wu X, Lu Q, Liu R, The underlying mechanism of A-type procyanidins from peanut skin on DSS-induced ulcerative colitis mice by regulating gut microbiota and metabolism, Journal of Food Biochemistry 46 (2022) e14103, https://doi.org/10.1111/jfbc.14103.

[377] Wang N, Chen W, Cui C, Zheng Y, Yu Q, Ren H, Liu Z, Xu C, Zhang G, The Peanut Skin Procyanidins Attenuate DSS-Induced Ulcerative Colitis in C57BL/6 Mice, Antioxidants (Basel) 11 (2022) 2098, https://doi.org/10.3390/antiox11112098.

[378] Qu L, Lin X, Liu C, Ke C, Zhou Z, Xu K, Cao G, Liu Y, Atractylodin Attenuates Dextran Sulfate Sodium-Induced Colitis by Alleviating Gut Microbiota Dysbiosis and Inhibiting Inflammatory Response Through the MAPK Pathway, Front Pharmacol 12 (2021) 665376, https://doi.org/10.3389/fphar.2021.665376.

[379] Qu L, Shi K, Xu J, Liu C, Ke C, Zhan X, Xu K, Liu Y, Atractylenolide-1 targets SPHK1 and B4GALT2 to regulate intestinal metabolism and flora composition to improve inflammation in mice with colitis, Phytomedicine 98 (2022) 153945, https://doi.org/10.1016/j.phymed.2022.153945.

[380] Han J, Li W, Shi G, Huang Y, Sun X, Sun N, Jiang D, Atractylenolide III Improves Mitochondrial Function and Protects Against Ulcerative Colitis by Activating AMPK/SIRT1/PGC-1alpha, Mediators Inflamm 2022 (2022) 9129984, https://doi.org/10.1155/2022/9129984.

[381] Zhong Y, Liu W, Xiong Y, Li Y, Wan Q, Zhou W, Zhao H, Xiao Q, Liu D, Astragaloside Ⅳ alleviates ulcerative colitis by regulating the balance of Th17/Treg cells, Phytomedicine 104 (2022) 154287, https://doi.org/10.1016/j.phymed.2022.154287.

[382] Xu SY, Hu XD, Yang ZL, Liu A, He YH, Lu HY, Effects of astragalosideⅣ on inflammatory response and percentage ofperipheral blood Th17 cells in mice with ulcerative colitis, China J. Chin. Mater. Med. 47 (2022) 469-475, https://doi.org/10.19540/j.cnki.cjcmm.20210827.401.

[383] Wu S, Chen Z, Astragaloside IV alleviates the symptoms of experimental ulcerative colitis in vitro and in vivo, Exp Ther Med 18 (2019) 2877-2884, https://doi.org/10.3892/etm.2019.7907.

[384] Qiao CX, Wan JE, Zhang LZ, Luo B, Liu PL, Di AT, Gao HR, Sun XM, Zhao G, Astragaloside II alleviates the symptoms of experimental ulcerative colitis in vitro and in vivo, American Journal of Translational Research 11 (2019) 7074-7083,

[385] Peng L, Gao X, Nie L, Xie J, Dai T, Shi C, Tao L, Wang Y, Tian Y, Sheng J, Astragalin Attenuates Dextran Sulfate Sodium (DSS)-Induced Acute Experimental Colitis by Alleviating Gut Microbiota Dysbiosis and Inhibiting NF-kappaB Activation in Mice, Front Immunol 11 (2020) 2058, https://doi.org/10.3389/fimmu.2020.02058.

[386] Chen YE, Xu SJ, Lu YY, Chen SX, Du XH, Hou SZ, Huang HY, Liang J, Asperuloside suppressing oxidative stress and inflammation in DSS-induced chronic colitis and RAW 264.7 macrophages via Nrf2/HO-1 and NF-kappaB pathways, Chemico-Biological Interactions 344 (2021) 109512, https://doi.org/10.1016/j.cbi.2021.109512.

[387] Hu X, He X, Peng C, He Y, Wang C, Tang W, Chen H, Feng Y, Liu D, Li T, He L, Improvement of Ulcerative Colitis by Aspartate via RIPK Pathway Modulation and Gut Microbiota Composition in Mice, Nutrients 14 (2022) 3707, https://doi.org/10.3390/nu14183707.

[388] Yin S, Li L, Tao Y, Yu J, Wei S, Liu M, Li J, The Inhibitory Effect of Artesunate on Excessive Endoplasmic Reticulum Stress Alleviates Experimental Colitis in Mice, Front Pharmacol 12 (2021) 629798, https://doi.org/10.3389/fphar.2021.629798.

[389] Yin S, Yang H, Tao Y, Wei S, Li L, Liu M, Li J, Artesunate ameliorates DSS-induced ulcerative colitis by protecting intestinal barrier and inhibiting inflammatory response, Inflammation 43 (2020) 765-776, https://doi.org/10.1007/s10753-019-01164-1.

[390] Yang ZB, Qiu LZ, Chen Q, Lin JD, Artesunate alleviates the inflammatory response of ulcerative colitis by regulating the expression of miR-155, Pharm Biol 59 (2021) 97-105, https://doi.org/10.1080/13880209.2020.1867196.

[391] Chen YX, Zhang XQ, Yu CG, Huang SL, Xie Y, Dou XT, Liu WJ, Zou XP, Artesunate exerts protective effects against ulcerative colitis via suppressing Toll-like receptor 4 and its downstream nuclear factor-kappaB signaling pathways, Mol Med Rep 20 (2019) 1321-1332, https://doi.org/10.3892/mmr.2019.10345.

[392] Jia X, Gao Y, Liu L, Guo Y, Wang J, Ma H, Zhao R, Li B, Du Y, Yang Q, Artemisinin Alleviates Intestinal Inflammation and Metabolic Disturbance in Ulcerative Colitis Rats Induced by DSS, Evid Based Complement Alternat Med 2022 (2022) 6211215, https://doi.org/10.1155/2022/6211215.

[393] Li C, Zhang W, Wu X, Cai Q, Tan Z, Hong Z, Huang S, Yuan Y, Yao L, Zhang L, Aromatic-turmerone ameliorates DSS-induced ulcerative colitis via modulating gut microbiota in mice, Inflammopharmacology 30 (2022) 1283-1294, https://doi.org/10.1007/s10787-022-01007-w.

[394] Wu X, Yang Y, Dou Y, Ye J, Bian D, Wei Z, Tong B, Kong L, Xia Y, Dai Y, Arctigenin but not arctiin acts as the major effective constituent of Arctium lappa L. fruit for attenuating colonic inflammatory response induced by dextran sulfate sodium in mice, Int Immunopharmacol 23 (2014) 505-515,

https://doi.org/10.1016/j.intimp.2014.09.026.

[395] Zhang C, Zhu H, Jie H, Ding H, Sun H, Arbutin ameliorated ulcerative colitis of mice induced by dextran sodium sulfate (DSS), Bioengineered 12 (2021) 11707-11715, https://doi.org/10.1080/21655979.2021.2005746.

[396] Wei DD, Lin XH, Wang HC, Wang B, Bai CY, Wang YQ, Li GE, Ren XQ, Apocynin relieves inflammation in dextran sulfate sodium-induced ulcerative colitis mice: the role of NOXs-ROS-p38MAPK pathway, Acta Physiologica Sinica 67 (2015) 74-82, https://doi.org/10.13294/j.aps.2015.0008.

[397] Ganjare AB, Nirmal SA, Patil AN, Use of apigenin from Cordia dichotoma in the treatment of colitis, Fitoterapia 82 (2011) 1052-1056, https://doi.org/10.1016/j.fitote.2011.06.008.

[398] Marquez-Flores YK, Villegas I, Cardeno A, Rosillo MA, Alarcon-de-la-Lastra C, Apigenin supplementation protects the development of dextran sulfate sodium-induced murine experimental colitis by inhibiting canonical and non-canonical inflammasome signaling pathways, J Nutr Biochem 30 (2016) 143-152, https://doi.org/10.1016/j.jnutbio.2015.12.002.

[399] Fu R, Wang L, Meng Y, Xue W, Liang J, Peng Z, Meng J, Zhang M, Apigenin remodels the gut microbiota to ameliorate ulcerative colitis, Frontiers in Nutrition 9 (2022) 1062961, https://doi.org/10.3389/fnut.2022.1062961.

[400] Shibrya EE, Rashed RR, Abd El Fattah MA, El-Ghazaly MA, Kenawy SA, Apigenin and Exposure to Low Dose Gamma Radiation Ameliorate Acetic Acid-Induced Ulcerative Colitis in Rats, Dose-Response 21 (2023) 15593258231155787, https://doi.org/10.1177/15593258231155787.

[401] Han Q, Deng LR, Zou M, Tang HZ, Huang CY, Chen FJ, Tomlinson B, Li YH, Anemoside B4 protects against chronic relapsing colitis in mice by modulating inflammatory response, colonic transcriptome and the gut microbiota, Phytomedicine 106 (2022) 154416, https://doi.org/10.1016/j.phymed.2022.154416.

[402] Ma H, Zhou M, Duan W, Chen L, Wang L, Liu P, Anemoside B4 prevents acute ulcerative colitis through inhibiting of TLR4/NF-kappaB/MAPK signaling pathway, Int Immunopharmacol 87 (2020) 106794, https://doi.org/10.1016/j.intimp.2020.106794.

[403] Zhang Y, Zha Z, Shen W, Li D, Kang N, Chen Z, Liu Y, Xu G, Xu Q, Anemoside B4 ameliorates TNBS-induced colitis through S100A9/MAPK/NF-kappaB signaling pathway, Chin Med 16 (2021) 11, https://doi.org/10.1186/s13020-020-00410-1.

[404] Jiang L, Chi C, Yuan F, Lu M, Hu D, Wang L, Liu X, Anti-inflammatory effects of anemonin on acute ulcerative colitis via targeted regulation of protein kinase C-theta, Chin Med 17 (2022) 39, https://doi.org/10.1186/s13020-022-00599-3.

[405] Guan F, Luo H, Wu J, Li M, Chen L, Huang N, Wei G, Nie J, Chen B, Su Z, Zhang X, Liu Y, Andrographolide sodium bisulfite ameliorates dextran sulfate sodium-induced colitis and liver injury in mice via inhibiting macrophage proinflammatory polarization from the gut-liver axis, Int Immunopharmacol 110 (2022) 109007, https://doi.org/10.1016/j.intimp.2022.109007.

[406] Zhang L, Cao N, Wang Y, Wang Y, Wu C, Cheng X, Wang C, Improvement of Oxazolone-Induced Ulcerative Colitis in Rats Using Andrographolide, Molecules 25

(2019) 76, https://doi.org/10.3390/molecules25010076.
[407] Sakthivel KM, Guruvayoorappan C, Amentoflavone inhibits iNOS, COX-2 expression and modulates cytokine profile, NF-kappaB signal transduction pathways in rats with ulcerative colitis, Int Immunopharmacol 17 (2013) 907-916, https://doi.org/10.1016/j.intimp.2013.09.022.
[408] Tan Y, Zheng C, Effects of Alpinetin on Intestinal Barrier Function, Inflammation and Oxidative Stress in Dextran Sulfate Sodium-Induced Ulcerative Colitis Mice, Am J Med Sci 355 (2018) 377-386, https://doi.org/10.1016/j.amjms.2018.01.002.
[409] Miao Y, Lv Q, Qiao S, Yang L, Tao Y, Yan W, Wang P, Cao N, Dai Y, Wei Z, Alpinetin improves intestinal barrier homeostasis via regulating AhR/suv39h1/TSC2/mTORC1/autophagy pathway, Toxicol Appl Pharmacol 384 (2019) 114772, https://doi.org/10.1016/j.taap.2019.114772.
[410] Lv Q, Shi C, Qiao S, Cao N, Guan C, Dai Y, Wei Z, Alpinetin exerts anti-colitis efficacy by activating AhR, regulating miR-302/DNMT-1/CREB signals, and therefore promoting Treg differentiation, Cell Death & Disease 9 (2018) 890, https://doi.org/10.1038/s41419-018-0814-4.
[411] Shi L, Lin Q, Li X, Nie Y, Sun S, Deng X, Wang L, Lu J, Tang Y, Luo F, Alliin, a garlic organosulfur compound, ameliorates gut inflammation through MAPK-NF-kappaB/AP-1/STAT-1 inactivation and PPAR-gamma activation, Mol Nutr Food Res 61 (2017) 1601013, https://doi.org/10.1002/mnfr.201601013.
[412] Jiang H, Shi GF, Fang YX, Liu YQ, Wang Q, Zheng X, Zhang DJ, Zhang J, Yin ZQ, Aloin A prevents ulcerative colitis in mice by enhancing the intestinal barrier function via suppressing the Notch signaling pathway, Phytomedicine 106 (2022) 154403, https://doi.org/10.1016/j.phymed.2022.154403.
[413] Wang X, Su L, Tan J, Ding T, Yue Y, Albiflorin alleviates DSS-induced ulcerative colitis in mice by reducing inflammation and oxidative stress, Iran J Basic Med Sci 26 (2023) 48-56, https://doi.org/10.22038/IJBMS.2022.66678.14624.
[414] Hou YC, Chu CC, Ko TL, Yeh CL, Yeh SL, Effects of alanyl-glutamine dipeptide on the expression of colon-inflammatory mediators during the recovery phase of colitis induced by dextran sulfate sodium, Eur J Nutr 52 (2013) 1089-1098, https://doi.org/10.1007/s00394-012-0416-3.
[415] Chu CC, Hou YC, Pai MH, Chao CJ, Yeh SL, Pretreatment with alanyl-glutamine suppresses T-helper-cell-associated cytokine expression and reduces inflammatory responses in mice with acute DSS-induced colitis, J Nutr Biochem 23 (2012) 1092-1099, https://doi.org/10.1016/j.jnutbio.2011.06.002.
[416] Tian X, Peng Z, Luo S, Zhang S, Li B, Zhou C, Fan H, Aesculin protects against DSS-Induced colitis though activating PPARgamma and inhibiting NF-small ka, CyrillicB pathway, Eur J Pharmacol 857 (2019) 172453, https://doi.org/10.1016/j.ejphar.2019.172453.
[417] Guo W, Wang X, Liu F, Chen S, Wang S, Zhang Q, Yuan L, Du S, Acteoside alleviates dextran sulphate sodium-induced ulcerative colitis via regulation of the HO-1/HMGB1 signaling pathway, Mol Med Rep 26 (2022) 360, https://doi.org/10.3892/mmr.2022.12877.
[418] Kang OH, Kim DK, Cai XF, Kim YH, Lee YM, Attenuation of experimental murine colitis by acanthoic acid from Acanthopanax koreanum, Archives of Pharmacal Research 33 (2010) 87-93, https://doi.org/10.1007/s12272-010-2230-x.
[419] Ren J, Yue B, Wang H, Zhang B, Luo X, Yu Z, Zhang J, Ren Y, Mani S, Wang Z, Dou W, Acacetin Ameliorates Experimental Colitis in Mice via Inhibiting Macrophage

Inflammatory Response and Regulating the Composition of Gut Microbiota, Front Physiol 11 (2020) 577237, https://doi.org/10.3389/fphys.2020.577237.
[420] Cheng J, Ma X, Zhang H, Wu X, Li M, Ai G, Zhan R, Xie J, Su Z, Huang X, 8-Oxypalmatine, a novel oxidative metabolite of palmatine, exhibits superior anti-colitis effect via regulating Nrf2 and NLRP3 inflammasome, Biomed Pharmacother 153 (2022) 113335, https://doi.org/10.1016/j.biopha.2022.113335.
[421] Zhang F, Ma N, Gao YF, Sun LL, Zhang JG, Therapeutic Effects of 6-Gingerol, 8-Gingerol, and 10-Gingerol on Dextran Sulfate Sodium-Induced Acute Ulcerative Colitis in Rats, Phytother Res 31 (2017) 1427-1432, https://doi.org/10.1002/ptr.5871.
[422] Wei HL, Li JT, Chen ZG, Yan SG, Experimental study on effects of berberine combined with 6-shogaol onintestinal inflammation and flora in mice with ulcerative colitis, China J. Chin. Mater. Med. 47 (2022) 4418-4427, https://doi.org/10.19540/j.cnki.cjcmm.20220413.401.
[423] Rafeeq M, Murad HAS, Abdallah HM, El-Halawany AM, Protective effect of 6-paradol in acetic acid-induced ulcerative colitis in rats, BMC Complementary Medicine and Therapies 21 (2021) 28, https://doi.org/10.1186/s12906-021-03203-7.
[424] Ajayi BO, Adedara IA, Farombi EO, Pharmacological activity of 6-gingerol in dextran sulphate sodium-induced ulcerative colitis in BALB/c mice, Phytother Res 29 (2015) 566-572, https://doi.org/10.1002/ptr.5286.
[425] Sheng Y, Wu T, Dai Y, Ji K, Zhong Y, Xue Y, The effect of 6-gingerol on inflammatory response and Th17/Treg balance in DSS-induced ulcerative colitis mice, Annals of Translational Medicine 8 (2020) 442, https://doi.org/10.21037/atm.2020.03.141.
[426] Ajayi BO, Adedara IA, Farombi EO, Protective mechanisms of 6-gingerol in dextran sulfate sodium-induced chronic ulcerative colitis in mice, Human & Experimental Toxicology 37 (2018) 1054-1068, https://doi.org/10.1177/0960327117751235.
[427] Li Q, Li K, Hu T, Liu F, Liao S, Zou Y, 6,7-Dihydroxy-2,4-Dimethoxyphenanthrene from Chinese Yam Peels Alleviates DSS-Induced Intestinal Mucosal Injury in Mice via Modulation of the NF-kappaB/COX-2 Signaling Pathway, J Agric Food Chem 69 (2021) 4720-4731, https://doi.org/10.1021/acs.jafc.1c00487.
[428] Wang M, Li Y, Su J, Bai J, Zhao Z, Sun Z, Protective effects of 4-geranyloxy-2,6-dihydroxybenzophenonel on DSS-induced ulcerative colitis in mice via regulation of cAMP/PKA/CREB and NF-kappaB signaling pathways, Phytother Res 37 (2023) 1330-1345, https://doi.org/10.1002/ptr.7689.
[429] Xing J, Sun J, You H, Lv J, Sun J, Dong Y, Anti-inflammatory effect of 3,4-oxo-isopropylidene-shikimic acid on acetic acid-induced colitis in rats, Inflammation 35 (2012) 1872-1879, https://doi.org/10.1007/s10753-012-9509-7.
[430] Yan Y, Wang P, Sun Y, Dong Y, Xing J, Potential Mechanisms of 3, 4-Oxo-Isopropylidene-Shikimic Acid in Ameliorating 2, 4, 6-Trinitrobenzenesulfonic Acid-Induced Colitis in Rats, Journal of Interferon & Cytokine Research 39 (2019) 554-563, https://doi.org/10.1089/jir.2019.0064.
[431] Zheng JY, Xu JY, Zhang L, Wang ZM, Yin XB, Qin LQ, Effect of 3,3'-diselenodipropionic Acid on Dextran Sodium Sulfate-Induced Ulcerative Colitis in Mice, Biol Trace Elem Res 201 (2023) 3961-3970, https://doi.org/10.1007/s12011-022-03491-1.
[432] He X, Liu J, Long G, Xia XH, Liu M, 2,3,5,4'-Tetrahydroxystilbene-2-O-beta-D-glucoside, a major bioactive component from Polygoni multiflori Radix (Heshouwu)

suppresses DSS induced acute colitis in BALb/c mice by modulating gut microbiota, Biomed Pharmacother 137 (2021) 111420, https://doi.org/10.1016/j.biopha.2021.111420.
[433] Wangchuk P, Navarro S, Shepherd C, Keller PA, Pyne SG, Loukas A, Diterpenoid alkaloids of Aconitum laciniatum and mitigation of inflammation by 14-O-acetylneoline in a murine model of ulcerative colitis, Sci Rep 5 (2015) 12845, https://doi.org/10.1038/srep12845.
[434] Zbakh H, Talero E, Avila J, Alcaide A, de Los Reyes C, Zubia E, Motilva V, The Algal Meroterpene 11-Hydroxy-1'-O-Methylamentadione Ameloriates Dextran Sulfate Sodium-Induced Colitis in Mice, Marine Drugs 14 (2016) 149, https://doi.org/10.3390/md14080149.
[435] Cao R, Ma Y, Li S, Shen D, Yang S, Wang X, Cao Y, Wang Z, Wei Y, Li S, Liu G, Zhang H, Wang Y, Ma Y, 1,25(OH)(2) D(3) alleviates DSS-induced ulcerative colitis via inhibiting NLRP3 inflammasome activation, J Leukoc Biol 108 (2020) 283-295, https://doi.org/10.1002/JLB.3MA0320-406RR.
[436] Zhang H, Wu H, Liu L, Li H, Shih DQ, Zhang X, 1,25-dihydroxyvitamin D3 regulates the development of chronic colitis by modulating both T helper (Th)1 and Th17 activation, APMIS 123 (2015) 490-501, https://doi.org/10.1111/apm.12378.
[437] Zeng J, Zhang D, Wan X, Bai Y, Yuan C, Wang T, Yuan D, Zhang C, Liu C, Chlorogenic Acid Suppresses miR-155 and Ameliorates Ulcerative Colitis through the NF-kappaB/NLRP3 Inflammasome Pathway, Mol Nutr Food Res 20 (2020) e2000452, https://doi.org/10.1002/mnfr.202000452.
[438] Gao W, Wang C, Yu L, Sheng T, Wu Z, Wang X, Zhang D, Lin Y, Gong Y, Chlorogenic Acid Attenuates Dextran Sodium Sulfate-Induced Ulcerative Colitis in Mice through MAPK/ERK/JNK Pathway, Biomed Res Int 2019 (2019) 6769789, https://doi.org/10.1155/2019/6769789.
[439] Zhang Z, Wu X, Cao S, Cromie M, Shen Y, Feng Y, Yang H, Li L, Chlorogenic Acid Ameliorates Experimental Colitis by Promoting Growth of Akkermansia in Mice, Nutrients 9 (2017) 677, https://doi.org/10.3390/nu9070677.
[440] Niu W, Chen Y, Wang L, Li J, Cui Z, Lv J, Yang F, Huo J, Zhang Z, Ju J, The combination of sodium alginate and chlorogenic acid enhances the therapeutic effect on ulcerative colitis by the regulation of inflammation and the intestinal flora, Food Funct 13 (2022) 10710-10723, https://doi.org/10.1039/d2fo01619b.
